# Development and Validation of a CT-Based Radiomics Nomogram in Patients With Anterior Mediastinal Mass: Individualized Options for Preoperative Patients

**DOI:** 10.3389/fonc.2022.869253

**Published:** 2022-07-08

**Authors:** Zhou Zhou, Yanjuan Qu, Yurong Zhou, Binchen Wang, Weidong Hu, Yiyuan Cao

**Affiliations:** ^1^ Department of Radiology, Zhongnan Hospital of Wuhan University, Wuhan, China; ^2^ Department of Thoracic Surgery, Zhongnan Hospital of Wuhan University, Wuhan, China

**Keywords:** anterior mediastinal mass, radiomics, nomogram, thymectomy, computed tomography

## Abstract

**Background:**

To improve the preoperative diagnostic accuracy and reduce the non-therapeutic thymectomy rate, we established a comprehensive predictive nomogram based on radiomics data and computed tomography (CT) features and further explored its potential use in clinical decision-making for anterior mediastinal masses (AMMs).

**Methods:**

A total of 280 patients, including 280 with unenhanced CT (UECT) and 241 with contrast-enhanced CT (CECT) scans, all of whom had undergone thymectomy for AMM with confirmed histopathology, were enrolled in this study. A total of 1,288 radiomics features were extracted from each labeled mass. The least absolute shrinkage and selection operator model was used to select the optimal radiomics features in the training set to construct the radscore. Multivariate logistic regression analysis was conducted to establish a combined clinical radiographic radscore model, and an individualized prediction nomogram was developed.

**Results:**

In the UECT dataset, radscore and the UECT ratio were selected for the nomogram. The combined model achieved higher accuracy (AUC: 0.870) than the clinical model (AUC: 0.752) for the prediction of therapeutic thymectomy probability. In the CECT dataset, the clinical and combined models achieved higher accuracy (AUC: 0.851 and 0.836, respectively) than the radscore model (AUC: 0.618) for the prediction of therapeutic thymectomy probability.

**Conclusions:**

In patients who underwent UECT only, a nomogram integrating the radscore and the UECT ratio achieved good accuracy in predicting therapeutic thymectomy in AMMs. However, the use of radiomics in patients with CECT scans did not improve prediction performance; therefore, a clinical model is recommended.

## Introduction

Mediastinal masses are uncommon compared to masses in the lungs. The prevalence of mediastinal masses ranges from 0.73% to 0.9%, taking reference from a population-based cohort study (1) and two lung cancer screening studies (2, 3). The masses most commonly occur in the anterior mediastinum, accounting for 50%–69.8% of all mediastinal masses (4, 5). Anterior mediastinal masses (AMMs) include a wide range of pathological entities, varying from benign cysts to neoplasms (benign and malignant) (6, 7). Therefore, they often pose a diagnostic challenge for clinicians (8).

Surgical excision is one of the most common treatments for AMMs (9), which may not necessarily be appropriate. The overall non-therapeutic thymectomy rate ranges from 22% to 68%, as it had been reported in the literature (10, 11), and is often due to diagnostic inaccuracies. For example, masses that did not warrant surgical intervention were misdiagnosed as thymomas (10). Thus, a definitive diagnosis is crucial for better preoperative counseling, appropriate treatment decisions, and follow-up management.

Biopsy of AMMs is an invasive approach to obtain tissue before surgical intervention and treatment for histopathological analysis (12, 13). However, not all patients are eligible for biopsy given the presence of certain comorbidities as well as lesion size and location (8, 14). On the other hand, there are also cases where direct surgical resection can be performed based only on imaging and clinical features, bypassing the superfluous step of biopsy (8). Imaging examination, as a non-invasive approach, is indispensable for preoperative workup and is essential for the differential diagnosis, staging, and follow-up monitoring of AMMs (15). CT is universally available for routine preoperative preparation and remains the current modality of choice. Nevertheless, the average diagnostic accuracy only ranged from 35% to 78% when radiologists provided the diagnosis based on their understanding and judgment of demographic and CT imaging features (5, 16, 17). The discriminating ability for malignant germ cell tumors (35%), thymic carcinomas (38%), and cysts (46%) is not satisfactory (5). Thus, there is an urgent need to improve the radiological diagnostic accuracy of AMMs to reduce the chances of unnecessary surgery for individuals who are unlikely to benefit from it.

Radiomics is an emerging translational field of research aimed at extracting features, more than those observed by radiologists, from radiological images for clinical decision-making (18). Most radiomics studies investigated thymic epithelial tumors (TETs) (19–21), the most prevalent primary tumor in the anterior mediastinum that accounts for 47% of the total mediastinal tumors (22). Quantitative radiomics analysis based on CT, MRI, and PET/CT has been conducted and has shown good diagnostic performance in differentiating tumor subtypes, staging, invasiveness, and risk categorization in TETs (19, 21, 23, 24). However, there is no empirical evidence proving that CT-based radiomics analysis would be beneficial for reducing the non-therapeutic thymectomy rate in AMMs. As such, this is an interesting problem that requires further investigation.

In this study, we sought to evaluate the potential value of CT-based radiomics features by establishing a comprehensive predictive nomogram that aims to improve preoperative diagnostic accuracy and reduce the non-therapeutic thymectomy rate. We also investigated the radiomics features extracted using different CT imaging techniques, including unenhanced CT (UECT) and contrast-enhanced CT (CECT), and explored their potential use in the clinical decision-making regarding AMMs.

## Materials and Methods

### Patient Selection

**Figure 1:**
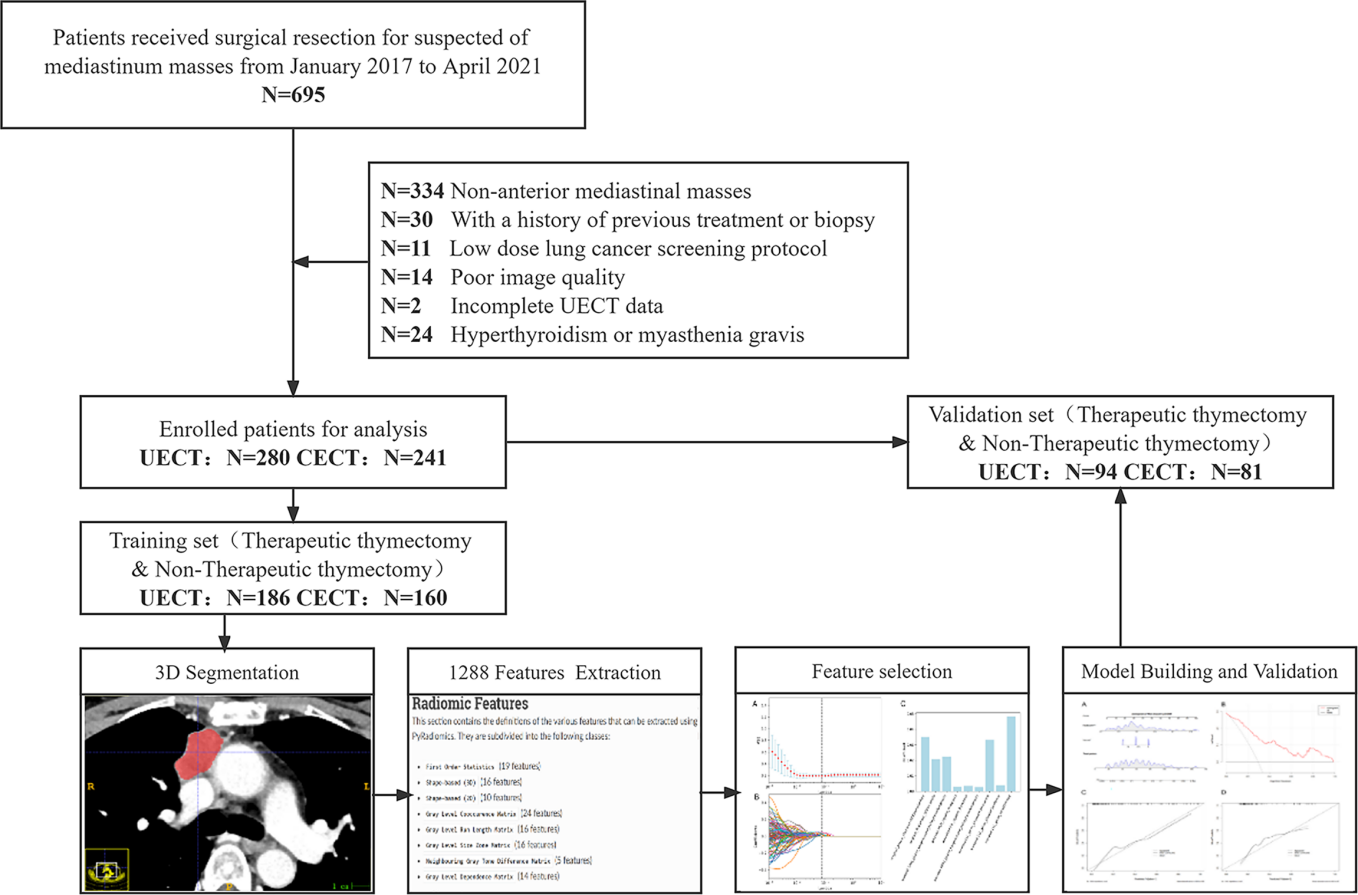
Flowchart of the patient recruitment pathway. The flowchart shows how the study population has been selected and its retrospective manner. *N*, number; UECT, unenhanced computed tomography; CECT, contrast-enhanced computed tomography.

This retrospective, single-center study was approved by our institutional ethics committee (no. 2022048 K), which waived the need for informed consent. The workflow diagram of the analysis is shown in **Figure 1**.

We searched the electronic medical record system of our hospital for patients (*n* = 695) who underwent surgical resection of suspected mediastinal masses at our institution from January 2017 to April 2021. The inclusion criteria were as follows: 1) AMM, 2) underwent UECT and/or CECT within 1 month before surgery, and 3) available clinical data and surgical records. The exclusion criteria were as follows: 1) previous treatment or biopsy before the CT scan, 2) low-dose lung cancer screening or lung nodule follow-up CT scan protocols, 3) poor image quality due to severe respiratory motion artifacts or other reasons, 4) incomplete UECT data, and 5) hyperthyroidism or myasthenia gravis. AMM was defined as any mass no less than 5 mm in the short-axis diameter that was located in the anterior mediastinum as defined by the International Thymic Malignancy Interest Group (25, 26). The center method was used for defining the mass center on axial CT images and further locating the theoretical site of mass origin (25, 26). When multiple masses were present in a single patient, they were evaluated separately.

In total, 280 patients with 280 UECT and 241 CECT scans were enrolled in this retrospective study. Clinical information, including age, sex, body mass index (BMI), and pathological diagnosis of all cases, was obtained from our institutional medical record system. The median time from the preoperative CT scan to surgery was 7.5 days (interquartile range, IQR: 3–10) in UECT and 6 days (IQR: 3–9) on CECT.

### Pathological Analysis

Surgical resection generally refers to total thymectomy or total thymectomy with partial en bloc resection of adjacent structures when complete resection is necessary. The procedures included open thoracotomy, mediastinoscopy, or robot-assisted mediastinoscopy. All resected specimens were formalin-fixed and hematoxylin–eosin-stained according to standard procedures. The pathological diagnosis was independently performed by two pathologists who were blinded to the radiological diagnosis. The diagnosis was made and reported according to the classification criteria issued in the 4th edition of the WHO classification of tumors of the thymus in 2015 (27).

### Image Acquisition

CT imaging was performed using one of four 64-slice multidetector CT scan machines (Philips Brilliance CT 64; Philips Medical Systems, Eindhoven, Netherlands), Philips IQon spectral CT (Philips Medical Systems, Eindhoven, Netherlands), GE Discovery CT750 HD (GE Medical Systems, Waukesha, USA), and Somatom Definition Flash (Siemens Healthineers, Erlangen, Germany). Detailed acquisition and reconstruction protocol specifications are provided in **Supplementary Material 1**. All CT scans were performed over the entire thorax, in the supine position, at the end of the inspiratory phase. CECT was obtained 60 s after contrast agent administration. The contrast agent (Omnipaque 350, GE Healthcare, Waukesha, USA) was intravenously administered at a dose of 1.5 ml/kg body weight and a rate of 3.0 ml/s *via* a power injector, followed by a 20.0-ml saline flush.

### Mass Analysis and Segmentation

Location (unilateral or bilateral), size (maximum axial diameter), shape (regular, irregular), boundary (clear, indistinct), mediastinal fatty line (preserved, infiltrate), fatty component (absence, presence), calcification (absence, presence), pericardial effusion (absence, presence), pleural effusion (absence, presence), and enhancement homogeneity (homogeneous, inhomogeneous) were verified by two radiologists (YZ and ZZ, both with 6 years of experience in thoracic imaging diagnosis) who were blinded to the pathological diagnosis. Subsequently, two attenuation-to-background ratios were calculated for each mass:


$$\text{ UECT ratio }=\;\frac{{\text{ AMM attenuation}}_{\text{UECT}}}{{\text{ Pectoralis major attenuation}}_{\text{UECT}}}$$

$$\text{CECT ratio }=\;\frac{{\text{AMM attenuation}}_{\text{UECT}}\;-{\text{ AMM attenuation}}_{\text{CECT}}}{{\text{Pectoralis major attenuation}}_{\text{UECT}}-{\text{ Pectoralis major attenuation}}_{\text{CECT}}}$$

All CT findings were evaluated based on the mediastinal window setting on the transverse plain CT scan section (window width, 300 HU; window level, 30 HU).

The included masses were independently segmented by two radiologists (YZ and ZZ) using the open-source image processing software ITK-SNAP 3.8.0 (www.itksnap.org) (28). A three-dimensional mask, defined as the delineation around the mass border for every CT axial plane, was delineated manually. The original digital imaging in communications in medicine format and the segmentation mask were exported directly into neuroimaging informatics technology initiative (NIfTI) format after the above segmentation process.

### Radiomics Feature Extraction

Referring to the recommended standardized radiomics analysis workflow (18), segmentation data were analyzed using Pyradiomics (version 3.0.1 https://pyradiomics.readthedocs.io/) in Python (version 3.7 https://www.python.org/) to extract the radiomics features (29). The NIfTI format data were resampled into 1.0 × 1.0 × 1.0-mm^3^ voxels using a nearest-neighbor algorithm. In total, 1,288 radiomics features were extracted, and the specific classifications were as follows: 1) first-order statistics and filter-based features (*n* = 252), 2) shape (*n* = 14), 3) gray level co-occurrence matrix and filter-based features (*n* = 308), 4) gray level run length matrix and filter-based features (*n* = 224), 5) gray level size zone matrix and filter-based features (*n* = 224), 6) gray level dependence matrix and filter-based features (*n* = 196), and 7) neighboring gray tone difference matrix and filter-based features (*n* = 70). All extracted radiomics features are listed in **Supplementary Material 2**. In addition to the shape features, all the features were computed on the original image or on a Gaussian- or wavelet-filtered image. Most features were defined in compliance with the imaging biomarker standardization initiative. The bin size in our analysis is 25.

### Radiomics Feature Selection and Predictive Model Building

The CECT and UECT datasets were randomly divided into training and validation sets in a ratio of 2:1. The median padding method was used to fill in missing values and replace outliers, after which the standardized data were subsequently used for statistical analyses. Radiomics features from the training set were selected using the Mann–Whitney test or independent Student’s *t*-test when results achieved *p <*0.05. The selected features were further filtered using least absolute shrinkage and selection operator (LASSO) with 10-fold cross-validation. Clinical and radiographic features were analyzed using the Mann–Whitney test, independent Student’s *t*-test, or chi-square test when appropriate. Countable data were analyzed with the chi-square test, data that fit the normal distribution were analyzed with the independent Student’s *t*-test, and data that did not fit the normal distribution were analyzed using the Mann–Whitney test.

The receiver operating characteristic (ROC) curve was used to evaluate the predictive performance of the radscore (radiomics feature) model, clinical model (clinical and radiographic features), and combined model (clinical + radscore model) in the training and validation sets. The Youden index was calculated, and the score at the maximum Youden index was taken as the cutoff value. An individualized prediction nomogram was constructed.

### Statistical Analysis

The intraclass correlation coefficient (ICC) was used to assess interobserver agreement during the segmentation process. Features with an ICC greater than 0.90 were retained for further statistical analysis. LASSO, Mann–Whitney test, independent Student’s *t*-test, chi-square test, and multivariate logistic analyses were performed to select the clinical, radiographic, and radiomics features. *p*-value was adjusted for multiple comparisons in the radiomics feature selection step as false discovery rate (FDR)-corrected *q*-value with a significance level of 0.05. Other statistical tests were two-sided with a significance level of 0.05. All statistical analyses were performed using the R software (version 4.0.5 https://www.r-project.org/) with “readr,” “irr,” “rms,” “foreign,” “Matrix,” “Hmisc,” “rmda,” “ggprism,” “ggDCA,” “ggplot2,” “ggsci,” “glmnet,” “fdrtool,” and “regplot” packages.

## Results

### Clinical and Radiographic Features

A total of 280 patients with 280 UECT and 241 CECT scans were recruited for this study. The pathological characteristics of the patients are shown in **Table 1**. Around 92.14% (258/280) of the masses were completely resected. The non-therapeutic thymectomy rates were 51.07% (143/280) and 48.55% (117/241) in the UECT and CECT datasets, respectively.

**Table 1:** Pathology characteristics of patients in UECT and CECT.

Diagnoses	UECT (*n* = 280)	NECT (*n* = 241)
Therapeutic thymectomy		
WHO type A thymoma	8	7
WHO type AB thymoma	23	20
WHO type B1 thymoma	12	10
WHO type B2 thymoma	22	22
WHO type B3 thymoma	14	13
Thymic carcinoma	22	22
Lymphoma	4	4
Small cell carcinoma	4	2
Atypical carcinoid	2	2
Castleman’s disease	3	2
Sarcomatoid carcinoma	3	3
Metastatic lymphoma node	2	2
Thyroid carcinoma	1	1
Seminoma	3	2
Yolk sac tumor	2	2
Teratoma	10	9
Aggressive fibromatosis	1	1
Solitary fibrous tumor	1	0
Total	137	124
Non-therapeutic thymectomy		
Cyst	110	89
Thymic hyperplasia	15	13
Hemangioma	5	3
Tuberculosis	5	4
Sarcoidosis	3	3
Eutopic thyroid	1	1
Lymphoma node	1	1
Fibrosing mediastinitis	3	3
Total	143	117

Data are the number of masses. UECT, unenhanced computed tomography; CECT, contrast-enhanced computed tomography; WHO, World Health Organization.

In the UECT dataset, diameter, shape, boundary, mediastinal fatty line, and UECT ratio showed a statistically significant difference (*p* < 0.05) between the non-therapeutic thymectomy and therapeutic thymectomy groups in both the training and validation sets, while sex, pleural effusion, and calcification exhibited evident differences in either the training or validation set (**Table 2**).

**Table 2:** The clinical and radiographic features in the training and validation sets of UECT.

Characteristics	Training set (*n* = 186)			Validation set (*n* = 94)		
	Therapeutic thymectomy (*n* = 91)	Non-therapeutic thymectomy (*n* = 95)	*p*-value	Therapeutic thymectomy (*n* = 46)	Non-therapeutic thymectomy (*n* = 48)	*p*-value
Age, years	56.5 (46.1, 63.5)	53.4 (45.7, 60.6)	0.181	57.4 (47.1, 64.7)	57.0 (49.2, 64.9)	0.464
Gender			**0.006**			0.833
Female	41 (39.8%)	62 (60.2%)		24 (52.2%)	24 (50.0%)	
Male	50 (60.2%)	33 (39.8%)		22 (47.8%)	24 (50.0%)	
BMI, kg/m^2^	22.4 (20.7, 24.3)	23.1 (21.2, 25.2)	0.300	22.3 (20.6, 24.1)	22.7 (20.1, 25.5)	0.560
Diameter, mm	54.3 (39.0, 70.6)	36.0 (23.0, 54.7)	**<0.001**	52.2 (35.8, 69.8)	24.0 (18.8, 46.9)	**<0.001**
UECT ratio	0.86 (0.73, 0.95)	0.57 (0.24, 0.84)	**<0.001**	0.81 (0.63, 0.92)	0.61 (0.29, 0.86)	**0.008**
Location			0.464			0.064
Unilateral	43 (47.3%)	50 (52.6%)		19 (41.3%)	30 (62.5%)	
Bilateral	48 (52.8%)	45 (47.4%)		27 (58.7%)	18 (37.5%)	
Shape			**<0.001**			**0.016**
Regular	65 (71.4%)	88 (92.6%)		33 (71.7%)	44 (91.7%)	
Irregular	26 (28.6%)	7 (7.4%)		13 (28.3%)	4 (8.3%)	
Boundary			**0.013**			**0.026**
Clear	78 (85.7%)	92 (96.8%)		37 (80.4%)	46 (95.8%)	
Indistinct	13 (14.3%)	3 (3.2%)		9 (19.6%)	2 (4.2%)	
Mediastinal fatty line			**<0.001**			**0.001**
Preserve	70 (76.9%)	93 (97.9%)		32 (69.6%)	47 (97.9%)	
Infiltrate	21 (23.1%)	2 (2.1%)		14 (30.4%)	1 (2.1%)	
Fatty component			0.604			0.481
Absence	86 (94.5%)	88 (92.6%)		41 (89.1%)	45 (93.7%)	
Presence	5 (5.5%)	7 (7.4%)		5 (10.9%)	3 (6.3%)	
Calcification			**0.018**			0.165
Absence	61 (67.0%)	81 (85.3%)		36 (78.3%)	43 (89.6%)	
Present	30 (33.0%)	14 (14.7%)		10 (21.7%)	5 (10.4%)	
Pleural effusion			0.242			**0.005**
Absence	86 (94.5%)	93 (97.9%)		39 (84.78%)	48 (100.0%)	
Present	5 (5.5%)	2 (2.1%)		7 (15.22%)	0(0.0%)	
Pericardial effusion			0.983			0.113
Absence	87 (95.6%)	95 (100.0%)		43 (93.5%)	48 (100.0%)	
Presence	4 (4.4%)	0 (0.0%)		3 (6.5%)	0 (0.0%)	

Enumeration data are the number of masses with percentage in parentheses, and measurement data are expressed as median and interquartile range (IQR). Bold p-values <0.05.

BMI, body mass index; UECT, unenhanced computed tomography.

Furthermore, multivariate analysis revealed significant differences in diameter, calcification, and UECT ratio. These three features were subsequently selected to establish the UECT clinical model.


Calculation formula=−3.333043+0.025668×diameter+1.008648×calcification+2.825093×UECT ratio

**Figure 2:**
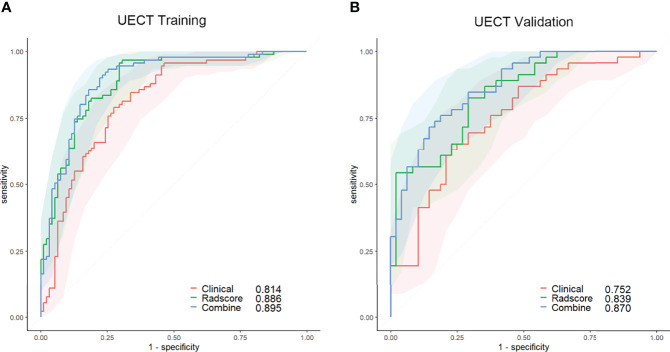
ROC curves of the clinical (red lines), radscore (green lines), and combined (blue lines) models in the training set **(A)** and validation set **(B)** of the UECT model. The shaded areas represent the confidence intervals of the ROC curves. UECT, unenhanced computed tomography; ROC, receiver operating characteristic.

The area under the curve (AUC) of the clinical model was 0.814 (95% CI, 0.751–0.867; threshold: 0.06431; sensitivity: 79.12%; specificity: 72.63%) in the training set and 0.752 (95% CI, 0.653–0.836; threshold: 0.02138; sensitivity: 69.57%; specificity: 70.83%) in the validation set (**Figure 2**).

In the CECT dataset, diameter, shape, mediastinal fatty line, homogeneity, and UECT ratio showed statistical differences (*p* < 0.05) between the non-therapeutic thymectomy and therapeutic thymectomy groups in both the training and validation sets, while boundary, sex, pleural effusion, and calcification exhibited evident differences in either the training or validation set (**Table 3**). Multivariate analysis revealed significant differences in diameter, UECT ratio, and homogeneity. These three features were subsequently selected to establish a clinical CECT model.


Calculation formula=−3.6322+0.01939×diameter−1.77731×homogeneity−2.35451×UECT ratio

**Table 3:** The clinical and radiographic features in the training and validation sets of CECT.

Characteristics	Training set (*n* = 160)			Validation set (*n* = 81)		
	Therapeutic thymectomy (*n* = 82)	Non-therapeutic thymectomy (*n* = 78)	*p*-value	Therapeutic thymectomy (*n* = 42)	Non-therapeutic thymectomy (*n* = 39)	*p*-value
Age, years	56.3 (45.6, 63.3)	53.0 (45.5, 62.2)	0.428	57.7 (41.8, 63.9)	53.2 (45.5, 60.6)	0.643
Gender			0.192			**0.036**
Female	41 (50.0%)	47 (60.3%)		15 (35.7%)	23 (59.0%)	
Male	41 (50.0%)	31 (39.7%)		27 (64.3%)	16 (41.0%)	
BMI, kg/m^2^	22.6 (20.8, 24.4)	23.1 (21.2, 25.5)	0.407	22.2 (20.6, 23.8)	23.3 (21.4, 24.9)	0.050
Diameter, mm	55.9 (41.4, 71.4)	32.5 (22.3, 58.8)	**<0.001**	53.5 (37.2, 68.5)	27.8 (19.6, 45.1)	**<0.001**
UECT ratio	0.84 (0.71, 0.94)	0.63 (0.29, 0.86)	**<0.001**	0.81 (0.63, 0.93)	0.47 (0.24, 0.81)	**<0.001**
CECT ratio	4.22 (1.94, 9.82)	1.28 (0.28, 4.23)	0.180	4.09 (1.47, 9.39)	0.83 (0.27, 2.29)	0.263
Location			0.160			0.429
Unilateral	34 (41.5%)	37 (47.4%)		20 (47.6%)	22 (56.4%)	
Bilateral	48 (58.5%)	41 (52.6%)		22 (52.4%)	17 (43.6%)	
Shape			**0.003**			**<0.001**
Regular	60 (73.2%)	71 (91.0%)		27 (64.3%)	37 (94.87%)	
Irregular	22 (26.8%)	7 (9.0%)		15 (35.7%)	2 (5.13%)	
Boundary			**0.005**			0.058
Clear	67 (81.7%)	75 (96.2%)		35 (83.3%)	38 (97.4%)	
Indistinct	15 (18.3%)	3 (3.85%)		7 (16.7%)	1 (2.6%)	
Mediastinal fatty line			**<0.001**			**<0.001**
Preserve	62 (75.6%)	76 (97.4%)		29 (69.1%)	38 (97.4%)	
Infiltrate	20 (24.4%)	2 (2.6%)		13 (30.9%)	1 (2.6%)	
Fatty component			0.766			0.093
Absence	75 (91.5%)	73 (93.6%)		39 (92.9%)	35 (89.7%)	
Presence	7 (8.5%)	5 (6.4%)		3 (7.1%)	4 (10.3%)	
Calcification			**0.020**			0.145
Absence	54 (65.9%)	64 (82.1%)		32 (76.2%)	35 (89.7%)	
Present	28 (34.1%)	14 (17.9%)		10 (23.8%)	4 (10.3%)	
Pleural effusion			**<0.001**			1.000
Absence	71 (86.6%)	78 (100.0%)		41 (97.6%)	39 (100.0%)	
Present	11 (13.4%)	0 (0.0%)		1 (2.4%)	0(0.0%)	
Pericardial effusion			0.059			0.494
Absence	77 (93.9%)	78 (100.0%)		40 (95.2%)	39 (100.0%)	
Present	5 (6.1%)	0 (0.0%)		2 (4.8%)	0(0.0%)	
Homogeneity			**<0.001**			**<0.001**
Homogeneous	25 (30.5%)	65 (83.3%)		19 (45.2%)	35 (89.7%)	
Inhomogeneous	57 (69.5%)	13 (16.7%)		23 (54.8%)	4 (10.3%)	

Enumeration data are the number of masses with percentage in parentheses, and measurement data are expressed as median and interquartile range (IQR). Bold p-values <0.05.

BMI, body mass index; CECT, contrast-enhanced computed tomography.

**Figure 3:**
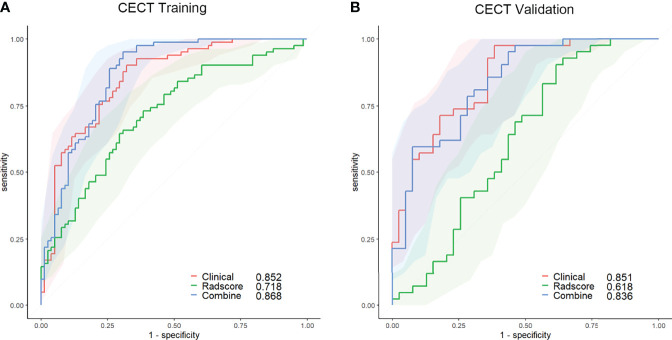
ROC curves of the clinical (red lines), radscore (green lines), and combined (blue lines) models in the training set **(A)** and validation set **(B)** of the CECT model. The shaded areas represent the confidence intervals of the ROC curves. CECT, contrast-enhanced computed tomography; ROC, receiver operating characteristic.

The AUC of the clinical model was 0.852 (95% CI, 0.793–0.911; threshold: −0.92923; sensitivity: 90.24%; specificity: 67.95%) in the training set and 0.851 (95% CI, 0.769–0.933; threshold: −1.62975; sensitivity: 97.62%; specificity: 61.54%) in the validation set (**Figure 3**).

### Radiomics Feature Selection

**Figure 4:**
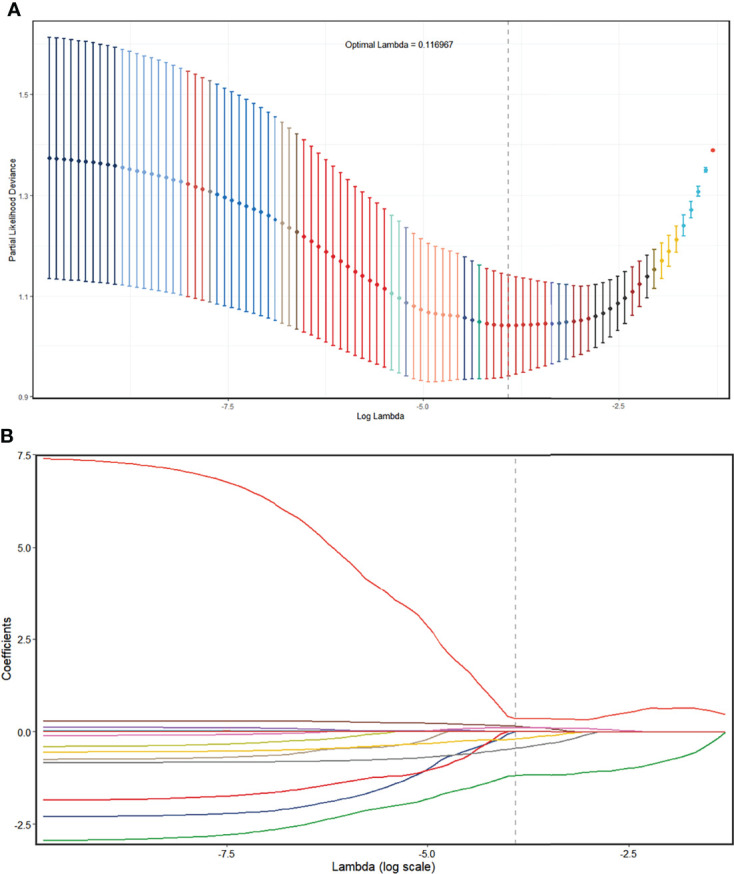
Radiomics feature selection using the LASSO regression model in the UECT model. **(A)** The partial likelihood deviance from the LASSO regression cross-validation procedure was plotted against log(λ). The optimal λ value of 0.116967 was selected. **(B)** LASSO coefficient profiles of the radiomics features. As the tuning parameter (λ) increased using 10-fold cross-validation, more coefficients tended to approach 0 and the optimal non-zero coefficients were generated, which yielded a set of the optimal radiomics features. LASSO, least absolute shrinkage and selection operator; UECT, unenhanced computed tomography.

In the UECT dataset, 587 features with good agreement (ICC > 0.90) were selected for further reduction (**Supplementary Material 3**). A total of 564 features were removed due to lack of statistical difference in the Mann–Whitney test, and 20 features were removed due to high correlation with other features in LASSO selection, with four features remaining (**Supplementary Material 4** **;** **Figure 4**). Consequently, the selected features were subjected to a radscore model:


$$\begin{array}{c} \text{Calculation formula}=-0.206691567+0.394716773\times \text{wavelet}.\text{HLH}\_\text{glrlm}\_\text{RunEntropy}\\ +0.003935499\\ \times \text{log}.\text{sigma}.5.0.\text{mm}.3\text{D}\_\text{glszm}\_\text{SmallAreaHighGrayLevelEmphasis}\\ +0.014854691\times \text{log}.\text{sigma}.1.0.\text{mm}.3\text{D}\_\text{glcm}\_\text{JointAverage}-5.008866283\\ \times \text{original}\_\text{shape}\_\text{SurfaceVolumeRatio} \end{array}$$

The AUC of the radscore model was 0.886 (95% CI, 0.831–0.928; threshold: 0.40102; sensitivity: 96.70%; specificity: 69.47%) in the training set and 0.839 (95% CI, 0.749–0.907; threshold: 0.47809; sensitivity: 82.61%; specificity: 70.83%) in the validation set (**Figure 2**).

**Figure 5:**
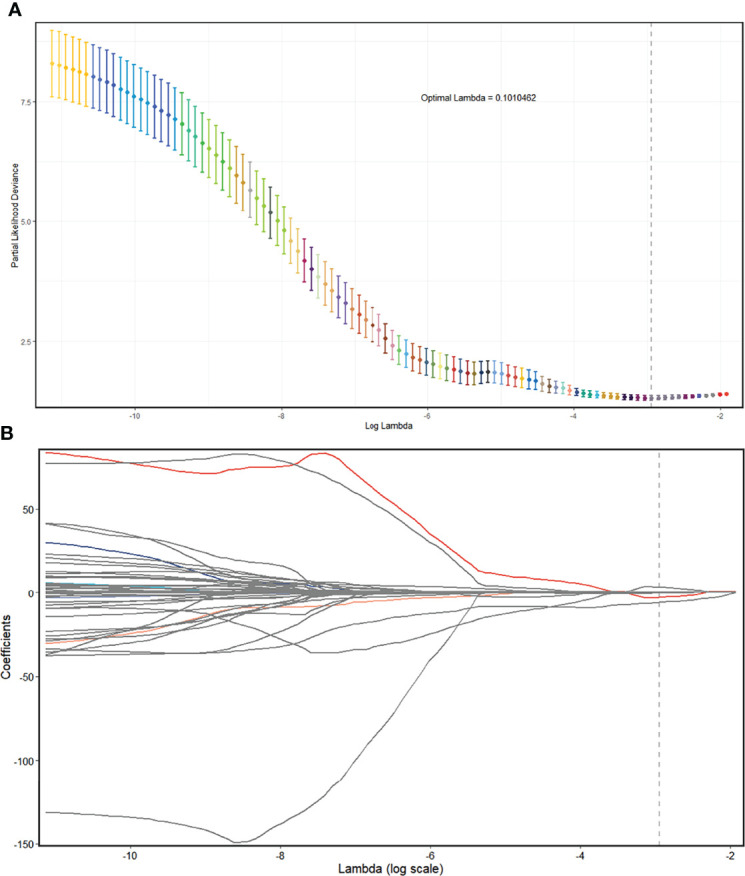
Radiomics feature selection using the LASSO regression model in the CECT model. **(A)** The partial likelihood deviance from the LASSO regression cross-validation procedure was plotted against log(λ). The optimal λ value of 0.1010462 was selected. **(B)** LASSO coefficient profiles of the radiomics features. As the tuning parameter (λ) increased using 10-fold cross-validation, more coefficients tended to approach 0 and the optimal non-zero coefficients were generated, which yielded a set of the optimal radiomics features. LASSO, least absolute shrinkage and selection operator; CECT, contrast-enhanced computed tomography.

In the CECT dataset, 846 features with good agreement (ICC > 0.90) were selected for further reduction (**Supplementary Material 3**). A total of 712 features were removed due to lack of statistical difference in the Mann–Whitney test, and 129 features were removed due to their high correlation with other features, with five remaining features (**Supplementary Material 5** **;** **Figure 5**). Consequently, the selected features were subjected to a radscore model:


$$\begin{array}{c} \text{Calculation formula}=-4.67265+0.000380729\\ \times \text{wavelet}.\text{HHL}\_\text{glrlm}\_\text{LongRunHighGrayLevelEmphasis}+4.73167\\ \times \text{log}.\text{sigma}.4.0.\text{mm}.3\text{D}\_\text{glcm}\_\text{Idmn}-15.76711\times \text{wavelet}.\text{HLL}\_\text{ngtdm}\_\text{Contrast}\\ -0.1337031\times \text{log}.\text{sigma}.5.0.\text{mm}.3\text{D}\_\text{gldm}\_\text{LowGrayLevelEmphasis}\\ +0.01050467\times \text{log}.\text{sigma}.2.0.\text{mm}.3\text{D}\_\text{glcm}\_\text{JointAverage} \end{array}$$

The AUC of the radscore model was 0.718 (95% CI, 0.639–0.797; threshold: 0.07717; sensitivity: 64.63%; specificity: 70.51%) in the training set and 0.618 (95% CI, 0.492–0.745; threshold: −0.02176; sensitivity: 90.48%; specificity: 38.46%) in the validation set (**Figure 3**).

### Construction of the Prediction Nomogram and Clinical Utility

**Figure 6:**
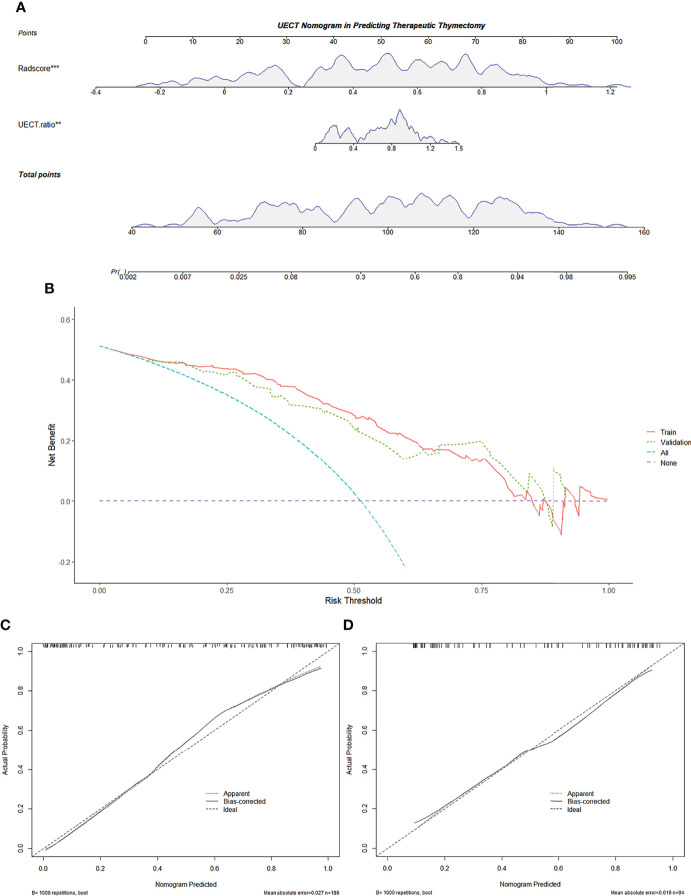
Radiomics-based nomogram was developed in the training set for the UECT model. **(A)** Radscore and the UECT ratio were incorporated. **(B)** Decision curve of the nomogram. The *y*-axis represents the net benefit. The *x*-axis represents the threshold probability. The red line represents the radiomics-based nomogram of the training set. The green line represents the radiomics-based nomogram of the validation set. The blue line represents the assumption that all AMMs are fit for therapeutic thymectomy. The violet line represents the assumption that all AMMs are not fit for therapeutic thymectomy. The decision curve demonstrates that if the threshold probability is >5%, using the nomogram for therapeutic thymectomy adds more benefit than predicting either all or no patients. **(C)** Calibration curve of the radiomics-based nomogram in the training set. **(D)** Calibration curve of the radiomics-based nomogram in the validation set. The 45-degree lines represent perfect predictions. The black dotted lines represent the predictive performance of the nomogram. A closer fit to the 45-degree line represents a better prediction. AMM, anterior mediastinal mass; UECT, unenhanced computed tomography.

In the UECT dataset, diameter, calcification, and UECT ratio were incorporated into the multivariate logistic analysis to develop a prediction nomogram (**Figure 6**). As a result, the UECT ratio and radscore were selected as independent predictors of therapeutic thymectomy.


Calculation formula=−4.52179+1.78133×UECT ratio+7.75105×Radscore

The calibration curve of the nomogram for predicting the probability of AMM suitable for therapeutic thymectomy demonstrated an acceptable agreement between prediction and observation in the training and validation sets (**Figure 6**). The AUC of the combined model was 0.895 (95% CI, 0.842–0.935; threshold: 0.15017; sensitivity: 93.41%; specificity: 74.74%) in the training set and 0.870 (95% CI, 0.784–0.930; threshold: 0.61539; sensitivity: 76.09%; specificity: 81.25%) in the validation set (**Figure 2**). The decision curve analysis of the nomogram is presented in **Figure 6**.

The radscore and the combined model achieved a higher accuracy (AUC: 0.886 and 0.895, respectively) than the clinical model (AUC: 0.814) for the prediction of therapeutic thymectomy probability among patients with AMMs in the UECT training set (Delong test; *p* = 0.019 and 0.001, respectively). A difference was observed only between the combined and clinical models in the validation set (*p* = 0.005).

In the CECT dataset, the UECT ratio and homogeneity were incorporated with the radscore into the multivariate logistic analysis and were selected for developing the prediction nomogram:


Calculation formula=−2.6841+2.2589×UECT ratio+2.2589 ×homogeneity+3.3255×Radscore

The calibration curve of the nomogram for predicting the probability of AMM suitable for therapeutic thymectomy demonstrated an acceptable agreement between prediction and observation in the training and validation sets. The AUC of the combined model was 0.868 (95% CI, 0.805–0.916; threshold: −0.83458; sensitivity: 95.12%; specificity: 69.13%) in the training set and 0.836 (95% CI, 0.738–0.909; threshold: 0.71154; sensitivity: 59.52%; specificity: 95.31%) in the validation set (**Figure 3**).

The clinical and combined models achieved a higher accuracy (AUC: 0.852 and 0.868, respectively) than the radscore model (AUC: 0.718) for the prediction of therapeutic thymectomy probability among patients with AMMs in the CECT training set (Delong test; *p* < 0.001 and 0.004, respectively). Similar results were obtained for the validation set.

## Discussion

In this retrospective study, we developed and validated two radiomic nomograms for preoperative AMMs. The nomogram demonstrated an individualized selection of AMMs, which could help in screening patients who required surgical intervention.

For chest UECT-identified AMMs, follow-up diagnosis was always based on radiological features on CECT or MRI, and clinicians tended to choose surgical intervention as the first management strategy. The non-therapeutic thymectomy rate was as high as 51.07% (143/280) in our study, which is in line with previous studies (22%–68%) (10, 11). Unlike in previous studies (10), lymphoma was assigned to the therapeutic thymectomy group in our study. We considered that routine follow-up may not be justified for this disease since therapeutic thymectomy could help determine the pathological diagnosis, which is essential for subsequent treatments. There has been increased awareness of non-therapeutic thymectomy cases, and a more accurate preoperative diagnosis contributes to avoiding them.

In the current study, we mainly focused on UECT and CECT, which are the most common imaging examinations in routine preoperative preparation. Usually, in cases of renal insufficiency, CECT may be replaced with MRI or saved with only UECT. The value of the UECT clinical model was limited according to the AUC (training: 0.814, validation: 0.752), whereas the CECT clinical model was found to be acceptable (AUC, training: 0.852, validation: 0.851). In our study, patients who were suitable for surgical resection presented with larger diameters and larger UECT ratios and were inhomogeneous. Diameter is a readily accessible observable metric for the clinical diagnosis of AMMs, and this finding is consistent with that of a previous study (30). Previous studies have mostly focused on distinguishing between benign and malignant AMMs (30, 31). In our study, the therapeutic thymectomy group consisted mostly of malignant masses, which may explain the diameter result. In previous studies, benign masses had lower attenuation than malignant masses (16, 31). To reduce the risk of bias, attenuation was substituted by the UECT ratio in our study; therefore, our results also fit well with previous studies on this aspect. Cysts (76.1%, 89/117) were the most common low attenuation masses in the non-therapeutic thymectomy group, which might be the reason why we found the UECT ratio larger in the therapeutic thymectomy group. Mass enhancement patterns are typically associated with necrosis, cystic changes, and heterogeneity. TETs formed the largest proportion, approximately 75.8% (94/124), of the therapeutic thymectomy group. The inhomogeneity rate of TETs was approximately 67% versus 0% for cysts, as reported in the literature (10). The different disease distributions likely resulted in different radiological characteristics. We also explored other possible reasons for these silent radiological features. Unilateral location, lobulation, and fewer fatty components are commonly used to identify TETs (10, 16). However, all three features failed to achieve statistical significance in this study. As such, these results may be attributed to our grouping method. Teratomas, which occur in a central location, are oval or rounded, contain a fatty component, and require surgical intervention because of the risk of developing malignant change. It was also categorized into the therapeutic thymectomy group. This may partially explain the negative results obtained.

To construct the radiomics-based radscore, we screened 1,288 candidate radiomics features on both the UECT and CECT datasets. Radiomics analyses yielded results that differed from those of the clinical models. The UECT radscore model (AUC: training: 0.886, validation: 0.839) was more effective than the CECT score model (AUC: training: 0.718, validation: 0.618). There are some controversies regarding the diagnostic efficacy of UECT and CECT radiomics modeling. Yasaka et al. (32) studied solid AMMs and cysts using quantitative CT texture analyses and found that CECT (AUC: 0.983) was more effective than UECT (AUC: 0.780). Similar results were obtained in a study by Wang et al. that focused on risk categorization and clinical staging of thymomas (33). However, our results are similar to those of Sui et al., who showed that the radiomics features of UECT performed better than those of CECT in the prediction of anterior mediastinal lesion risk grading (34). This discrepancy could have been caused by a grouping bias. Another possible explanation is that the biological heterogeneity within the tumor that can be characterized by radiomics is confounded by contrast material (35). SurfaceVolumeRatio is one of the four target features selected and used to construct the UECT radscore model. It had a lower median value in the therapeutic thymectomy group, indicating that masses tended to have a more compact shape in this group. JointAverage had a higher median value in the therapeutic thymectomy group, reflecting mass attenuation. RunEntropy and SmallAreaHighGrayLevelEmphasis were utilized to evaluate heterogeneity, which resulted in higher median values in the therapeutic thymectomy group. This suggests that the heterogeneity of masses in this group was more apparent.

A comparison of the predictive power of the clinical, radscore, and combined models was performed in our study. In the UECT dataset, the predictive performance of the combined model (AUC: 0.870) was higher than that of the clinical model (AUC: 0.754; *p* = 0.005). Although there was no significant difference between the clinical and radscore models (AUC: 0.839; *p* = 0.093), the AUC of the radscore model was higher. This implies that incorporating the radscore to develop a combined model may improve the predictive performance for the therapeutic thymectomy ratio compared to using the UECT clinical model only. Therefore, we adopted a nomogram that presented quantitative differences, which is more accessible than the calculation formula. The nomogram combining radscore and UECT ratio demonstrated satisfactory calibration and discrimination in the training and validation sets. This finding offers the potential for application in patients with renal failure or contrast allergies. In the CECT dataset, the predictive performance of the combined model (AUC: 0.836) and the clinical model (AUC: 0.851) was higher than that of the radscore model (AUC: 0.618; *p* = 0.002 and <0.001, respectively). This result could provide some hints that assessing the CECT qualitative and quantitative features such as UECT ratio, diameter, and enhancement homogeneity could reduce the unnecessary non-therapeutic thymectomy rate in AMMs. Comparing the UECT combined model (AUC: 0.870) to the CECT clinical model (AUC: 0.851), we see that their performance is very similar. In this case, expert-based lesion quantification on CECT is probably more efficient and faster than expert-based lesion quantification on UECT plus target delineation and radiomics calculation.

Several limitations of this pilot study must be acknowledged. First, this was a retrospective study in which only surgically resected AMMs were included, and patient selection bias was inevitable. Second, our study was subject to the inherent limitations of any single-center study, without external validation. Third, the technical parameters and scan protocols for the CT examinations were not consistent. Although resampling-based approaches have been adopted to address this problem, the potential influence of radiomic stability should still be considered. Another point of concern was that the CT imaging evaluations of both UECT and CECT groups were done based on the UECT scans, except for enhancement homogeneity and CECT ratio; therefore, we recommend that all patients require a UECT scan. In addition, the distribution of the pathological types of AMMs was uneven, and the number of TETs and cysts was relatively large. This might have affected the stability of the model. Finally, image processing algorithms and segmentation process techniques are bottlenecks of radiomics applications, which are time-consuming and highly prone to artificial error. This may restrict the further clinical application of these techniques, and there remains a need to develop a more reliable and robust segmentation tool.

## Conclusion

In patients who underwent UECT scan only, a nomogram model integrating the radscore and UECT ratio achieved good accuracy in predicting therapeutic thymectomy probability in AMMs. Nevertheless, the use of radiomics as a clinical biomarker in CECT scans did not improve the predictive performance of the model. A clinical model consisting of the diameter, UECT ratio, and homogeneity may be helpful and more practical in finding an appropriate solution for the management of AMMs. The individualized model may avoid blind follow-ups and high non-therapeutic thymectomy rates.

## Data Availability Statement

The raw data supporting the conclusions of this article will be made available by the authors, without undue reservation.

## Author Contributions

Conceptualization: YC and WH. Methodology: YC and YZ. Software: ZZ and BW. Validation: YZ, YC, and ZZ. Formal analysis: ZZ and BW. Investigation: YQ. Data curation: ZZ and YQ. Writing—original draft preparation: ZZ. Writing—review and editing: ZZ, YZ, and YC. Visualization: ZZ. Supervision: YC. Project administration: YC. Funding acquisition: YC. All authors have read and agreed to the published version of the manuscript.

## Funding

This work was supported by the Zhongnan Hospital of Wuhan University (znpy2019038).

## Conflict of Interest

The authors declare that the research was conducted in the absence of any commercial or financial relationships that could be construed as a potential conflict of interest.

## Publisher’s Note

All claims expressed in this article are solely those of the authors and do not necessarily represent those of their affiliated organizations, or those of the publisher, the editors and the reviewers. Any product that may be evaluated in this article, or claim that may be made by its manufacturer, is not guaranteed or endorsed by the publisher.
