# Supplementary material for: Development and Validation of a CT-Based Radiomics Nomogram in Patients With Anterior Mediastinal Mass: Individualized Options for Preoperative Patients
Source: Front Oncol. 2022 Jul 8;12:869253. doi: 10.3389/fonc.2022.869253 (PMC9304864; doi:10.3389/fonc.2022.869253)
Supplement: Supplementary file 1 [file DataSheet_1.docx]

Supplementary Material

**Content**

[**1. CT scan protocols 2**](#_Toc9380)

[**2. 1288 Radiomics features 2**](#_Toc9964)

[**3. Features (ICC>0.90) 12**](#_Toc25281)

[**4. UECT Features in LASSO 23**](#_Toc30057)

[**5. CECT features in LASSO 24**](#_Toc108)

[**6. Pyradiomics parameter 25**](#_Toc5662)

[**7. Radiomics reporting guidelines 27**](#_Toc29125)

#### **CT scan protocols**

|  | **Siemens Somatom Definition Flash** | **Philips Brilliance CT** | **Philips IQon spectral CT** | **GE Discovery CT750 HD** |
| --- | --- | --- | --- | --- |
| **Tube voltage(KV)** | 120 | 120 | 120 | 120 |
| **Tube current (mAs)** | adaptive tube current technology | adaptive tube current technology | adaptive tube current technology | adaptive tube current technology |
| **reconstruction thickness(mm)** | 1 | 1 | 1 | 1.25 |
| **reconstruction interval (mm)** | 1 | 1 | 1 | 1.25 |
| **Reconstruction algorithm** | Lung window setting | Lung window setting | Lung window setting | Lung window setting |
| **Aquisition matrix** | 512*512 | 512*512 | 512*512 | 512*512 |

#### **1288 Radiomics features**

original_shape_Elongation,original_shape_Flatness,original_shape_LeastAxisLength,original_shape_MajorAxisLength,original_shape_Maximum2DDiameterColumn,original_shape_Maximum2DDiameterRow,original_shape_Maximum2DDiameterSlice,original_shape_Maximum3DDiameter,original_shape_MeshVolume,original_shape_MinorAxisLength,original_shape_Sphericity,original_shape_SurfaceArea,original_shape_SurfaceVolumeRatio,original_shape_VoxelVolume,original_firstorder_10Percentile,original_firstorder_90Percentile,original_firstorder_Energy,original_firstorder_Entropy,original_firstorder_InterquartileRange,original_firstorder_Kurtosis,original_firstorder_Maximum,original_firstorder_MeanAbsoluteDeviation,original_firstorder_Mean,original_firstorder_Median,original_firstorder_Minimum,original_firstorder_Range,original_firstorder_RobustMeanAbsoluteDeviation,original_firstorder_RootMeanSquared,original_firstorder_Skewness,original_firstorder_TotalEnergy,original_firstorder_Uniformity,original_firstorder_Variance,original_glcm_Autocorrelation,original_glcm_JointAverage,original_glcm_ClusterProminence,original_glcm_ClusterShade,original_glcm_ClusterTendency,original_glcm_Contrast,original_glcm_Correlation,original_glcm_DifferenceAverage,original_glcm_DifferenceEntropy,original_glcm_DifferenceVariance,original_glcm_JointEnergy,original_glcm_JointEntropy,original_glcm_Imc1,original_glcm_Imc2,original_glcm_Idm,original_glcm_Idmn,original_glcm_Id,original_glcm_Idn,original_glcm_InverseVariance,original_glcm_MaximumProbability,original_glcm_SumEntropy,original_glcm_SumSquares,original_glrlm_GrayLevelNonUniformity,original_glrlm_GrayLevelNonUniformityNormalized,original_glrlm_GrayLevelVariance,original_glrlm_HighGrayLevelRunEmphasis,original_glrlm_LongRunEmphasis,original_glrlm_LongRunHighGrayLevelEmphasis,original_glrlm_LongRunLowGrayLevelEmphasis,original_glrlm_LowGrayLevelRunEmphasis,original_glrlm_RunEntropy,original_glrlm_RunLengthNonUniformity,original_glrlm_RunLengthNonUniformityNormalized,original_glrlm_RunPercentage,original_glrlm_RunVariance,original_glrlm_ShortRunEmphasis,original_glrlm_ShortRunHighGrayLevelEmphasis,original_glrlm_ShortRunLowGrayLevelEmphasis,original_glszm_GrayLevelNonUniformity,original_glszm_GrayLevelNonUniformityNormalized,original_glszm_GrayLevelVariance,original_glszm_HighGrayLevelZoneEmphasis,original_glszm_LargeAreaEmphasis,original_glszm_LargeAreaHighGrayLevelEmphasis,original_glszm_LargeAreaLowGrayLevelEmphasis,original_glszm_LowGrayLevelZoneEmphasis,original_glszm_SizeZoneNonUniformity,original_glszm_SizeZoneNonUniformityNormalized,original_glszm_SmallAreaEmphasis,original_glszm_SmallAreaHighGrayLevelEmphasis,original_glszm_SmallAreaLowGrayLevelEmphasis,original_glszm_ZoneEntropy,original_glszm_ZonePercentage,original_glszm_ZoneVariance,original_gldm_DependenceEntropy,original_gldm_DependenceNonUniformity,original_gldm_DependenceNonUniformityNormalized,original_gldm_DependenceVariance,original_gldm_GrayLevelNonUniformity,original_gldm_GrayLevelVariance,original_gldm_HighGrayLevelEmphasis,original_gldm_LargeDependenceEmphasis,original_gldm_LargeDependenceHighGrayLevelEmphasis,original_gldm_LargeDependenceLowGrayLevelEmphasis,original_gldm_LowGrayLevelEmphasis,original_gldm_SmallDependenceEmphasis,original_gldm_SmallDependenceHighGrayLevelEmphasis,original_gldm_SmallDependenceLowGrayLevelEmphasis,original_ngtdm_Busyness,original_ngtdm_Coarseness,original_ngtdm_Complexity,original_ngtdm_Contrast,original_ngtdm_Strength,log-sigma-1-0-mm-3D_firstorder_10Percentile,log-sigma-1-0-mm-3D_firstorder_90Percentile,log-sigma-1-0-mm-3D_firstorder_Energy,log-sigma-1-0-mm-3D_firstorder_Entropy,log-sigma-1-0-mm-3D_firstorder_InterquartileRange,log-sigma-1-0-mm-3D_firstorder_Kurtosis,log-sigma-1-0-mm-3D_firstorder_Maximum,log-sigma-1-0-mm-3D_firstorder_MeanAbsoluteDeviation,log-sigma-1-0-mm-3D_firstorder_Mean,log-sigma-1-0-mm-3D_firstorder_Median,log-sigma-1-0-mm-3D_firstorder_Minimum,log-sigma-1-0-mm-3D_firstorder_Range,log-sigma-1-0-mm-3D_firstorder_RobustMeanAbsoluteDeviation,log-sigma-1-0-mm-3D_firstorder_RootMeanSquared,log-sigma-1-0-mm-3D_firstorder_Skewness,log-sigma-1-0-mm-3D_firstorder_TotalEnergy,log-sigma-1-0-mm-3D_firstorder_Uniformity,log-sigma-1-0-mm-3D_firstorder_Variance,log-sigma-1-0-mm-3D_glcm_Autocorrelation,log-sigma-1-0-mm-3D_glcm_JointAverage,log-sigma-1-0-mm-3D_glcm_ClusterProminence,log-sigma-1-0-mm-3D_glcm_ClusterShade,log-sigma-1-0-mm-3D_glcm_ClusterTendency,log-sigma-1-0-mm-3D_glcm_Contrast,log-sigma-1-0-mm-3D_glcm_Correlation,log-sigma-1-0-mm-3D_glcm_DifferenceAverage,log-sigma-1-0-mm-3D_glcm_DifferenceEntropy,log-sigma-1-0-mm-3D_glcm_DifferenceVariance,log-sigma-1-0-mm-3D_glcm_JointEnergy,log-sigma-1-0-mm-3D_glcm_JointEntropy,log-sigma-1-0-mm-3D_glcm_Imc1,log-sigma-1-0-mm-3D_glcm_Imc2,log-sigma-1-0-mm-3D_glcm_Idm,log-sigma-1-0-mm-3D_glcm_Idmn,log-sigma-1-0-mm-3D_glcm_Id,log-sigma-1-0-mm-3D_glcm_Idn,log-sigma-1-0-mm-3D_glcm_InverseVariance,log-sigma-1-0-mm-3D_glcm_MaximumProbability,log-sigma-1-0-mm-3D_glcm_SumEntropy,log-sigma-1-0-mm-3D_glcm_SumSquares,log-sigma-1-0-mm-3D_glrlm_GrayLevelNonUniformity,log-sigma-1-0-mm-3D_glrlm_GrayLevelNonUniformityNormalized,log-sigma-1-0-mm-3D_glrlm_GrayLevelVariance,log-sigma-1-0-mm-3D_glrlm_HighGrayLevelRunEmphasis,log-sigma-1-0-mm-3D_glrlm_LongRunEmphasis,log-sigma-1-0-mm-3D_glrlm_LongRunHighGrayLevelEmphasis,log-sigma-1-0-mm-3D_glrlm_LongRunLowGrayLevelEmphasis,log-sigma-1-0-mm-3D_glrlm_LowGrayLevelRunEmphasis,log-sigma-1-0-mm-3D_glrlm_RunEntropy,log-sigma-1-0-mm-3D_glrlm_RunLengthNonUniformity,log-sigma-1-0-mm-3D_glrlm_RunLengthNonUniformityNormalized,log-sigma-1-0-mm-3D_glrlm_RunPercentage,log-sigma-1-0-mm-3D_glrlm_RunVariance,log-sigma-1-0-mm-3D_glrlm_ShortRunEmphasis,log-sigma-1-0-mm-3D_glrlm_ShortRunHighGrayLevelEmphasis,log-sigma-1-0-mm-3D_glrlm_ShortRunLowGrayLevelEmphasis,log-sigma-1-0-mm-3D_glszm_GrayLevelNonUniformity,log-sigma-1-0-mm-3D_glszm_GrayLevelNonUniformityNormalized,log-sigma-1-0-mm-3D_glszm_GrayLevelVariance,log-sigma-1-0-mm-3D_glszm_HighGrayLevelZoneEmphasis,log-sigma-1-0-mm-3D_glszm_LargeAreaEmphasis,log-sigma-1-0-mm-3D_glszm_LargeAreaHighGrayLevelEmphasis,log-sigma-1-0-mm-3D_glszm_LargeAreaLowGrayLevelEmphasis,log-sigma-1-0-mm-3D_glszm_LowGrayLevelZoneEmphasis,log-sigma-1-0-mm-3D_glszm_SizeZoneNonUniformity,log-sigma-1-0-mm-3D_glszm_SizeZoneNonUniformityNormalized,log-sigma-1-0-mm-3D_glszm_SmallAreaEmphasis,log-sigma-1-0-mm-3D_glszm_SmallAreaHighGrayLevelEmphasis,log-sigma-1-0-mm-3D_glszm_SmallAreaLowGrayLevelEmphasis,log-sigma-1-0-mm-3D_glszm_ZoneEntropy,log-sigma-1-0-mm-3D_glszm_ZonePercentage,log-sigma-1-0-mm-3D_glszm_ZoneVariance,log-sigma-1-0-mm-3D_gldm_DependenceEntropy,log-sigma-1-0-mm-3D_gldm_DependenceNonUniformity,log-sigma-1-0-mm-3D_gldm_DependenceNonUniformityNormalized,log-sigma-1-0-mm-3D_gldm_DependenceVariance,log-sigma-1-0-mm-3D_gldm_GrayLevelNonUniformity,log-sigma-1-0-mm-3D_gldm_GrayLevelVariance,log-sigma-1-0-mm-3D_gldm_HighGrayLevelEmphasis,log-sigma-1-0-mm-3D_gldm_LargeDependenceEmphasis,log-sigma-1-0-mm-3D_gldm_LargeDependenceHighGrayLevelEmphasis,log-sigma-1-0-mm-3D_gldm_LargeDependenceLowGrayLevelEmphasis,log-sigma-1-0-mm-3D_gldm_LowGrayLevelEmphasis,log-sigma-1-0-mm-3D_gldm_SmallDependenceEmphasis,log-sigma-1-0-mm-3D_gldm_SmallDependenceHighGrayLevelEmphasis,log-sigma-1-0-mm-3D_gldm_SmallDependenceLowGrayLevelEmphasis,log-sigma-1-0-mm-3D_ngtdm_Busyness,log-sigma-1-0-mm-3D_ngtdm_Coarseness,log-sigma-1-0-mm-3D_ngtdm_Complexity,log-sigma-1-0-mm-3D_ngtdm_Contrast,log-sigma-1-0-mm-3D_ngtdm_Strength,log-sigma-2-0-mm-3D_firstorder_10Percentile,log-sigma-2-0-mm-3D_firstorder_90Percentile,log-sigma-2-0-mm-3D_firstorder_Energy,log-sigma-2-0-mm-3D_firstorder_Entropy,log-sigma-2-0-mm-3D_firstorder_InterquartileRange,log-sigma-2-0-mm-3D_firstorder_Kurtosis,log-sigma-2-0-mm-3D_firstorder_Maximum,log-sigma-2-0-mm-3D_firstorder_MeanAbsoluteDeviation,log-sigma-2-0-mm-3D_firstorder_Mean,log-sigma-2-0-mm-3D_firstorder_Median,log-sigma-2-0-mm-3D_firstorder_Minimum,log-sigma-2-0-mm-3D_firstorder_Range,log-sigma-2-0-mm-3D_firstorder_RobustMeanAbsoluteDeviation,log-sigma-2-0-mm-3D_firstorder_RootMeanSquared,log-sigma-2-0-mm-3D_firstorder_Skewness,log-sigma-2-0-mm-3D_firstorder_TotalEnergy,log-sigma-2-0-mm-3D_firstorder_Uniformity,log-sigma-2-0-mm-3D_firstorder_Variance,log-sigma-2-0-mm-3D_glcm_Autocorrelation,log-sigma-2-0-mm-3D_glcm_JointAverage,log-sigma-2-0-mm-3D_glcm_ClusterProminence,log-sigma-2-0-mm-3D_glcm_ClusterShade,log-sigma-2-0-mm-3D_glcm_ClusterTendency,log-sigma-2-0-mm-3D_glcm_Contrast,log-sigma-2-0-mm-3D_glcm_Correlation,log-sigma-2-0-mm-3D_glcm_DifferenceAverage,log-sigma-2-0-mm-3D_glcm_DifferenceEntropy,log-sigma-2-0-mm-3D_glcm_DifferenceVariance,log-sigma-2-0-mm-3D_glcm_JointEnergy,log-sigma-2-0-mm-3D_glcm_JointEntropy,log-sigma-2-0-mm-3D_glcm_Imc1,log-sigma-2-0-mm-3D_glcm_Imc2,log-sigma-2-0-mm-3D_glcm_Idm,log-sigma-2-0-mm-3D_glcm_Idmn,log-sigma-2-0-mm-3D_glcm_Id,log-sigma-2-0-mm-3D_glcm_Idn,log-sigma-2-0-mm-3D_glcm_InverseVariance,log-sigma-2-0-mm-3D_glcm_MaximumProbability,log-sigma-2-0-mm-3D_glcm_SumEntropy,log-sigma-2-0-mm-3D_glcm_SumSquares,log-sigma-2-0-mm-3D_glrlm_GrayLevelNonUniformity,log-sigma-2-0-mm-3D_glrlm_GrayLevelNonUniformityNormalized,log-sigma-2-0-mm-3D_glrlm_GrayLevelVariance,log-sigma-2-0-mm-3D_glrlm_HighGrayLevelRunEmphasis,log-sigma-2-0-mm-3D_glrlm_LongRunEmphasis,log-sigma-2-0-mm-3D_glrlm_LongRunHighGrayLevelEmphasis,log-sigma-2-0-mm-3D_glrlm_LongRunLowGrayLevelEmphasis,log-sigma-2-0-mm-3D_glrlm_LowGrayLevelRunEmphasis,log-sigma-2-0-mm-3D_glrlm_RunEntropy,log-sigma-2-0-mm-3D_glrlm_RunLengthNonUniformity,log-sigma-2-0-mm-3D_glrlm_RunLengthNonUniformityNormalized,log-sigma-2-0-mm-3D_glrlm_RunPercentage,log-sigma-2-0-mm-3D_glrlm_RunVariance,log-sigma-2-0-mm-3D_glrlm_ShortRunEmphasis,log-sigma-2-0-mm-3D_glrlm_ShortRunHighGrayLevelEmphasis,log-sigma-2-0-mm-3D_glrlm_ShortRunLowGrayLevelEmphasis,log-sigma-2-0-mm-3D_glszm_GrayLevelNonUniformity,log-sigma-2-0-mm-3D_glszm_GrayLevelNonUniformityNormalized,log-sigma-2-0-mm-3D_glszm_GrayLevelVariance,log-sigma-2-0-mm-3D_glszm_HighGrayLevelZoneEmphasis,log-sigma-2-0-mm-3D_glszm_LargeAreaEmphasis,log-sigma-2-0-mm-3D_glszm_LargeAreaHighGrayLevelEmphasis,log-sigma-2-0-mm-3D_glszm_LargeAreaLowGrayLevelEmphasis,log-sigma-2-0-mm-3D_glszm_LowGrayLevelZoneEmphasis,log-sigma-2-0-mm-3D_glszm_SizeZoneNonUniformity,log-sigma-2-0-mm-3D_glszm_SizeZoneNonUniformityNormalized,log-sigma-2-0-mm-3D_glszm_SmallAreaEmphasis,log-sigma-2-0-mm-3D_glszm_SmallAreaHighGrayLevelEmphasis,log-sigma-2-0-mm-3D_glszm_SmallAreaLowGrayLevelEmphasis,log-sigma-2-0-mm-3D_glszm_ZoneEntropy,log-sigma-2-0-mm-3D_glszm_ZonePercentage,log-sigma-2-0-mm-3D_glszm_ZoneVariance,log-sigma-2-0-mm-3D_gldm_DependenceEntropy,log-sigma-2-0-mm-3D_gldm_DependenceNonUniformity,log-sigma-2-0-mm-3D_gldm_DependenceNonUniformityNormalized,log-sigma-2-0-mm-3D_gldm_DependenceVariance,log-sigma-2-0-mm-3D_gldm_GrayLevelNonUniformity,log-sigma-2-0-mm-3D_gldm_GrayLevelVariance,log-sigma-2-0-mm-3D_gldm_HighGrayLevelEmphasis,log-sigma-2-0-mm-3D_gldm_LargeDependenceEmphasis,log-sigma-2-0-mm-3D_gldm_LargeDependenceHighGrayLevelEmphasis,log-sigma-2-0-mm-3D_gldm_LargeDependenceLowGrayLevelEmphasis,log-sigma-2-0-mm-3D_gldm_LowGrayLevelEmphasis,log-sigma-2-0-mm-3D_gldm_SmallDependenceEmphasis,log-sigma-2-0-mm-3D_gldm_SmallDependenceHighGrayLevelEmphasis,log-sigma-2-0-mm-3D_gldm_SmallDependenceLowGrayLevelEmphasis,log-sigma-2-0-mm-3D_ngtdm_Busyness,log-sigma-2-0-mm-3D_ngtdm_Coarseness,log-sigma-2-0-mm-3D_ngtdm_Complexity,log-sigma-2-0-mm-3D_ngtdm_Contrast,log-sigma-2-0-mm-3D_ngtdm_Strength,log-sigma-3-0-mm-3D_firstorder_10Percentile,log-sigma-3-0-mm-3D_firstorder_90Percentile,log-sigma-3-0-mm-3D_firstorder_Energy,log-sigma-3-0-mm-3D_firstorder_Entropy,log-sigma-3-0-mm-3D_firstorder_InterquartileRange,log-sigma-3-0-mm-3D_firstorder_Kurtosis,log-sigma-3-0-mm-3D_firstorder_Maximum,log-sigma-3-0-mm-3D_firstorder_MeanAbsoluteDeviation,log-sigma-3-0-mm-3D_firstorder_Mean,log-sigma-3-0-mm-3D_firstorder_Median,log-sigma-3-0-mm-3D_firstorder_Minimum,log-sigma-3-0-mm-3D_firstorder_Range,log-sigma-3-0-mm-3D_firstorder_RobustMeanAbsoluteDeviation,log-sigma-3-0-mm-3D_firstorder_RootMeanSquared,log-sigma-3-0-mm-3D_firstorder_Skewness,log-sigma-3-0-mm-3D_firstorder_TotalEnergy,log-sigma-3-0-mm-3D_firstorder_Uniformity,log-sigma-3-0-mm-3D_firstorder_Variance,log-sigma-3-0-mm-3D_glcm_Autocorrelation,log-sigma-3-0-mm-3D_glcm_JointAverage,log-sigma-3-0-mm-3D_glcm_ClusterProminence,log-sigma-3-0-mm-3D_glcm_ClusterShade,log-sigma-3-0-mm-3D_glcm_ClusterTendency,log-sigma-3-0-mm-3D_glcm_Contrast,log-sigma-3-0-mm-3D_glcm_Correlation,log-sigma-3-0-mm-3D_glcm_DifferenceAverage,log-sigma-3-0-mm-3D_glcm_DifferenceEntropy,log-sigma-3-0-mm-3D_glcm_DifferenceVariance,log-sigma-3-0-mm-3D_glcm_JointEnergy,log-sigma-3-0-mm-3D_glcm_JointEntropy,log-sigma-3-0-mm-3D_glcm_Imc1,log-sigma-3-0-mm-3D_glcm_Imc2,log-sigma-3-0-mm-3D_glcm_Idm,log-sigma-3-0-mm-3D_glcm_Idmn,log-sigma-3-0-mm-3D_glcm_Id,log-sigma-3-0-mm-3D_glcm_Idn,log-sigma-3-0-mm-3D_glcm_InverseVariance,log-sigma-3-0-mm-3D_glcm_MaximumProbability,log-sigma-3-0-mm-3D_glcm_SumEntropy,log-sigma-3-0-mm-3D_glcm_SumSquares,log-sigma-3-0-mm-3D_glrlm_GrayLevelNonUniformity,log-sigma-3-0-mm-3D_glrlm_GrayLevelNonUniformityNormalized,log-sigma-3-0-mm-3D_glrlm_GrayLevelVariance,log-sigma-3-0-mm-3D_glrlm_HighGrayLevelRunEmphasis,log-sigma-3-0-mm-3D_glrlm_LongRunEmphasis,log-sigma-3-0-mm-3D_glrlm_LongRunHighGrayLevelEmphasis,log-sigma-3-0-mm-3D_glrlm_LongRunLowGrayLevelEmphasis,log-sigma-3-0-mm-3D_glrlm_LowGrayLevelRunEmphasis,log-sigma-3-0-mm-3D_glrlm_RunEntropy,log-sigma-3-0-mm-3D_glrlm_RunLengthNonUniformity,log-sigma-3-0-mm-3D_glrlm_RunLengthNonUniformityNormalized,log-sigma-3-0-mm-3D_glrlm_RunPercentage,log-sigma-3-0-mm-3D_glrlm_RunVariance,log-sigma-3-0-mm-3D_glrlm_ShortRunEmphasis,log-sigma-3-0-mm-3D_glrlm_ShortRunHighGrayLevelEmphasis,log-sigma-3-0-mm-3D_glrlm_ShortRunLowGrayLevelEmphasis,log-sigma-3-0-mm-3D_glszm_GrayLevelNonUniformity,log-sigma-3-0-mm-3D_glszm_GrayLevelNonUniformityNormalized,log-sigma-3-0-mm-3D_glszm_GrayLevelVariance,log-sigma-3-0-mm-3D_glszm_HighGrayLevelZoneEmphasis,log-sigma-3-0-mm-3D_glszm_LargeAreaEmphasis,log-sigma-3-0-mm-3D_glszm_LargeAreaHighGrayLevelEmphasis,log-sigma-3-0-mm-3D_glszm_LargeAreaLowGrayLevelEmphasis,log-sigma-3-0-mm-3D_glszm_LowGrayLevelZoneEmphasis,log-sigma-3-0-mm-3D_glszm_SizeZoneNonUniformity,log-sigma-3-0-mm-3D_glszm_SizeZoneNonUniformityNormalized,log-sigma-3-0-mm-3D_glszm_SmallAreaEmphasis,log-sigma-3-0-mm-3D_glszm_SmallAreaHighGrayLevelEmphasis,log-sigma-3-0-mm-3D_glszm_SmallAreaLowGrayLevelEmphasis,log-sigma-3-0-mm-3D_glszm_ZoneEntropy,log-sigma-3-0-mm-3D_glszm_ZonePercentage,log-sigma-3-0-mm-3D_glszm_ZoneVariance,log-sigma-3-0-mm-3D_gldm_DependenceEntropy,log-sigma-3-0-mm-3D_gldm_DependenceNonUniformity,log-sigma-3-0-mm-3D_gldm_DependenceNonUniformityNormalized,log-sigma-3-0-mm-3D_gldm_DependenceVariance,log-sigma-3-0-mm-3D_gldm_GrayLevelNonUniformity,log-sigma-3-0-mm-3D_gldm_GrayLevelVariance,log-sigma-3-0-mm-3D_gldm_HighGrayLevelEmphasis,log-sigma-3-0-mm-3D_gldm_LargeDependenceEmphasis,log-sigma-3-0-mm-3D_gldm_LargeDependenceHighGrayLevelEmphasis,log-sigma-3-0-mm-3D_gldm_LargeDependenceLowGrayLevelEmphasis,log-sigma-3-0-mm-3D_gldm_LowGrayLevelEmphasis,log-sigma-3-0-mm-3D_gldm_SmallDependenceEmphasis,log-sigma-3-0-mm-3D_gldm_SmallDependenceHighGrayLevelEmphasis,log-sigma-3-0-mm-3D_gldm_SmallDependenceLowGrayLevelEmphasis,log-sigma-3-0-mm-3D_ngtdm_Busyness,log-sigma-3-0-mm-3D_ngtdm_Coarseness,log-sigma-3-0-mm-3D_ngtdm_Complexity,log-sigma-3-0-mm-3D_ngtdm_Contrast,log-sigma-3-0-mm-3D_ngtdm_Strength,log-sigma-4-0-mm-3D_firstorder_10Percentile,log-sigma-4-0-mm-3D_firstorder_90Percentile,log-sigma-4-0-mm-3D_firstorder_Energy,log-sigma-4-0-mm-3D_firstorder_Entropy,log-sigma-4-0-mm-3D_firstorder_InterquartileRange,log-sigma-4-0-mm-3D_firstorder_Kurtosis,log-sigma-4-0-mm-3D_firstorder_Maximum,log-sigma-4-0-mm-3D_firstorder_MeanAbsoluteDeviation,log-sigma-4-0-mm-3D_firstorder_Mean,log-sigma-4-0-mm-3D_firstorder_Median,log-sigma-4-0-mm-3D_firstorder_Minimum,log-sigma-4-0-mm-3D_firstorder_Range,log-sigma-4-0-mm-3D_firstorder_RobustMeanAbsoluteDeviation,log-sigma-4-0-mm-3D_firstorder_RootMeanSquared,log-sigma-4-0-mm-3D_firstorder_Skewness,log-sigma-4-0-mm-3D_firstorder_TotalEnergy,log-sigma-4-0-mm-3D_firstorder_Uniformity,log-sigma-4-0-mm-3D_firstorder_Variance,log-sigma-4-0-mm-3D_glcm_Autocorrelation,log-sigma-4-0-mm-3D_glcm_JointAverage,log-sigma-4-0-mm-3D_glcm_ClusterProminence,log-sigma-4-0-mm-3D_glcm_ClusterShade,log-sigma-4-0-mm-3D_glcm_ClusterTendency,log-sigma-4-0-mm-3D_glcm_Contrast,log-sigma-4-0-mm-3D_glcm_Correlation,log-sigma-4-0-mm-3D_glcm_DifferenceAverage,log-sigma-4-0-mm-3D_glcm_DifferenceEntropy,log-sigma-4-0-mm-3D_glcm_DifferenceVariance,log-sigma-4-0-mm-3D_glcm_JointEnergy,log-sigma-4-0-mm-3D_glcm_JointEntropy,log-sigma-4-0-mm-3D_glcm_Imc1,log-sigma-4-0-mm-3D_glcm_Imc2,log-sigma-4-0-mm-3D_glcm_Idm,log-sigma-4-0-mm-3D_glcm_Idmn,log-sigma-4-0-mm-3D_glcm_Id,log-sigma-4-0-mm-3D_glcm_Idn,log-sigma-4-0-mm-3D_glcm_InverseVariance,log-sigma-4-0-mm-3D_glcm_MaximumProbability,log-sigma-4-0-mm-3D_glcm_SumEntropy,log-sigma-4-0-mm-3D_glcm_SumSquares,log-sigma-4-0-mm-3D_glrlm_GrayLevelNonUniformity,log-sigma-4-0-mm-3D_glrlm_GrayLevelNonUniformityNormalized,log-sigma-4-0-mm-3D_glrlm_GrayLevelVariance,log-sigma-4-0-mm-3D_glrlm_HighGrayLevelRunEmphasis,log-sigma-4-0-mm-3D_glrlm_LongRunEmphasis,log-sigma-4-0-mm-3D_glrlm_LongRunHighGrayLevelEmphasis,log-sigma-4-0-mm-3D_glrlm_LongRunLowGrayLevelEmphasis,log-sigma-4-0-mm-3D_glrlm_LowGrayLevelRunEmphasis,log-sigma-4-0-mm-3D_glrlm_RunEntropy,log-sigma-4-0-mm-3D_glrlm_RunLengthNonUniformity,log-sigma-4-0-mm-3D_glrlm_RunLengthNonUniformityNormalized,log-sigma-4-0-mm-3D_glrlm_RunPercentage,log-sigma-4-0-mm-3D_glrlm_RunVariance,log-sigma-4-0-mm-3D_glrlm_ShortRunEmphasis,log-sigma-4-0-mm-3D_glrlm_ShortRunHighGrayLevelEmphasis,log-sigma-4-0-mm-3D_glrlm_ShortRunLowGrayLevelEmphasis,log-sigma-4-0-mm-3D_glszm_GrayLevelNonUniformity,log-sigma-4-0-mm-3D_glszm_GrayLevelNonUniformityNormalized,log-sigma-4-0-mm-3D_glszm_GrayLevelVariance,log-sigma-4-0-mm-3D_glszm_HighGrayLevelZoneEmphasis,log-sigma-4-0-mm-3D_glszm_LargeAreaEmphasis,log-sigma-4-0-mm-3D_glszm_LargeAreaHighGrayLevelEmphasis,log-sigma-4-0-mm-3D_glszm_LargeAreaLowGrayLevelEmphasis,log-sigma-4-0-mm-3D_glszm_LowGrayLevelZoneEmphasis,log-sigma-4-0-mm-3D_glszm_SizeZoneNonUniformity,log-sigma-4-0-mm-3D_glszm_SizeZoneNonUniformityNormalized,log-sigma-4-0-mm-3D_glszm_SmallAreaEmphasis,log-sigma-4-0-mm-3D_glszm_SmallAreaHighGrayLevelEmphasis,log-sigma-4-0-mm-3D_glszm_SmallAreaLowGrayLevelEmphasis,log-sigma-4-0-mm-3D_glszm_ZoneEntropy,log-sigma-4-0-mm-3D_glszm_ZonePercentage,log-sigma-4-0-mm-3D_glszm_ZoneVariance,log-sigma-4-0-mm-3D_gldm_DependenceEntropy,log-sigma-4-0-mm-3D_gldm_DependenceNonUniformity,log-sigma-4-0-mm-3D_gldm_DependenceNonUniformityNormalized,log-sigma-4-0-mm-3D_gldm_DependenceVariance,log-sigma-4-0-mm-3D_gldm_GrayLevelNonUniformity,log-sigma-4-0-mm-3D_gldm_GrayLevelVariance,log-sigma-4-0-mm-3D_gldm_HighGrayLevelEmphasis,log-sigma-4-0-mm-3D_gldm_LargeDependenceEmphasis,log-sigma-4-0-mm-3D_gldm_LargeDependenceHighGrayLevelEmphasis,log-sigma-4-0-mm-3D_gldm_LargeDependenceLowGrayLevelEmphasis,log-sigma-4-0-mm-3D_gldm_LowGrayLevelEmphasis,log-sigma-4-0-mm-3D_gldm_SmallDependenceEmphasis,log-sigma-4-0-mm-3D_gldm_SmallDependenceHighGrayLevelEmphasis,log-sigma-4-0-mm-3D_gldm_SmallDependenceLowGrayLevelEmphasis,log-sigma-4-0-mm-3D_ngtdm_Busyness,log-sigma-4-0-mm-3D_ngtdm_Coarseness,log-sigma-4-0-mm-3D_ngtdm_Complexity,log-sigma-4-0-mm-3D_ngtdm_Contrast,log-sigma-4-0-mm-3D_ngtdm_Strength,log-sigma-5-0-mm-3D_firstorder_10Percentile,log-sigma-5-0-mm-3D_firstorder_90Percentile,log-sigma-5-0-mm-3D_firstorder_Energy,log-sigma-5-0-mm-3D_firstorder_Entropy,log-sigma-5-0-mm-3D_firstorder_InterquartileRange,log-sigma-5-0-mm-3D_firstorder_Kurtosis,log-sigma-5-0-mm-3D_firstorder_Maximum,log-sigma-5-0-mm-3D_firstorder_MeanAbsoluteDeviation,log-sigma-5-0-mm-3D_firstorder_Mean,log-sigma-5-0-mm-3D_firstorder_Median,log-sigma-5-0-mm-3D_firstorder_Minimum,log-sigma-5-0-mm-3D_firstorder_Range,log-sigma-5-0-mm-3D_firstorder_RobustMeanAbsoluteDeviation,log-sigma-5-0-mm-3D_firstorder_RootMeanSquared,log-sigma-5-0-mm-3D_firstorder_Skewness,log-sigma-5-0-mm-3D_firstorder_TotalEnergy,log-sigma-5-0-mm-3D_firstorder_Uniformity,log-sigma-5-0-mm-3D_firstorder_Variance,log-sigma-5-0-mm-3D_glcm_Autocorrelation,log-sigma-5-0-mm-3D_glcm_JointAverage,log-sigma-5-0-mm-3D_glcm_ClusterProminence,log-sigma-5-0-mm-3D_glcm_ClusterShade,log-sigma-5-0-mm-3D_glcm_ClusterTendency,log-sigma-5-0-mm-3D_glcm_Contrast,log-sigma-5-0-mm-3D_glcm_Correlation,log-sigma-5-0-mm-3D_glcm_DifferenceAverage,log-sigma-5-0-mm-3D_glcm_DifferenceEntropy,log-sigma-5-0-mm-3D_glcm_DifferenceVariance,log-sigma-5-0-mm-3D_glcm_JointEnergy,log-sigma-5-0-mm-3D_glcm_JointEntropy,log-sigma-5-0-mm-3D_glcm_Imc1,log-sigma-5-0-mm-3D_glcm_Imc2,log-sigma-5-0-mm-3D_glcm_Idm,log-sigma-5-0-mm-3D_glcm_Idmn,log-sigma-5-0-mm-3D_glcm_Id,log-sigma-5-0-mm-3D_glcm_Idn,log-sigma-5-0-mm-3D_glcm_InverseVariance,log-sigma-5-0-mm-3D_glcm_MaximumProbability,log-sigma-5-0-mm-3D_glcm_SumEntropy,log-sigma-5-0-mm-3D_glcm_SumSquares,log-sigma-5-0-mm-3D_glrlm_GrayLevelNonUniformity,log-sigma-5-0-mm-3D_glrlm_GrayLevelNonUniformityNormalized,log-sigma-5-0-mm-3D_glrlm_GrayLevelVariance,log-sigma-5-0-mm-3D_glrlm_HighGrayLevelRunEmphasis,log-sigma-5-0-mm-3D_glrlm_LongRunEmphasis,log-sigma-5-0-mm-3D_glrlm_LongRunHighGrayLevelEmphasis,log-sigma-5-0-mm-3D_glrlm_LongRunLowGrayLevelEmphasis,log-sigma-5-0-mm-3D_glrlm_LowGrayLevelRunEmphasis,log-sigma-5-0-mm-3D_glrlm_RunEntropy,log-sigma-5-0-mm-3D_glrlm_RunLengthNonUniformity,log-sigma-5-0-mm-3D_glrlm_RunLengthNonUniformityNormalized,log-sigma-5-0-mm-3D_glrlm_RunPercentage,log-sigma-5-0-mm-3D_glrlm_RunVariance,log-sigma-5-0-mm-3D_glrlm_ShortRunEmphasis,log-sigma-5-0-mm-3D_glrlm_ShortRunHighGrayLevelEmphasis,log-sigma-5-0-mm-3D_glrlm_ShortRunLowGrayLevelEmphasis,log-sigma-5-0-mm-3D_glszm_GrayLevelNonUniformity,log-sigma-5-0-mm-3D_glszm_GrayLevelNonUniformityNormalized,log-sigma-5-0-mm-3D_glszm_GrayLevelVariance,log-sigma-5-0-mm-3D_glszm_HighGrayLevelZoneEmphasis,log-sigma-5-0-mm-3D_glszm_LargeAreaEmphasis,log-sigma-5-0-mm-3D_glszm_LargeAreaHighGrayLevelEmphasis,log-sigma-5-0-mm-3D_glszm_LargeAreaLowGrayLevelEmphasis,log-sigma-5-0-mm-3D_glszm_LowGrayLevelZoneEmphasis,log-sigma-5-0-mm-3D_glszm_SizeZoneNonUniformity,log-sigma-5-0-mm-3D_glszm_SizeZoneNonUniformityNormalized,log-sigma-5-0-mm-3D_glszm_SmallAreaEmphasis,log-sigma-5-0-mm-3D_glszm_SmallAreaHighGrayLevelEmphasis,log-sigma-5-0-mm-3D_glszm_SmallAreaLowGrayLevelEmphasis,log-sigma-5-0-mm-3D_glszm_ZoneEntropy,log-sigma-5-0-mm-3D_glszm_ZonePercentage,log-sigma-5-0-mm-3D_glszm_ZoneVariance,log-sigma-5-0-mm-3D_gldm_DependenceEntropy,log-sigma-5-0-mm-3D_gldm_DependenceNonUniformity,log-sigma-5-0-mm-3D_gldm_DependenceNonUniformityNormalized,log-sigma-5-0-mm-3D_gldm_DependenceVariance,log-sigma-5-0-mm-3D_gldm_GrayLevelNonUniformity,log-sigma-5-0-mm-3D_gldm_GrayLevelVariance,log-sigma-5-0-mm-3D_gldm_HighGrayLevelEmphasis,log-sigma-5-0-mm-3D_gldm_LargeDependenceEmphasis,log-sigma-5-0-mm-3D_gldm_LargeDependenceHighGrayLevelEmphasis,log-sigma-5-0-mm-3D_gldm_LargeDependenceLowGrayLevelEmphasis,log-sigma-5-0-mm-3D_gldm_LowGrayLevelEmphasis,log-sigma-5-0-mm-3D_gldm_SmallDependenceEmphasis,log-sigma-5-0-mm-3D_gldm_SmallDependenceHighGrayLevelEmphasis,log-sigma-5-0-mm-3D_gldm_SmallDependenceLowGrayLevelEmphasis,log-sigma-5-0-mm-3D_ngtdm_Busyness,log-sigma-5-0-mm-3D_ngtdm_Coarseness,log-sigma-5-0-mm-3D_ngtdm_Complexity,log-sigma-5-0-mm-3D_ngtdm_Contrast,log-sigma-5-0-mm-3D_ngtdm_Strength,wavelet-LLH_firstorder_10Percentile,wavelet-LLH_firstorder_90Percentile,wavelet-LLH_firstorder_Energy,wavelet-LLH_firstorder_Entropy,wavelet-LLH_firstorder_InterquartileRange,wavelet-LLH_firstorder_Kurtosis,wavelet-LLH_firstorder_Maximum,wavelet-LLH_firstorder_MeanAbsoluteDeviation,wavelet-LLH_firstorder_Mean,wavelet-LLH_firstorder_Median,wavelet-LLH_firstorder_Minimum,wavelet-LLH_firstorder_Range,wavelet-LLH_firstorder_RobustMeanAbsoluteDeviation,wavelet-LLH_firstorder_RootMeanSquared,wavelet-LLH_firstorder_Skewness,wavelet-LLH_firstorder_TotalEnergy,wavelet-LLH_firstorder_Uniformity,wavelet-LLH_firstorder_Variance,wavelet-LLH_glcm_Autocorrelation,wavelet-LLH_glcm_JointAverage,wavelet-LLH_glcm_ClusterProminence,wavelet-LLH_glcm_ClusterShade,wavelet-LLH_glcm_ClusterTendency,wavelet-LLH_glcm_Contrast,wavelet-LLH_glcm_Correlation,wavelet-LLH_glcm_DifferenceAverage,wavelet-LLH_glcm_DifferenceEntropy,wavelet-LLH_glcm_DifferenceVariance,wavelet-LLH_glcm_JointEnergy,wavelet-LLH_glcm_JointEntropy,wavelet-LLH_glcm_Imc1,wavelet-LLH_glcm_Imc2,wavelet-LLH_glcm_Idm,wavelet-LLH_glcm_Idmn,wavelet-LLH_glcm_Id,wavelet-LLH_glcm_Idn,wavelet-LLH_glcm_InverseVariance,wavelet-LLH_glcm_MaximumProbability,wavelet-LLH_glcm_SumEntropy,wavelet-LLH_glcm_SumSquares,wavelet-LLH_glrlm_GrayLevelNonUniformity,wavelet-LLH_glrlm_GrayLevelNonUniformityNormalized,wavelet-LLH_glrlm_GrayLevelVariance,wavelet-LLH_glrlm_HighGrayLevelRunEmphasis,wavelet-LLH_glrlm_LongRunEmphasis,wavelet-LLH_glrlm_LongRunHighGrayLevelEmphasis,wavelet-LLH_glrlm_LongRunLowGrayLevelEmphasis,wavelet-LLH_glrlm_LowGrayLevelRunEmphasis,wavelet-LLH_glrlm_RunEntropy,wavelet-LLH_glrlm_RunLengthNonUniformity,wavelet-LLH_glrlm_RunLengthNonUniformityNormalized,wavelet-LLH_glrlm_RunPercentage,wavelet-LLH_glrlm_RunVariance,wavelet-LLH_glrlm_ShortRunEmphasis,wavelet-LLH_glrlm_ShortRunHighGrayLevelEmphasis,wavelet-LLH_glrlm_ShortRunLowGrayLevelEmphasis,wavelet-LLH_glszm_GrayLevelNonUniformity,wavelet-LLH_glszm_GrayLevelNonUniformityNormalized,wavelet-LLH_glszm_GrayLevelVariance,wavelet-LLH_glszm_HighGrayLevelZoneEmphasis,wavelet-LLH_glszm_LargeAreaEmphasis,wavelet-LLH_glszm_LargeAreaHighGrayLevelEmphasis,wavelet-LLH_glszm_LargeAreaLowGrayLevelEmphasis,wavelet-LLH_glszm_LowGrayLevelZoneEmphasis,wavelet-LLH_glszm_SizeZoneNonUniformity,wavelet-LLH_glszm_SizeZoneNonUniformityNormalized,wavelet-LLH_glszm_SmallAreaEmphasis,wavelet-LLH_glszm_SmallAreaHighGrayLevelEmphasis,wavelet-LLH_glszm_SmallAreaLowGrayLevelEmphasis,wavelet-LLH_glszm_ZoneEntropy,wavelet-LLH_glszm_ZonePercentage,wavelet-LLH_glszm_ZoneVariance,wavelet-LLH_gldm_DependenceEntropy,wavelet-LLH_gldm_DependenceNonUniformity,wavelet-LLH_gldm_DependenceNonUniformityNormalized,wavelet-LLH_gldm_DependenceVariance,wavelet-LLH_gldm_GrayLevelNonUniformity,wavelet-LLH_gldm_GrayLevelVariance,wavelet-LLH_gldm_HighGrayLevelEmphasis,wavelet-LLH_gldm_LargeDependenceEmphasis,wavelet-LLH_gldm_LargeDependenceHighGrayLevelEmphasis,wavelet-LLH_gldm_LargeDependenceLowGrayLevelEmphasis,wavelet-LLH_gldm_LowGrayLevelEmphasis,wavelet-LLH_gldm_SmallDependenceEmphasis,wavelet-LLH_gldm_SmallDependenceHighGrayLevelEmphasis,wavelet-LLH_gldm_SmallDependenceLowGrayLevelEmphasis,wavelet-LLH_ngtdm_Busyness,wavelet-LLH_ngtdm_Coarseness,wavelet-LLH_ngtdm_Complexity,wavelet-LLH_ngtdm_Contrast,wavelet-LLH_ngtdm_Strength,wavelet-LHL_firstorder_10Percentile,wavelet-LHL_firstorder_90Percentile,wavelet-LHL_firstorder_Energy,wavelet-LHL_firstorder_Entropy,wavelet-LHL_firstorder_InterquartileRange,wavelet-LHL_firstorder_Kurtosis,wavelet-LHL_firstorder_Maximum,wavelet-LHL_firstorder_MeanAbsoluteDeviation,wavelet-LHL_firstorder_Mean,wavelet-LHL_firstorder_Median,wavelet-LHL_firstorder_Minimum,wavelet-LHL_firstorder_Range,wavelet-LHL_firstorder_RobustMeanAbsoluteDeviation,wavelet-LHL_firstorder_RootMeanSquared,wavelet-LHL_firstorder_Skewness,wavelet-LHL_firstorder_TotalEnergy,wavelet-LHL_firstorder_Uniformity,wavelet-LHL_firstorder_Variance,wavelet-LHL_glcm_Autocorrelation,wavelet-LHL_glcm_JointAverage,wavelet-LHL_glcm_ClusterProminence,wavelet-LHL_glcm_ClusterShade,wavelet-LHL_glcm_ClusterTendency,wavelet-LHL_glcm_Contrast,wavelet-LHL_glcm_Correlation,wavelet-LHL_glcm_DifferenceAverage,wavelet-LHL_glcm_DifferenceEntropy,wavelet-LHL_glcm_DifferenceVariance,wavelet-LHL_glcm_JointEnergy,wavelet-LHL_glcm_JointEntropy,wavelet-LHL_glcm_Imc1,wavelet-LHL_glcm_Imc2,wavelet-LHL_glcm_Idm,wavelet-LHL_glcm_Idmn,wavelet-LHL_glcm_Id,wavelet-LHL_glcm_Idn,wavelet-LHL_glcm_InverseVariance,wavelet-LHL_glcm_MaximumProbability,wavelet-LHL_glcm_SumEntropy,wavelet-LHL_glcm_SumSquares,wavelet-LHL_glrlm_GrayLevelNonUniformity,wavelet-LHL_glrlm_GrayLevelNonUniformityNormalized,wavelet-LHL_glrlm_GrayLevelVariance,wavelet-LHL_glrlm_HighGrayLevelRunEmphasis,wavelet-LHL_glrlm_LongRunEmphasis,wavelet-LHL_glrlm_LongRunHighGrayLevelEmphasis,wavelet-LHL_glrlm_LongRunLowGrayLevelEmphasis,wavelet-LHL_glrlm_LowGrayLevelRunEmphasis,wavelet-LHL_glrlm_RunEntropy,wavelet-LHL_glrlm_RunLengthNonUniformity,wavelet-LHL_glrlm_RunLengthNonUniformityNormalized,wavelet-LHL_glrlm_RunPercentage,wavelet-LHL_glrlm_RunVariance,wavelet-LHL_glrlm_ShortRunEmphasis,wavelet-LHL_glrlm_ShortRunHighGrayLevelEmphasis,wavelet-LHL_glrlm_ShortRunLowGrayLevelEmphasis,wavelet-LHL_glszm_GrayLevelNonUniformity,wavelet-LHL_glszm_GrayLevelNonUniformityNormalized,wavelet-LHL_glszm_GrayLevelVariance,wavelet-LHL_glszm_HighGrayLevelZoneEmphasis,wavelet-LHL_glszm_LargeAreaEmphasis,wavelet-LHL_glszm_LargeAreaHighGrayLevelEmphasis,wavelet-LHL_glszm_LargeAreaLowGrayLevelEmphasis,wavelet-LHL_glszm_LowGrayLevelZoneEmphasis,wavelet-LHL_glszm_SizeZoneNonUniformity,wavelet-LHL_glszm_SizeZoneNonUniformityNormalized,wavelet-LHL_glszm_SmallAreaEmphasis,wavelet-LHL_glszm_SmallAreaHighGrayLevelEmphasis,wavelet-LHL_glszm_SmallAreaLowGrayLevelEmphasis,wavelet-LHL_glszm_ZoneEntropy,wavelet-LHL_glszm_ZonePercentage,wavelet-LHL_glszm_ZoneVariance,wavelet-LHL_gldm_DependenceEntropy,wavelet-LHL_gldm_DependenceNonUniformity,wavelet-LHL_gldm_DependenceNonUniformityNormalized,wavelet-LHL_gldm_DependenceVariance,wavelet-LHL_gldm_GrayLevelNonUniformity,wavelet-LHL_gldm_GrayLevelVariance,wavelet-LHL_gldm_HighGrayLevelEmphasis,wavelet-LHL_gldm_LargeDependenceEmphasis,wavelet-LHL_gldm_LargeDependenceHighGrayLevelEmphasis,wavelet-LHL_gldm_LargeDependenceLowGrayLevelEmphasis,wavelet-LHL_gldm_LowGrayLevelEmphasis,wavelet-LHL_gldm_SmallDependenceEmphasis,wavelet-LHL_gldm_SmallDependenceHighGrayLevelEmphasis,wavelet-LHL_gldm_SmallDependenceLowGrayLevelEmphasis,wavelet-LHL_ngtdm_Busyness,wavelet-LHL_ngtdm_Coarseness,wavelet-LHL_ngtdm_Complexity,wavelet-LHL_ngtdm_Contrast,wavelet-LHL_ngtdm_Strength,wavelet-LHH_firstorder_10Percentile,wavelet-LHH_firstorder_90Percentile,wavelet-LHH_firstorder_Energy,wavelet-LHH_firstorder_Entropy,wavelet-LHH_firstorder_InterquartileRange,wavelet-LHH_firstorder_Kurtosis,wavelet-LHH_firstorder_Maximum,wavelet-LHH_firstorder_MeanAbsoluteDeviation,wavelet-LHH_firstorder_Mean,wavelet-LHH_firstorder_Median,wavelet-LHH_firstorder_Minimum,wavelet-LHH_firstorder_Range,wavelet-LHH_firstorder_RobustMeanAbsoluteDeviation,wavelet-LHH_firstorder_RootMeanSquared,wavelet-LHH_firstorder_Skewness,wavelet-LHH_firstorder_TotalEnergy,wavelet-LHH_firstorder_Uniformity,wavelet-LHH_firstorder_Variance,wavelet-LHH_glcm_Autocorrelation,wavelet-LHH_glcm_JointAverage,wavelet-LHH_glcm_ClusterProminence,wavelet-LHH_glcm_ClusterShade,wavelet-LHH_glcm_ClusterTendency,wavelet-LHH_glcm_Contrast,wavelet-LHH_glcm_Correlation,wavelet-LHH_glcm_DifferenceAverage,wavelet-LHH_glcm_DifferenceEntropy,wavelet-LHH_glcm_DifferenceVariance,wavelet-LHH_glcm_JointEnergy,wavelet-LHH_glcm_JointEntropy,wavelet-LHH_glcm_Imc1,wavelet-LHH_glcm_Imc2,wavelet-LHH_glcm_Idm,wavelet-LHH_glcm_Idmn,wavelet-LHH_glcm_Id,wavelet-LHH_glcm_Idn,wavelet-LHH_glcm_InverseVariance,wavelet-LHH_glcm_MaximumProbability,wavelet-LHH_glcm_SumEntropy,wavelet-LHH_glcm_SumSquares,wavelet-LHH_glrlm_GrayLevelNonUniformity,wavelet-LHH_glrlm_GrayLevelNonUniformityNormalized,wavelet-LHH_glrlm_GrayLevelVariance,wavelet-LHH_glrlm_HighGrayLevelRunEmphasis,wavelet-LHH_glrlm_LongRunEmphasis,wavelet-LHH_glrlm_LongRunHighGrayLevelEmphasis,wavelet-LHH_glrlm_LongRunLowGrayLevelEmphasis,wavelet-LHH_glrlm_LowGrayLevelRunEmphasis,wavelet-LHH_glrlm_RunEntropy,wavelet-LHH_glrlm_RunLengthNonUniformity,wavelet-LHH_glrlm_RunLengthNonUniformityNormalized,wavelet-LHH_glrlm_RunPercentage,wavelet-LHH_glrlm_RunVariance,wavelet-LHH_glrlm_ShortRunEmphasis,wavelet-LHH_glrlm_ShortRunHighGrayLevelEmphasis,wavelet-LHH_glrlm_ShortRunLowGrayLevelEmphasis,wavelet-LHH_glszm_GrayLevelNonUniformity,wavelet-LHH_glszm_GrayLevelNonUniformityNormalized,wavelet-LHH_glszm_GrayLevelVariance,wavelet-LHH_glszm_HighGrayLevelZoneEmphasis,wavelet-LHH_glszm_LargeAreaEmphasis,wavelet-LHH_glszm_LargeAreaHighGrayLevelEmphasis,wavelet-LHH_glszm_LargeAreaLowGrayLevelEmphasis,wavelet-LHH_glszm_LowGrayLevelZoneEmphasis,wavelet-LHH_glszm_SizeZoneNonUniformity,wavelet-LHH_glszm_SizeZoneNonUniformityNormalized,wavelet-LHH_glszm_SmallAreaEmphasis,wavelet-LHH_glszm_SmallAreaHighGrayLevelEmphasis,wavelet-LHH_glszm_SmallAreaLowGrayLevelEmphasis,wavelet-LHH_glszm_ZoneEntropy,wavelet-LHH_glszm_ZonePercentage,wavelet-LHH_glszm_ZoneVariance,wavelet-LHH_gldm_DependenceEntropy,wavelet-LHH_gldm_DependenceNonUniformity,wavelet-LHH_gldm_DependenceNonUniformityNormalized,wavelet-LHH_gldm_DependenceVariance,wavelet-LHH_gldm_GrayLevelNonUniformity,wavelet-LHH_gldm_GrayLevelVariance,wavelet-LHH_gldm_HighGrayLevelEmphasis,wavelet-LHH_gldm_LargeDependenceEmphasis,wavelet-LHH_gldm_LargeDependenceHighGrayLevelEmphasis,wavelet-LHH_gldm_LargeDependenceLowGrayLevelEmphasis,wavelet-LHH_gldm_LowGrayLevelEmphasis,wavelet-LHH_gldm_SmallDependenceEmphasis,wavelet-LHH_gldm_SmallDependenceHighGrayLevelEmphasis,wavelet-LHH_gldm_SmallDependenceLowGrayLevelEmphasis,wavelet-LHH_ngtdm_Busyness,wavelet-LHH_ngtdm_Coarseness,wavelet-LHH_ngtdm_Complexity,wavelet-LHH_ngtdm_Contrast,wavelet-LHH_ngtdm_Strength,wavelet-HLL_firstorder_10Percentile,wavelet-HLL_firstorder_90Percentile,wavelet-HLL_firstorder_Energy,wavelet-HLL_firstorder_Entropy,wavelet-HLL_firstorder_InterquartileRange,wavelet-HLL_firstorder_Kurtosis,wavelet-HLL_firstorder_Maximum,wavelet-HLL_firstorder_MeanAbsoluteDeviation,wavelet-HLL_firstorder_Mean,wavelet-HLL_firstorder_Median,wavelet-HLL_firstorder_Minimum,wavelet-HLL_firstorder_Range,wavelet-HLL_firstorder_RobustMeanAbsoluteDeviation,wavelet-HLL_firstorder_RootMeanSquared,wavelet-HLL_firstorder_Skewness,wavelet-HLL_firstorder_TotalEnergy,wavelet-HLL_firstorder_Uniformity,wavelet-HLL_firstorder_Variance,wavelet-HLL_glcm_Autocorrelation,wavelet-HLL_glcm_JointAverage,wavelet-HLL_glcm_ClusterProminence,wavelet-HLL_glcm_ClusterShade,wavelet-HLL_glcm_ClusterTendency,wavelet-HLL_glcm_Contrast,wavelet-HLL_glcm_Correlation,wavelet-HLL_glcm_DifferenceAverage,wavelet-HLL_glcm_DifferenceEntropy,wavelet-HLL_glcm_DifferenceVariance,wavelet-HLL_glcm_JointEnergy,wavelet-HLL_glcm_JointEntropy,wavelet-HLL_glcm_Imc1,wavelet-HLL_glcm_Imc2,wavelet-HLL_glcm_Idm,wavelet-HLL_glcm_Idmn,wavelet-HLL_glcm_Id,wavelet-HLL_glcm_Idn,wavelet-HLL_glcm_InverseVariance,wavelet-HLL_glcm_MaximumProbability,wavelet-HLL_glcm_SumEntropy,wavelet-HLL_glcm_SumSquares,wavelet-HLL_glrlm_GrayLevelNonUniformity,wavelet-HLL_glrlm_GrayLevelNonUniformityNormalized,wavelet-HLL_glrlm_GrayLevelVariance,wavelet-HLL_glrlm_HighGrayLevelRunEmphasis,wavelet-HLL_glrlm_LongRunEmphasis,wavelet-HLL_glrlm_LongRunHighGrayLevelEmphasis,wavelet-HLL_glrlm_LongRunLowGrayLevelEmphasis,wavelet-HLL_glrlm_LowGrayLevelRunEmphasis,wavelet-HLL_glrlm_RunEntropy,wavelet-HLL_glrlm_RunLengthNonUniformity,wavelet-HLL_glrlm_RunLengthNonUniformityNormalized,wavelet-HLL_glrlm_RunPercentage,wavelet-HLL_glrlm_RunVariance,wavelet-HLL_glrlm_ShortRunEmphasis,wavelet-HLL_glrlm_ShortRunHighGrayLevelEmphasis,wavelet-HLL_glrlm_ShortRunLowGrayLevelEmphasis,wavelet-HLL_glszm_GrayLevelNonUniformity,wavelet-HLL_glszm_GrayLevelNonUniformityNormalized,wavelet-HLL_glszm_GrayLevelVariance,wavelet-HLL_glszm_HighGrayLevelZoneEmphasis,wavelet-HLL_glszm_LargeAreaEmphasis,wavelet-HLL_glszm_LargeAreaHighGrayLevelEmphasis,wavelet-HLL_glszm_LargeAreaLowGrayLevelEmphasis,wavelet-HLL_glszm_LowGrayLevelZoneEmphasis,wavelet-HLL_glszm_SizeZoneNonUniformity,wavelet-HLL_glszm_SizeZoneNonUniformityNormalized,wavelet-HLL_glszm_SmallAreaEmphasis,wavelet-HLL_glszm_SmallAreaHighGrayLevelEmphasis,wavelet-HLL_glszm_SmallAreaLowGrayLevelEmphasis,wavelet-HLL_glszm_ZoneEntropy,wavelet-HLL_glszm_ZonePercentage,wavelet-HLL_glszm_ZoneVariance,wavelet-HLL_gldm_DependenceEntropy,wavelet-HLL_gldm_DependenceNonUniformity,wavelet-HLL_gldm_DependenceNonUniformityNormalized,wavelet-HLL_gldm_DependenceVariance,wavelet-HLL_gldm_GrayLevelNonUniformity,wavelet-HLL_gldm_GrayLevelVariance,wavelet-HLL_gldm_HighGrayLevelEmphasis,wavelet-HLL_gldm_LargeDependenceEmphasis,wavelet-HLL_gldm_LargeDependenceHighGrayLevelEmphasis,wavelet-HLL_gldm_LargeDependenceLowGrayLevelEmphasis,wavelet-HLL_gldm_LowGrayLevelEmphasis,wavelet-HLL_gldm_SmallDependenceEmphasis,wavelet-HLL_gldm_SmallDependenceHighGrayLevelEmphasis,wavelet-HLL_gldm_SmallDependenceLowGrayLevelEmphasis,wavelet-HLL_ngtdm_Busyness,wavelet-HLL_ngtdm_Coarseness,wavelet-HLL_ngtdm_Complexity,wavelet-HLL_ngtdm_Contrast,wavelet-HLL_ngtdm_Strength,wavelet-HLH_firstorder_10Percentile,wavelet-HLH_firstorder_90Percentile,wavelet-HLH_firstorder_Energy,wavelet-HLH_firstorder_Entropy,wavelet-HLH_firstorder_InterquartileRange,wavelet-HLH_firstorder_Kurtosis,wavelet-HLH_firstorder_Maximum,wavelet-HLH_firstorder_MeanAbsoluteDeviation,wavelet-HLH_firstorder_Mean,wavelet-HLH_firstorder_Median,wavelet-HLH_firstorder_Minimum,wavelet-HLH_firstorder_Range,wavelet-HLH_firstorder_RobustMeanAbsoluteDeviation,wavelet-HLH_firstorder_RootMeanSquared,wavelet-HLH_firstorder_Skewness,wavelet-HLH_firstorder_TotalEnergy,wavelet-HLH_firstorder_Uniformity,wavelet-HLH_firstorder_Variance,wavelet-HLH_glcm_Autocorrelation,wavelet-HLH_glcm_JointAverage,wavelet-HLH_glcm_ClusterProminence,wavelet-HLH_glcm_ClusterShade,wavelet-HLH_glcm_ClusterTendency,wavelet-HLH_glcm_Contrast,wavelet-HLH_glcm_Correlation,wavelet-HLH_glcm_DifferenceAverage,wavelet-HLH_glcm_DifferenceEntropy,wavelet-HLH_glcm_DifferenceVariance,wavelet-HLH_glcm_JointEnergy,wavelet-HLH_glcm_JointEntropy,wavelet-HLH_glcm_Imc1,wavelet-HLH_glcm_Imc2,wavelet-HLH_glcm_Idm,wavelet-HLH_glcm_Idmn,wavelet-HLH_glcm_Id,wavelet-HLH_glcm_Idn,wavelet-HLH_glcm_InverseVariance,wavelet-HLH_glcm_MaximumProbability,wavelet-HLH_glcm_SumEntropy,wavelet-HLH_glcm_SumSquares,wavelet-HLH_glrlm_GrayLevelNonUniformity,wavelet-HLH_glrlm_GrayLevelNonUniformityNormalized,wavelet-HLH_glrlm_GrayLevelVariance,wavelet-HLH_glrlm_HighGrayLevelRunEmphasis,wavelet-HLH_glrlm_LongRunEmphasis,wavelet-HLH_glrlm_LongRunHighGrayLevelEmphasis,wavelet-HLH_glrlm_LongRunLowGrayLevelEmphasis,wavelet-HLH_glrlm_LowGrayLevelRunEmphasis,wavelet-HLH_glrlm_RunEntropy,wavelet-HLH_glrlm_RunLengthNonUniformity,wavelet-HLH_glrlm_RunLengthNonUniformityNormalized,wavelet-HLH_glrlm_RunPercentage,wavelet-HLH_glrlm_RunVariance,wavelet-HLH_glrlm_ShortRunEmphasis,wavelet-HLH_glrlm_ShortRunHighGrayLevelEmphasis,wavelet-HLH_glrlm_ShortRunLowGrayLevelEmphasis,wavelet-HLH_glszm_GrayLevelNonUniformity,wavelet-HLH_glszm_GrayLevelNonUniformityNormalized,wavelet-HLH_glszm_GrayLevelVariance,wavelet-HLH_glszm_HighGrayLevelZoneEmphasis,wavelet-HLH_glszm_LargeAreaEmphasis,wavelet-HLH_glszm_LargeAreaHighGrayLevelEmphasis,wavelet-HLH_glszm_LargeAreaLowGrayLevelEmphasis,wavelet-HLH_glszm_LowGrayLevelZoneEmphasis,wavelet-HLH_glszm_SizeZoneNonUniformity,wavelet-HLH_glszm_SizeZoneNonUniformityNormalized,wavelet-HLH_glszm_SmallAreaEmphasis,wavelet-HLH_glszm_SmallAreaHighGrayLevelEmphasis,wavelet-HLH_glszm_SmallAreaLowGrayLevelEmphasis,wavelet-HLH_glszm_ZoneEntropy,wavelet-HLH_glszm_ZonePercentage,wavelet-HLH_glszm_ZoneVariance,wavelet-HLH_gldm_DependenceEntropy,wavelet-HLH_gldm_DependenceNonUniformity,wavelet-HLH_gldm_DependenceNonUniformityNormalized,wavelet-HLH_gldm_DependenceVariance,wavelet-HLH_gldm_GrayLevelNonUniformity,wavelet-HLH_gldm_GrayLevelVariance,wavelet-HLH_gldm_HighGrayLevelEmphasis,wavelet-HLH_gldm_LargeDependenceEmphasis,wavelet-HLH_gldm_LargeDependenceHighGrayLevelEmphasis,wavelet-HLH_gldm_LargeDependenceLowGrayLevelEmphasis,wavelet-HLH_gldm_LowGrayLevelEmphasis,wavelet-HLH_gldm_SmallDependenceEmphasis,wavelet-HLH_gldm_SmallDependenceHighGrayLevelEmphasis,wavelet-HLH_gldm_SmallDependenceLowGrayLevelEmphasis,wavelet-HLH_ngtdm_Busyness,wavelet-HLH_ngtdm_Coarseness,wavelet-HLH_ngtdm_Complexity,wavelet-HLH_ngtdm_Contrast,wavelet-HLH_ngtdm_Strength,wavelet-HHL_firstorder_10Percentile,wavelet-HHL_firstorder_90Percentile,wavelet-HHL_firstorder_Energy,wavelet-HHL_firstorder_Entropy,wavelet-HHL_firstorder_InterquartileRange,wavelet-HHL_firstorder_Kurtosis,wavelet-HHL_firstorder_Maximum,wavelet-HHL_firstorder_MeanAbsoluteDeviation,wavelet-HHL_firstorder_Mean,wavelet-HHL_firstorder_Median,wavelet-HHL_firstorder_Minimum,wavelet-HHL_firstorder_Range,wavelet-HHL_firstorder_RobustMeanAbsoluteDeviation,wavelet-HHL_firstorder_RootMeanSquared,wavelet-HHL_firstorder_Skewness,wavelet-HHL_firstorder_TotalEnergy,wavelet-HHL_firstorder_Uniformity,wavelet-HHL_firstorder_Variance,wavelet-HHL_glcm_Autocorrelation,wavelet-HHL_glcm_JointAverage,wavelet-HHL_glcm_ClusterProminence,wavelet-HHL_glcm_ClusterShade,wavelet-HHL_glcm_ClusterTendency,wavelet-HHL_glcm_Contrast,wavelet-HHL_glcm_Correlation,wavelet-HHL_glcm_DifferenceAverage,wavelet-HHL_glcm_DifferenceEntropy,wavelet-HHL_glcm_DifferenceVariance,wavelet-HHL_glcm_JointEnergy,wavelet-HHL_glcm_JointEntropy,wavelet-HHL_glcm_Imc1,wavelet-HHL_glcm_Imc2,wavelet-HHL_glcm_Idm,wavelet-HHL_glcm_Idmn,wavelet-HHL_glcm_Id,wavelet-HHL_glcm_Idn,wavelet-HHL_glcm_InverseVariance,wavelet-HHL_glcm_MaximumProbability,wavelet-HHL_glcm_SumEntropy,wavelet-HHL_glcm_SumSquares,wavelet-HHL_glrlm_GrayLevelNonUniformity,wavelet-HHL_glrlm_GrayLevelNonUniformityNormalized,wavelet-HHL_glrlm_GrayLevelVariance,wavelet-HHL_glrlm_HighGrayLevelRunEmphasis,wavelet-HHL_glrlm_LongRunEmphasis,wavelet-HHL_glrlm_LongRunHighGrayLevelEmphasis,wavelet-HHL_glrlm_LongRunLowGrayLevelEmphasis,wavelet-HHL_glrlm_LowGrayLevelRunEmphasis,wavelet-HHL_glrlm_RunEntropy,wavelet-HHL_glrlm_RunLengthNonUniformity,wavelet-HHL_glrlm_RunLengthNonUniformityNormalized,wavelet-HHL_glrlm_RunPercentage,wavelet-HHL_glrlm_RunVariance,wavelet-HHL_glrlm_ShortRunEmphasis,wavelet-HHL_glrlm_ShortRunHighGrayLevelEmphasis,wavelet-HHL_glrlm_ShortRunLowGrayLevelEmphasis,wavelet-HHL_glszm_GrayLevelNonUniformity,wavelet-HHL_glszm_GrayLevelNonUniformityNormalized,wavelet-HHL_glszm_GrayLevelVariance,wavelet-HHL_glszm_HighGrayLevelZoneEmphasis,wavelet-HHL_glszm_LargeAreaEmphasis,wavelet-HHL_glszm_LargeAreaHighGrayLevelEmphasis,wavelet-HHL_glszm_LargeAreaLowGrayLevelEmphasis,wavelet-HHL_glszm_LowGrayLevelZoneEmphasis,wavelet-HHL_glszm_SizeZoneNonUniformity,wavelet-HHL_glszm_SizeZoneNonUniformityNormalized,wavelet-HHL_glszm_SmallAreaEmphasis,wavelet-HHL_glszm_SmallAreaHighGrayLevelEmphasis,wavelet-HHL_glszm_SmallAreaLowGrayLevelEmphasis,wavelet-HHL_glszm_ZoneEntropy,wavelet-HHL_glszm_ZonePercentage,wavelet-HHL_glszm_ZoneVariance,wavelet-HHL_gldm_DependenceEntropy,wavelet-HHL_gldm_DependenceNonUniformity,wavelet-HHL_gldm_DependenceNonUniformityNormalized,wavelet-HHL_gldm_DependenceVariance,wavelet-HHL_gldm_GrayLevelNonUniformity,wavelet-HHL_gldm_GrayLevelVariance,wavelet-HHL_gldm_HighGrayLevelEmphasis,wavelet-HHL_gldm_LargeDependenceEmphasis,wavelet-HHL_gldm_LargeDependenceHighGrayLevelEmphasis,wavelet-HHL_gldm_LargeDependenceLowGrayLevelEmphasis,wavelet-HHL_gldm_LowGrayLevelEmphasis,wavelet-HHL_gldm_SmallDependenceEmphasis,wavelet-HHL_gldm_SmallDependenceHighGrayLevelEmphasis,wavelet-HHL_gldm_SmallDependenceLowGrayLevelEmphasis,wavelet-HHL_ngtdm_Busyness,wavelet-HHL_ngtdm_Coarseness,wavelet-HHL_ngtdm_Complexity,wavelet-HHL_ngtdm_Contrast,wavelet-HHL_ngtdm_Strength,wavelet-HHH_firstorder_10Percentile,wavelet-HHH_firstorder_90Percentile,wavelet-HHH_firstorder_Energy,wavelet-HHH_firstorder_Entropy,wavelet-HHH_firstorder_InterquartileRange,wavelet-HHH_firstorder_Kurtosis,wavelet-HHH_firstorder_Maximum,wavelet-HHH_firstorder_MeanAbsoluteDeviation,wavelet-HHH_firstorder_Mean,wavelet-HHH_firstorder_Median,wavelet-HHH_firstorder_Minimum,wavelet-HHH_firstorder_Range,wavelet-HHH_firstorder_RobustMeanAbsoluteDeviation,wavelet-HHH_firstorder_RootMeanSquared,wavelet-HHH_firstorder_Skewness,wavelet-HHH_firstorder_TotalEnergy,wavelet-HHH_firstorder_Uniformity,wavelet-HHH_firstorder_Variance,wavelet-HHH_glcm_Autocorrelation,wavelet-HHH_glcm_JointAverage,wavelet-HHH_glcm_ClusterProminence,wavelet-HHH_glcm_ClusterShade,wavelet-HHH_glcm_ClusterTendency,wavelet-HHH_glcm_Contrast,wavelet-HHH_glcm_Correlation,wavelet-HHH_glcm_DifferenceAverage,wavelet-HHH_glcm_DifferenceEntropy,wavelet-HHH_glcm_DifferenceVariance,wavelet-HHH_glcm_JointEnergy,wavelet-HHH_glcm_JointEntropy,wavelet-HHH_glcm_Imc1,wavelet-HHH_glcm_Imc2,wavelet-HHH_glcm_Idm,wavelet-HHH_glcm_Idmn,wavelet-HHH_glcm_Id,wavelet-HHH_glcm_Idn,wavelet-HHH_glcm_InverseVariance,wavelet-HHH_glcm_MaximumProbability,wavelet-HHH_glcm_SumEntropy,wavelet-HHH_glcm_SumSquares,wavelet-HHH_glrlm_GrayLevelNonUniformity,wavelet-HHH_glrlm_GrayLevelNonUniformityNormalized,wavelet-HHH_glrlm_GrayLevelVariance,wavelet-HHH_glrlm_HighGrayLevelRunEmphasis,wavelet-HHH_glrlm_LongRunEmphasis,wavelet-HHH_glrlm_LongRunHighGrayLevelEmphasis,wavelet-HHH_glrlm_LongRunLowGrayLevelEmphasis,wavelet-HHH_glrlm_LowGrayLevelRunEmphasis,wavelet-HHH_glrlm_RunEntropy,wavelet-HHH_glrlm_RunLengthNonUniformity,wavelet-HHH_glrlm_RunLengthNonUniformityNormalized,wavelet-HHH_glrlm_RunPercentage,wavelet-HHH_glrlm_RunVariance,wavelet-HHH_glrlm_ShortRunEmphasis,wavelet-HHH_glrlm_ShortRunHighGrayLevelEmphasis,wavelet-HHH_glrlm_ShortRunLowGrayLevelEmphasis,wavelet-HHH_glszm_GrayLevelNonUniformity,wavelet-HHH_glszm_GrayLevelNonUniformityNormalized,wavelet-HHH_glszm_GrayLevelVariance,wavelet-HHH_glszm_HighGrayLevelZoneEmphasis,wavelet-HHH_glszm_LargeAreaEmphasis,wavelet-HHH_glszm_LargeAreaHighGrayLevelEmphasis,wavelet-HHH_glszm_LargeAreaLowGrayLevelEmphasis,wavelet-HHH_glszm_LowGrayLevelZoneEmphasis,wavelet-HHH_glszm_SizeZoneNonUniformity,wavelet-HHH_glszm_SizeZoneNonUniformityNormalized,wavelet-HHH_glszm_SmallAreaEmphasis,wavelet-HHH_glszm_SmallAreaHighGrayLevelEmphasis,wavelet-HHH_glszm_SmallAreaLowGrayLevelEmphasis,wavelet-HHH_glszm_ZoneEntropy,wavelet-HHH_glszm_ZonePercentage,wavelet-HHH_glszm_ZoneVariance,wavelet-HHH_gldm_DependenceEntropy,wavelet-HHH_gldm_DependenceNonUniformity,wavelet-HHH_gldm_DependenceNonUniformityNormalized,wavelet-HHH_gldm_DependenceVariance,wavelet-HHH_gldm_GrayLevelNonUniformity,wavelet-HHH_gldm_GrayLevelVariance,wavelet-HHH_gldm_HighGrayLevelEmphasis,wavelet-HHH_gldm_LargeDependenceEmphasis,wavelet-HHH_gldm_LargeDependenceHighGrayLevelEmphasis,wavelet-HHH_gldm_LargeDependenceLowGrayLevelEmphasis,wavelet-HHH_gldm_LowGrayLevelEmphasis,wavelet-HHH_gldm_SmallDependenceEmphasis,wavelet-HHH_gldm_SmallDependenceHighGrayLevelEmphasis,wavelet-HHH_gldm_SmallDependenceLowGrayLevelEmphasis,wavelet-HHH_ngtdm_Busyness,wavelet-HHH_ngtdm_Coarseness,wavelet-HHH_ngtdm_Complexity,wavelet-HHH_ngtdm_Contrast,wavelet-HHH_ngtdm_Strength,wavelet-LLL_firstorder_10Percentile,wavelet-LLL_firstorder_90Percentile,wavelet-LLL_firstorder_Energy,wavelet-LLL_firstorder_Entropy,wavelet-LLL_firstorder_InterquartileRange,wavelet-LLL_firstorder_Kurtosis,wavelet-LLL_firstorder_Maximum,wavelet-LLL_firstorder_MeanAbsoluteDeviation,wavelet-LLL_firstorder_Mean,wavelet-LLL_firstorder_Median,wavelet-LLL_firstorder_Minimum,wavelet-LLL_firstorder_Range,wavelet-LLL_firstorder_RobustMeanAbsoluteDeviation,wavelet-LLL_firstorder_RootMeanSquared,wavelet-LLL_firstorder_Skewness,wavelet-LLL_firstorder_TotalEnergy,wavelet-LLL_firstorder_Uniformity,wavelet-LLL_firstorder_Variance,wavelet-LLL_glcm_Autocorrelation,wavelet-LLL_glcm_JointAverage,wavelet-LLL_glcm_ClusterProminence,wavelet-LLL_glcm_ClusterShade,wavelet-LLL_glcm_ClusterTendency,wavelet-LLL_glcm_Contrast,wavelet-LLL_glcm_Correlation,wavelet-LLL_glcm_DifferenceAverage,wavelet-LLL_glcm_DifferenceEntropy,wavelet-LLL_glcm_DifferenceVariance,wavelet-LLL_glcm_JointEnergy,wavelet-LLL_glcm_JointEntropy,wavelet-LLL_glcm_Imc1,wavelet-LLL_glcm_Imc2,wavelet-LLL_glcm_Idm,wavelet-LLL_glcm_Idmn,wavelet-LLL_glcm_Id,wavelet-LLL_glcm_Idn,wavelet-LLL_glcm_InverseVariance,wavelet-LLL_glcm_MaximumProbability,wavelet-LLL_glcm_SumEntropy,wavelet-LLL_glcm_SumSquares,wavelet-LLL_glrlm_GrayLevelNonUniformity,wavelet-LLL_glrlm_GrayLevelNonUniformityNormalized,wavelet-LLL_glrlm_GrayLevelVariance,wavelet-LLL_glrlm_HighGrayLevelRunEmphasis,wavelet-LLL_glrlm_LongRunEmphasis,wavelet-LLL_glrlm_LongRunHighGrayLevelEmphasis,wavelet-LLL_glrlm_LongRunLowGrayLevelEmphasis,wavelet-LLL_glrlm_LowGrayLevelRunEmphasis,wavelet-LLL_glrlm_RunEntropy,wavelet-LLL_glrlm_RunLengthNonUniformity,wavelet-LLL_glrlm_RunLengthNonUniformityNormalized,wavelet-LLL_glrlm_RunPercentage,wavelet-LLL_glrlm_RunVariance,wavelet-LLL_glrlm_ShortRunEmphasis,wavelet-LLL_glrlm_ShortRunHighGrayLevelEmphasis,wavelet-LLL_glrlm_ShortRunLowGrayLevelEmphasis,wavelet-LLL_glszm_GrayLevelNonUniformity,wavelet-LLL_glszm_GrayLevelNonUniformityNormalized,wavelet-LLL_glszm_GrayLevelVariance,wavelet-LLL_glszm_HighGrayLevelZoneEmphasis,wavelet-LLL_glszm_LargeAreaEmphasis,wavelet-LLL_glszm_LargeAreaHighGrayLevelEmphasis,wavelet-LLL_glszm_LargeAreaLowGrayLevelEmphasis,wavelet-LLL_glszm_LowGrayLevelZoneEmphasis,wavelet-LLL_glszm_SizeZoneNonUniformity,wavelet-LLL_glszm_SizeZoneNonUniformityNormalized,wavelet-LLL_glszm_SmallAreaEmphasis,wavelet-LLL_glszm_SmallAreaHighGrayLevelEmphasis,wavelet-LLL_glszm_SmallAreaLowGrayLevelEmphasis,wavelet-LLL_glszm_ZoneEntropy,wavelet-LLL_glszm_ZonePercentage,wavelet-LLL_glszm_ZoneVariance,wavelet-LLL_gldm_DependenceEntropy,wavelet-LLL_gldm_DependenceNonUniformity,wavelet-LLL_gldm_DependenceNonUniformityNormalized,wavelet-LLL_gldm_DependenceVariance,wavelet-LLL_gldm_GrayLevelNonUniformity,wavelet-LLL_gldm_GrayLevelVariance,wavelet-LLL_gldm_HighGrayLevelEmphasis,wavelet-LLL_gldm_LargeDependenceEmphasis,wavelet-LLL_gldm_LargeDependenceHighGrayLevelEmphasis,wavelet-LLL_gldm_LargeDependenceLowGrayLevelEmphasis,wavelet-LLL_gldm_LowGrayLevelEmphasis,wavelet-LLL_gldm_SmallDependenceEmphasis,wavelet-LLL_gldm_SmallDependenceHighGrayLevelEmphasis,wavelet-LLL_gldm_SmallDependenceLowGrayLevelEmphasis,wavelet-LLL_ngtdm_Busyness,wavelet-LLL_ngtdm_Coarseness,wavelet-LLL_ngtdm_Complexity,wavelet-LLL_ngtdm_Contrast,wavelet-LLL_ngtdm_Strength

# Features (ICC>0.90)

- 1. **Total 587 features in UECT**

original_shape_SurfaceArea,original_shape_SurfaceVolumeRatio,original_shape_VoxelVolume,original_glcm_SumEntropy,wavelet-LLL_glcm_SumEntropy,wavelet-LLL_glrlm_LongRunLowGrayLevelEmphasis,original_glrlm_LongRunLowGrayLevelEmphasis,wavelet-HHL_glcm_Idm,wavelet-LLH_ngtdm_Contrast,wavelet-HHH_glrlm_GrayLevelNonUniformityNormalized,wavelet-LLH_gldm_LargeDependenceLowGrayLevelEmphasis,wavelet-HHH_glrlm_RunLengthNonUniformity,wavelet-LHH_glrlm_GrayLevelNonUniformityNormalized,wavelet-LLL_glrlm_RunLengthNonUniformityNormalized,wavelet-LHH_glcm_MaximumProbability,wavelet-HHH_glcm_MaximumProbability,wavelet-LHL_glcm_DifferenceAverage,wavelet-LHH_glrlm_RunLengthNonUniformity,original_glrlm_RunLengthNonUniformityNormalized,wavelet-LLH_gldm_DependenceNonUniformity,wavelet-LLL_glcm_Idm,wavelet-HLH_glrlm_RunLengthNonUniformity,original_glcm_ClusterProminence,original_glrlm_LongRunHighGrayLevelEmphasis,wavelet-HLH_glrlm_GrayLevelNonUniformityNormalized,wavelet-LLL_glrlm_LongRunHighGrayLevelEmphasis,wavelet-HHH_glcm_SumEntropy,wavelet-HLL_glcm_Idm,wavelet-HHH_glrlm_LongRunEmphasis,wavelet-LLH_ngtdm_Complexity,wavelet-LHH_glszm_ZoneVariance,wavelet-LHH_glszm_ZoneEntropy,wavelet-LHH_glcm_SumEntropy,wavelet-LHH_glrlm_LongRunEmphasis,wavelet-LLH_ngtdm_Strength,wavelet-HHL_glrlm_GrayLevelNonUniformityNormalized,wavelet-HHL_glrlm_RunLengthNonUniformity,wavelet-LHH_glszm_LargeAreaLowGrayLevelEmphasis,wavelet-LHH_firstorder_Maximum,wavelet-LLH_ngtdm_Coarseness,wavelet-LHH_gldm_LargeDependenceLowGrayLevelEmphasis,wavelet-HLH_glrlm_LongRunEmphasis,wavelet-LHH_glszm_SizeZoneNonUniformityNormalized,wavelet-LHH_ngtdm_Strength,wavelet-HLH_gldm_LargeDependenceLowGrayLevelEmphasis,wavelet-HHL_glcm_SumEntropy,wavelet-LLH_glszm_ZoneVariance,original_glcm_MaximumProbability,wavelet-HHL_glcm_JointEntropy,wavelet-LLL_firstorder_Uniformity,wavelet-HLL_glcm_SumEntropy,wavelet-LLL_glcm_JointEntropy,wavelet-LHH_glrlm_ShortRunEmphasis,log-sigma-5-0-mm-3D_ngtdm_Contrast,wavelet-HHL_firstorder_Uniformity,wavelet-LLH_glszm_ZoneEntropy,wavelet-LHH_glszm_LargeAreaEmphasis,wavelet-LHH_glszm_SmallAreaEmphasis,wavelet-HHH_glszm_GrayLevelVariance,wavelet-LHH_gldm_DependenceNonUniformityNormalized,wavelet-LHL_glcm_SumEntropy,wavelet-LHH_ngtdm_Coarseness,wavelet-LHH_glcm_DifferenceAverage,wavelet-LLL_glcm_MaximumProbability,log-sigma-2-0-mm-3D_glrlm_LongRunLowGrayLevelEmphasis,wavelet-HLL_glrlm_RunLengthNonUniformity,wavelet-LHH_glszm_LowGrayLevelZoneEmphasis,wavelet-HLH_glcm_SumEntropy,wavelet-HHH_gldm_DependenceNonUniformityNormalized,wavelet-LHL_gldm_LargeDependenceLowGrayLevelEmphasis,wavelet-HHH_glszm_SizeZoneNonUniformityNormalized,wavelet-HLH_ngtdm_Strength,wavelet-HHH_glrlm_ShortRunHighGrayLevelEmphasis,wavelet-LHL_ngtdm_Contrast,wavelet-LHH_gldm_DependenceNonUniformity,wavelet-HHL_glszm_ZoneVariance,wavelet-HLH_glszm_ZoneVariance,wavelet-LHL_ngtdm_Complexity,wavelet-LLH_gldm_DependenceNonUniformityNormalized,wavelet-HHH_glszm_ZoneVariance,wavelet-LHL_glcm_Idm,wavelet-LLH_glcm_SumEntropy,wavelet-HHL_glcm_MaximumProbability,wavelet-HLL_ngtdm_Complexity,wavelet-HLH_glszm_ZoneEntropy,wavelet-LHH_gldm_HighGrayLevelEmphasis,wavelet-HLL_gldm_LargeDependenceLowGrayLevelEmphasis,wavelet-HHL_glszm_ZoneEntropy,wavelet-HHH_glszm_ZoneEntropy,wavelet-HLL_ngtdm_Contrast,wavelet-HLL_firstorder_Uniformity,wavelet-HLL_glcm_MaximumProbability,wavelet-HLH_glcm_DifferenceAverage,wavelet-HHL_gldm_LargeDependenceLowGrayLevelEmphasis,wavelet-HLL_glcm_DifferenceAverage,wavelet-HLL_glcm_ClusterProminence,wavelet-LHH_ngtdm_Complexity,wavelet-HHH_glszm_SmallAreaHighGrayLevelEmphasis,wavelet-HLH_ngtdm_Coarseness,wavelet-LHH_glrlm_GrayLevelNonUniformity,wavelet-HLL_glcm_JointEntropy,wavelet-LHH_gldm_DependenceVariance,log-sigma-2-0-mm-3D_glcm_SumEntropy,wavelet-HLH_ngtdm_Complexity,wavelet-HHL_glcm_DifferenceAverage,wavelet-HLH_gldm_DependenceNonUniformity,wavelet-HHL_gldm_DependenceNonUniformityNormalized,wavelet-HLL_ngtdm_Coarseness,wavelet-HHH_glszm_LargeAreaLowGrayLevelEmphasis,wavelet-LLH_glszm_GrayLevelVariance,wavelet-LHL_ngtdm_Coarseness,wavelet-HLH_glszm_SizeZoneNonUniformityNormalized,wavelet-LLH_glrlm_GrayLevelNonUniformityNormalized,wavelet-HHH_glszm_SmallAreaEmphasis,wavelet-HLH_ngtdm_Contrast,wavelet-HHL_ngtdm_Contrast,wavelet-LHH_gldm_GrayLevelNonUniformity,wavelet-HHH_gldm_DependenceNonUniformity,wavelet-HHH_gldm_DependenceVariance,wavelet-HHH_glszm_LowGrayLevelZoneEmphasis,wavelet-LHL_ngtdm_Strength,wavelet-LHH_ngtdm_Contrast,wavelet-LHL_glrlm_GrayLevelNonUniformityNormalized,wavelet-LLH_gldm_SmallDependenceEmphasis,log-sigma-4-0-mm-3D_ngtdm_Contrast,log-sigma-5-0-mm-3D_gldm_LowGrayLevelEmphasis,log-sigma-1-0-mm-3D_glcm_ClusterShade,wavelet-HLH_glszm_HighGrayLevelZoneEmphasis,wavelet-LHL_glszm_ZoneVariance,wavelet-LLH_glrlm_RunLengthNonUniformity,wavelet-HHH_gldm_HighGrayLevelEmphasis,wavelet-LHL_firstorder_Uniformity,wavelet-HLL_ngtdm_Strength,wavelet-LHL_glszm_ZoneEntropy,wavelet-HHL_glszm_SizeZoneNonUniformityNormalized,wavelet-LLH_glszm_SmallAreaHighGrayLevelEmphasis,wavelet-LHL_glrlm_RunLengthNonUniformity,log-sigma-1-0-mm-3D_glcm_DifferenceAverage,wavelet-HLH_glrlm_ShortRunEmphasis,wavelet-HHH_glrlm_ShortRunEmphasis,wavelet-HLH_gldm_DependenceNonUniformityNormalized,log-sigma-5-0-mm-3D_glszm_GrayLevelVariance,log-sigma-3-0-mm-3D_glrlm_LongRunLowGrayLevelEmphasis,wavelet-LLH_glcm_MaximumProbability,log-sigma-3-0-mm-3D_gldm_LargeDependenceLowGrayLevelEmphasis,wavelet-LLH_glszm_SizeZoneNonUniformityNormalized,wavelet-LHH_glszm_SizeZoneNonUniformity,wavelet-HHH_glszm_HighGrayLevelZoneEmphasis,wavelet-LLL_ngtdm_Contrast,wavelet-HLH_glszm_LowGrayLevelZoneEmphasis,wavelet-HHL_glrlm_LongRunEmphasis,original_ngtdm_Complexity,wavelet-HLH_glrlm_ShortRunHighGrayLevelEmphasis,wavelet-HHH_gldm_GrayLevelNonUniformity,wavelet-HHL_glcm_Idn,log-sigma-4-0-mm-3D_glszm_GrayLevelVariance,wavelet-HHL_glcm_Contrast,wavelet-HLH_glszm_LargeAreaLowGrayLevelEmphasis,wavelet-HHL_ngtdm_Coarseness,wavelet-HLL_glcm_ClusterShade,wavelet-HHL_ngtdm_Strength,original_ngtdm_Contrast,wavelet-HLH_gldm_LargeDependenceEmphasis,original_gldm_DependenceNonUniformity,wavelet-LHH_glrlm_ShortRunHighGrayLevelEmphasis,wavelet-HHH_gldm_LargeDependenceLowGrayLevelEmphasis,wavelet-HHH_glszm_LargeAreaEmphasis,log-sigma-4-0-mm-3D_glcm_ClusterShade,wavelet-HHL_glcm_ClusterProminence,wavelet-LLH_glcm_DifferenceEntropy,wavelet-HLL_gldm_DependenceNonUniformityNormalized,wavelet-HHH_glrlm_GrayLevelNonUniformity,wavelet-HHL_glcm_Imc1,wavelet-HHL_glszm_LargeAreaLowGrayLevelEmphasis,wavelet-HLL_glszm_ZoneVariance,wavelet-LHH_glszm_HighGrayLevelZoneEmphasis,wavelet-HLH_glszm_SmallAreaEmphasis,wavelet-HLL_glszm_ZoneEntropy,wavelet-HLH_gldm_HighGrayLevelEmphasis,wavelet-HLL_glcm_Autocorrelation,wavelet-HLH_gldm_DependenceVariance,wavelet-LHL_glcm_JointEntropy,wavelet-LLH_glcm_ClusterShade,log-sigma-3-0-mm-3D_ngtdm_Contrast,wavelet-LHL_gldm_DependenceNonUniformityNormalized,wavelet-LLH_glszm_LargeAreaLowGrayLevelEmphasis,wavelet-HLH_glrlm_GrayLevelNonUniformity,log-sigma-3-0-mm-3D_glcm_SumEntropy,log-sigma-5-0-mm-3D_glcm_DifferenceAverage,wavelet-LLH_glrlm_LongRunEmphasis,wavelet-LLL_glcm_ClusterShade,wavelet-HLL_glrlm_GrayLevelNonUniformityNormalized,log-sigma-4-0-mm-3D_ngtdm_Complexity,wavelet-LLL_glcm_Autocorrelation,wavelet-HHH_glcm_JointEntropy,wavelet-HHH_gldm_LargeDependenceEmphasis,log-sigma-5-0-mm-3D_glrlm_LongRunLowGrayLevelEmphasis,wavelet-HLH_gldm_GrayLevelNonUniformity,log-sigma-4-0-mm-3D_glcm_DifferenceAverage,wavelet-LLL_glcm_Contrast,wavelet-HHH_glcm_ClusterProminence,wavelet-HHL_glcm_ClusterShade,wavelet-HHH_firstorder_Variance,wavelet-LLH_glrlm_ShortRunHighGrayLevelEmphasis,wavelet-HHH_firstorder_Uniformity,log-sigma-5-0-mm-3D_ngtdm_Complexity,wavelet-HHL_glrlm_ShortRunEmphasis,wavelet-LLH_glszm_HighGrayLevelZoneEmphasis,wavelet-LHL_glcm_ClusterShade,wavelet-LHL_glcm_Contrast,wavelet-LLH_glszm_LowGrayLevelZoneEmphasis,wavelet-LLH_glcm_DifferenceAverage,log-sigma-1-0-mm-3D_glcm_SumEntropy,wavelet-HHL_ngtdm_Complexity,wavelet-LLH_glrlm_LongRunHighGrayLevelEmphasis,wavelet-HHH_glszm_SizeZoneNonUniformity,wavelet-HLH_gldm_SmallDependenceEmphasis,wavelet-HHL_glcm_Autocorrelation,wavelet-LHL_glszm_SizeZoneNonUniformityNormalized,log-sigma-4-0-mm-3D_gldm_LowGrayLevelEmphasis,wavelet-LLH_glrlm_RunLengthNonUniformityNormalized,log-sigma-4-0-mm-3D_glrlm_LongRunLowGrayLevelEmphasis,wavelet-HLL_glcm_Contrast,wavelet-LLH_gldm_LargeDependenceEmphasis,wavelet-HLL_glszm_SizeZoneNonUniformityNormalized,wavelet-HHL_glszm_SmallAreaEmphasis,wavelet-HLH_glcm_MaximumProbability,log-sigma-3-0-mm-3D_ngtdm_Complexity,wavelet-LHL_glszm_LargeAreaLowGrayLevelEmphasis,original_firstorder_Range,wavelet-LLH_glszm_GrayLevelNonUniformityNormalized,wavelet-LLH_gldm_GrayLevelNonUniformity,log-sigma-5-0-mm-3D_gldm_LargeDependenceLowGrayLevelEmphasis,wavelet-HHL_glcm_Id,wavelet-HHH_ngtdm_Strength,wavelet-LHL_gldm_DependenceNonUniformity,log-sigma-5-0-mm-3D_glrlm_LongRunHighGrayLevelEmphasis,wavelet-HHL_glszm_LowGrayLevelZoneEmphasis,log-sigma-5-0-mm-3D_glrlm_RunLengthNonUniformityNormalized,log-sigma-5-0-mm-3D_glcm_ClusterShade,log-sigma-5-0-mm-3D_glcm_DifferenceEntropy,log-sigma-5-0-mm-3D_glrlm_RunPercentage,log-sigma-4-0-mm-3D_glrlm_RunLengthNonUniformityNormalized,wavelet-HHH_glszm_GrayLevelNonUniformityNormalized,wavelet-LLL_ngtdm_Complexity,wavelet-HHL_gldm_HighGrayLevelEmphasis,log-sigma-4-0-mm-3D_glrlm_LongRunHighGrayLevelEmphasis,log-sigma-5-0-mm-3D_glrlm_ShortRunLowGrayLevelEmphasis,wavelet-LLH_glszm_LargeAreaHighGrayLevelEmphasis,log-sigma-4-0-mm-3D_glcm_DifferenceEntropy,wavelet-LHL_glcm_DifferenceVariance,original_glszm_ZoneVariance,wavelet-LLL_glcm_ClusterProminence,wavelet-HHH_glcm_DifferenceAverage,wavelet-LLH_glcm_JointAverage,wavelet-HLL_glszm_GrayLevelVariance,log-sigma-3-0-mm-3D_glszm_GrayLevelVariance,wavelet-LHH_glrlm_RunEntropy,wavelet-HHL_firstorder_InterquartileRange,log-sigma-1-0-mm-3D_gldm_LargeDependenceLowGrayLevelEmphasis,wavelet-LLH_glcm_Correlation,wavelet-LHH_gldm_LargeDependenceEmphasis,wavelet-HLL_glrlm_LongRunEmphasis,original_ngtdm_Coarseness,log-sigma-4-0-mm-3D_glszm_ZoneEntropy,wavelet-LHL_glszm_LowGrayLevelZoneEmphasis,log-sigma-3-0-mm-3D_glrlm_RunLengthNonUniformityNormalized,wavelet-HLL_glszm_HighGrayLevelZoneEmphasis,log-sigma-4-0-mm-3D_gldm_LargeDependenceLowGrayLevelEmphasis,wavelet-LLL_gldm_DependenceNonUniformityNormalized,original_glszm_ZoneEntropy,log-sigma-3-0-mm-3D_gldm_LowGrayLevelEmphasis,original_ngtdm_Strength,log-sigma-4-0-mm-3D_glszm_LargeAreaLowGrayLevelEmphasis,log-sigma-5-0-mm-3D_gldm_DependenceEntropy,wavelet-HLL_glrlm_LongRunHighGrayLevelEmphasis,log-sigma-4-0-mm-3D_glszm_ZoneVariance,wavelet-HLL_glrlm_RunLengthNonUniformityNormalized,log-sigma-1-0-mm-3D_gldm_DependenceNonUniformity,wavelet-HLL_firstorder_InterquartileRange,wavelet-HLL_glrlm_ShortRunEmphasis,log-sigma-3-0-mm-3D_glrlm_LongRunHighGrayLevelEmphasis,wavelet-HLH_glszm_ZonePercentage,log-sigma-1-0-mm-3D_ngtdm_Contrast,log-sigma-5-0-mm-3D_ngtdm_Coarseness,log-sigma-5-0-mm-3D_glcm_JointAverage,wavelet-LLH_glrlm_ShortRunEmphasis,wavelet-HLH_glszm_SizeZoneNonUniformity,wavelet-LHH_glszm_SmallAreaHighGrayLevelEmphasis,wavelet-HLH_glszm_LargeAreaEmphasis,wavelet-LLH_glszm_SmallAreaEmphasis,log-sigma-4-0-mm-3D_glcm_SumEntropy,log-sigma-5-0-mm-3D_glszm_LargeAreaLowGrayLevelEmphasis,wavelet-HLH_glrlm_RunEntropy,log-sigma-2-0-mm-3D_gldm_LargeDependenceLowGrayLevelEmphasis,wavelet-LHH_glszm_LargeAreaHighGrayLevelEmphasis,wavelet-LHH_glszm_SmallAreaLowGrayLevelEmphasis,log-sigma-5-0-mm-3D_glszm_ZoneEntropy,wavelet-HHL_gldm_DependenceVariance,wavelet-HLL_glszm_LargeAreaLowGrayLevelEmphasis,wavelet-LHL_glszm_SmallAreaEmphasis,wavelet-HLL_gldm_GrayLevelNonUniformity,wavelet-HLL_glcm_Id,wavelet-HLL_glszm_GrayLevelNonUniformityNormalized,wavelet-HHL_firstorder_Maximum,wavelet-LHH_glcm_JointEntropy,log-sigma-2-0-mm-3D_glrlm_RunLengthNonUniformityNormalized,log-sigma-4-0-mm-3D_ngtdm_Coarseness,log-sigma-2-0-mm-3D_glrlm_GrayLevelNonUniformityNormalized,log-sigma-5-0-mm-3D_glcm_ClusterTendency,wavelet-HLH_glrlm_HighGrayLevelRunEmphasis,wavelet-LLL_glcm_SumSquares,wavelet-LLL_glrlm_RunVariance,wavelet-HHL_glrlm_GrayLevelNonUniformity,log-sigma-5-0-mm-3D_glszm_GrayLevelNonUniformityNormalized,log-sigma-5-0-mm-3D_glszm_ZoneVariance,wavelet-HHL_firstorder_Mean,wavelet-HLL_glrlm_ShortRunHighGrayLevelEmphasis,log-sigma-3-0-mm-3D_glcm_DifferenceAverage,wavelet-HLH_glcm_Contrast,wavelet-HHH_firstorder_Maximum,log-sigma-5-0-mm-3D_gldm_LargeDependenceHighGrayLevelEmphasis,wavelet-LLH_glcm_ClusterTendency,log-sigma-2-0-mm-3D_glrlm_LongRunHighGrayLevelEmphasis,log-sigma-3-0-mm-3D_gldm_DependenceNonUniformity,log-sigma-4-0-mm-3D_glrlm_ShortRunLowGrayLevelEmphasis,wavelet-HLL_glszm_SmallAreaEmphasis,wavelet-LHL_glrlm_ShortRunEmphasis,log-sigma-3-0-mm-3D_glrlm_ShortRunLowGrayLevelEmphasis,wavelet-LLH_glszm_LargeAreaEmphasis,wavelet-HHL_glcm_DifferenceVariance,log-sigma-5-0-mm-3D_glcm_Correlation,log-sigma-4-0-mm-3D_glcm_Correlation,wavelet-LHH_glrlm_HighGrayLevelRunEmphasis,wavelet-HHL_glszm_SmallAreaHighGrayLevelEmphasis,wavelet-HHH_ngtdm_Coarseness,original_gldm_LargeDependenceLowGrayLevelEmphasis,log-sigma-5-0-mm-3D_glszm_HighGrayLevelZoneEmphasis,wavelet-LLH_gldm_HighGrayLevelEmphasis,wavelet-LLH_glszm_SizeZoneNonUniformity,log-sigma-5-0-mm-3D_glszm_LargeAreaEmphasis,log-sigma-5-0-mm-3D_gldm_GrayLevelNonUniformity,log-sigma-2-0-mm-3D_glcm_DifferenceAverage,wavelet-LLH_glrlm_GrayLevelNonUniformity,log-sigma-3-0-mm-3D_glszm_ZoneVariance,wavelet-HHH_glcm_ClusterShade,log-sigma-3-0-mm-3D_glszm_ZoneEntropy,log-sigma-4-0-mm-3D_glszm_GrayLevelNonUniformityNormalized,wavelet-LLH_glcm_Contrast,log-sigma-5-0-mm-3D_glrlm_ShortRunHighGrayLevelEmphasis,wavelet-LHH_firstorder_Uniformity,log-sigma-4-0-mm-3D_gldm_DependenceEntropy,log-sigma-4-0-mm-3D_glszm_LargeAreaEmphasis,log-sigma-2-0-mm-3D_ngtdm_Complexity,wavelet-HHL_firstorder_MeanAbsoluteDeviation,wavelet-HHH_glszm_SmallAreaLowGrayLevelEmphasis,wavelet-LLL_gldm_LargeDependenceLowGrayLevelEmphasis,wavelet-HLH_glcm_JointEntropy,wavelet-LHH_glrlm_LowGrayLevelRunEmphasis,log-sigma-3-0-mm-3D_gldm_LargeDependenceHighGrayLevelEmphasis,log-sigma-5-0-mm-3D_glcm_MaximumProbability,log-sigma-4-0-mm-3D_glcm_ClusterTendency,log-sigma-5-0-mm-3D_gldm_LargeDependenceEmphasis,wavelet-HLH_firstorder_Uniformity,wavelet-HHL_firstorder_Variance,log-sigma-5-0-mm-3D_glszm_GrayLevelNonUniformity,wavelet-HHH_glcm_Autocorrelation,original_gldm_DependenceNonUniformityNormalized,log-sigma-3-0-mm-3D_glszm_LargeAreaLowGrayLevelEmphasis,wavelet-LLL_gldm_DependenceNonUniformity,wavelet-HLL_glszm_LowGrayLevelZoneEmphasis,wavelet-LHH_glszm_GrayLevelNonUniformity,log-sigma-1-0-mm-3D_ngtdm_Complexity,original_glcm_SumSquares,original_glrlm_RunVariance,wavelet-HLH_glszm_GrayLevelNonUniformityNormalized,wavelet-LHL_glrlm_LongRunEmphasis,wavelet-LHH_gldm_SmallDependenceEmphasis,wavelet-LLH_firstorder_10Percentile,wavelet-HLL_gldm_LargeDependenceEmphasis,log-sigma-4-0-mm-3D_firstorder_10Percentile,log-sigma-5-0-mm-3D_glcm_InverseVariance,log-sigma-5-0-mm-3D_glcm_JointEntropy,log-sigma-4-0-mm-3D_gldm_LargeDependenceHighGrayLevelEmphasis,log-sigma-4-0-mm-3D_gldm_DependenceNonUniformity,log-sigma-5-0-mm-3D_firstorder_10Percentile,wavelet-HHL_glszm_LargeAreaEmphasis,log-sigma-4-0-mm-3D_glcm_JointAverage,log-sigma-4-0-mm-3D_glcm_ClusterProminence,wavelet-HHH_ngtdm_Complexity,log-sigma-5-0-mm-3D_glcm_SumEntropy,wavelet-LHL_firstorder_InterquartileRange,wavelet-LLH_glcm_JointEntropy,wavelet-HLL_glrlm_GrayLevelNonUniformity,wavelet-LHH_gldm_SmallDependenceLowGrayLevelEmphasis,wavelet-LLL_glcm_JointAverage,log-sigma-5-0-mm-3D_glszm_SmallAreaHighGrayLevelEmphasis,log-sigma-3-0-mm-3D_glszm_GrayLevelNonUniformityNormalized,wavelet-LLL_glszm_SizeZoneNonUniformityNormalized,wavelet-HLL_gldm_DependenceVariance,log-sigma-2-0-mm-3D_ngtdm_Contrast,log-sigma-5-0-mm-3D_glrlm_GrayLevelNonUniformityNormalized,log-sigma-4-0-mm-3D_glcm_MaximumProbability,log-sigma-1-0-mm-3D_glrlm_LongRunLowGrayLevelEmphasis,wavelet-HHH_ngtdm_Contrast,wavelet-HLL_gldm_HighGrayLevelEmphasis,wavelet-HLH_glszm_LargeAreaHighGrayLevelEmphasis,wavelet-LLH_firstorder_Mean,wavelet-LLH_gldm_SmallDependenceLowGrayLevelEmphasis,log-sigma-4-0-mm-3D_gldm_LargeDependenceEmphasis,log-sigma-5-0-mm-3D_glrlm_RunLengthNonUniformity,wavelet-HHL_glcm_InverseVariance,wavelet-LHH_glszm_GrayLevelNonUniformityNormalized,wavelet-HLH_glszm_SmallAreaHighGrayLevelEmphasis,wavelet-LHL_gldm_HighGrayLevelEmphasis,wavelet-HHL_glcm_JointAverage,wavelet-HHH_firstorder_RobustMeanAbsoluteDeviation,wavelet-LLL_glcm_DifferenceAverage,original_glcm_DifferenceAverage,wavelet-HLH_firstorder_Maximum,wavelet-LHL_glcm_InverseVariance,wavelet-HLH_firstorder_Variance,log-sigma-1-0-mm-3D_gldm_DependenceNonUniformityNormalized,wavelet-HLL_glszm_SmallAreaHighGrayLevelEmphasis,wavelet-HLL_glcm_Imc1,wavelet-HHH_glszm_LargeAreaHighGrayLevelEmphasis,wavelet-LLH_gldm_DependenceVariance,wavelet-HHL_firstorder_Entropy,wavelet-LHH_glcm_ClusterProminence,wavelet-HLH_gldm_DependenceEntropy,log-sigma-2-0-mm-3D_gldm_GrayLevelVariance,wavelet-HLL_glrlm_LongRunLowGrayLevelEmphasis,wavelet-HHL_glcm_ClusterTendency,wavelet-HLL_glcm_JointAverage,wavelet-HLL_gldm_DependenceNonUniformity,log-sigma-3-0-mm-3D_glcm_ClusterProminence,wavelet-LLH_glcm_InverseVariance,wavelet-LHL_glcm_ClusterProminence,log-sigma-4-0-mm-3D_glrlm_ShortRunHighGrayLevelEmphasis,log-sigma-5-0-mm-3D_glcm_Contrast,wavelet-LHL_firstorder_Minimum,log-sigma-2-0-mm-3D_glcm_JointAverage,wavelet-LHH_firstorder_10Percentile,log-sigma-3-0-mm-3D_ngtdm_Coarseness,wavelet-HHH_glcm_JointAverage,wavelet-HLL_glcm_ClusterTendency,log-sigma-5-0-mm-3D_ngtdm_Busyness,original_glszm_LargeAreaLowGrayLevelEmphasis,original_gldm_GrayLevelVariance,wavelet-HLL_firstorder_RootMeanSquared,wavelet-HLL_firstorder_Kurtosis,log-sigma-4-0-mm-3D_glszm_HighGrayLevelZoneEmphasis,log-sigma-5-0-mm-3D_glrlm_LowGrayLevelRunEmphasis,log-sigma-4-0-mm-3D_gldm_GrayLevelNonUniformity,wavelet-LHL_glrlm_GrayLevelNonUniformity,log-sigma-5-0-mm-3D_firstorder_Kurtosis,wavelet-HLL_glcm_DifferenceEntropy,wavelet-LHL_glcm_ClusterTendency,wavelet-HLL_firstorder_10Percentile,wavelet-LHL_glcm_DifferenceEntropy,wavelet-LLH_firstorder_Uniformity,wavelet-HLH_gldm_SmallDependenceLowGrayLevelEmphasis,wavelet-LHH_glcm_Imc1,wavelet-LHH_gldm_LargeDependenceHighGrayLevelEmphasis,wavelet-HLL_glcm_Correlation,wavelet-LHH_glrlm_ShortRunLowGrayLevelEmphasis,log-sigma-3-0-mm-3D_glcm_ClusterTendency,wavelet-LHL_gldm_LargeDependenceHighGrayLevelEmphasis,wavelet-LHL_glrlm_ShortRunLowGrayLevelEmphasis,wavelet-LHH_glcm_Correlation,wavelet-LHH_firstorder_Kurtosis,wavelet-LLL_glcm_ClusterTendency,log-sigma-5-0-mm-3D_glrlm_LongRunEmphasis,log-sigma-4-0-mm-3D_glrlm_RunPercentage,wavelet-LHH_gldm_LowGrayLevelEmphasis,wavelet-LHL_gldm_DependenceVariance,wavelet-LHL_glcm_MaximumProbability,wavelet-HLH_gldm_GrayLevelVariance,wavelet-LLL_firstorder_Median,log-sigma-1-0-mm-3D_ngtdm_Coarseness,wavelet-LHH_firstorder_Variance,log-sigma-1-0-mm-3D_glszm_LargeAreaEmphasis,log-sigma-1-0-mm-3D_glszm_SizeZoneNonUniformity,wavelet-HLH_glcm_ClusterTendency,wavelet-HHL_glrlm_ShortRunHighGrayLevelEmphasis,log-sigma-2-0-mm-3D_glszm_GrayLevelNonUniformityNormalized,log-sigma-3-0-mm-3D_glszm_LargeAreaEmphasis,wavelet-HHH_glcm_ClusterTendency,original_firstorder_InterquartileRange,wavelet-LLL_gldm_GrayLevelVariance,wavelet-HHL_gldm_GrayLevelNonUniformity,log-sigma-1-0-mm-3D_glszm_GrayLevelNonUniformityNormalized,wavelet-LLL_ngtdm_Busyness,wavelet-LHH_firstorder_RootMeanSquared,log-sigma-1-0-mm-3D_firstorder_MeanAbsoluteDeviation,wavelet-HLL_gldm_GrayLevelVariance,log-sigma-1-0-mm-3D_gldm_GrayLevelVariance,wavelet-LHH_gldm_GrayLevelVariance,wavelet-HHH_gldm_GrayLevelVariance,log-sigma-4-0-mm-3D_ngtdm_Busyness,wavelet-HLL_glcm_SumSquares,wavelet-HLL_glrlm_RunVariance,wavelet-HLH_glcm_JointAverage,wavelet-HHH_glcm_SumSquares,wavelet-HHH_glrlm_RunVariance,wavelet-LHH_glcm_SumSquares,wavelet-LHH_glrlm_RunVariance,wavelet-HHL_glcm_SumSquares,wavelet-HHL_glrlm_RunVariance,wavelet-HLH_glcm_SumSquares,wavelet-HLH_glrlm_RunVariance,original_glcm_Imc2,original_firstorder_Uniformity,original_glcm_InverseVariance,wavelet-HHH_gldm_SmallDependenceLowGrayLevelEmphasis,log-sigma-3-0-mm-3D_glcm_JointAverage,log-sigma-1-0-mm-3D_glcm_SumSquares,log-sigma-1-0-mm-3D_glrlm_RunVariance,original_glszm_SizeZoneNonUniformityNormalized,wavelet-LLH_glcm_SumSquares,wavelet-LLH_glrlm_RunVariance,wavelet-LHL_glcm_SumSquares,wavelet-LHL_glrlm_RunVariance,log-sigma-4-0-mm-3D_firstorder_Kurtosis,log-sigma-5-0-mm-3D_firstorder_Uniformity,wavelet-LHL_firstorder_Energy,log-sigma-2-0-mm-3D_glcm_SumSquares,log-sigma-2-0-mm-3D_glrlm_RunVariance,log-sigma-1-0-mm-3D_glszm_ZoneVariance,log-sigma-3-0-mm-3D_glcm_SumSquares,log-sigma-3-0-mm-3D_glrlm_RunVariance,wavelet-LHH_firstorder_Minimum,wavelet-LLL_ngtdm_Coarseness,log-sigma-2-0-mm-3D_glcm_ClusterTendency,wavelet-HLH_glszm_SmallAreaLowGrayLevelEmphasis,wavelet-LLH_firstorder_Kurtosis,wavelet-LLL_glszm_ZoneVariance,log-sigma-5-0-mm-3D_gldm_DependenceNonUniformity,wavelet-LHH_glcm_DifferenceEntropy,wavelet-LLH_glszm_SmallAreaLowGrayLevelEmphasis,log-sigma-3-0-mm-3D_gldm_DependenceEntropy,log-sigma-4-0-mm-3D_glcm_SumSquares,log-sigma-4-0-mm-3D_glrlm_RunVariance,wavelet-HHL_gldm_GrayLevelVariance,wavelet-LHL_ngtdm_Busyness,wavelet-LLH_glszm_GrayLevelNonUniformity,wavelet-LLH_gldm_LowGrayLevelEmphasis,log-sigma-3-0-mm-3D_glszm_GrayLevelNonUniformity,wavelet-LHL_glcm_JointAverage,wavelet-LHH_glcm_JointAverage,wavelet-HHL_firstorder_Energy,log-sigma-4-0-mm-3D_glrlm_LowGrayLevelRunEmphasis,wavelet-HLH_glrlm_LowGrayLevelRunEmphasis,log-sigma-5-0-mm-3D_glcm_Imc1,log-sigma-5-0-mm-3D_glcm_SumSquares,log-sigma-5-0-mm-3D_glrlm_RunVariance,wavelet-LLH_glrlm_LowGrayLevelRunEmphasis,wavelet-LLL_glrlm_HighGrayLevelRunEmphasis,log-sigma-1-0-mm-3D_glszm_ZoneEntropy,log-sigma-1-0-mm-3D_glszm_LargeAreaLowGrayLevelEmphasis,log-sigma-2-0-mm-3D_glszm_LargeAreaLowGrayLevelEmphasis,log-sigma-4-0-mm-3D_glszm_GrayLevelNonUniformity,log-sigma-2-0-mm-3D_glszm_ZoneEntropy,log-sigma-3-0-mm-3D_glcm_MaximumProbability,log-sigma-1-0-mm-3D_ngtdm_Busyness,log-sigma-1-0-mm-3D_glcm_JointAverage,wavelet-HHL_firstorder_Minimum,log-sigma-2-0-mm-3D_glszm_LargeAreaEmphasis,wavelet-LHL_gldm_LowGrayLevelEmphasis,log-sigma-1-0-mm-3D_glrlm_RunLengthNonUniformityNormalized,wavelet-HLL_firstorder_Minimum,log-sigma-2-0-mm-3D_glszm_ZoneVariance,wavelet-HLL_ngtdm_Busyness,log-sigma-1-0-mm-3D_ngtdm_Strength,wavelet-LHH_glcm_ClusterTendency,log-sigma-4-0-mm-3D_glrlm_RunLengthNonUniformity,log-sigma-3-0-mm-3D_gldm_LargeDependenceEmphasis,wavelet-LLL_glszm_ZoneEntropy,log-sigma-4-0-mm-3D_glszm_LowGrayLevelZoneEmphasis,wavelet-HLL_firstorder_Mean,log-sigma-3-0-mm-3D_ngtdm_Busyness,log-sigma-4-0-mm-3D_glcm_JointEntropy,log-sigma-2-0-mm-3D_glrlm_RunLengthNonUniformity,wavelet-LHH_firstorder_Energy,wavelet-HHH_glcm_Imc1,log-sigma-1-0-mm-3D_glrlm_LongRunHighGrayLevelEmphasis,wavelet-LLL_ngtdm_Strength

- 1. **Total 587 features in UECT**

wavelet-HHL_glrlm_RunEntropy,log-sigma-3-0-mm-3D_gldm_SmallDependenceHighGrayLevelEmphasis,wavelet-LLH_firstorder_Maximum,log-sigma-3-0-mm-3D_firstorder_Uniformity,log-sigma-5-0-mm-3D_glcm_JointEnergy,log-sigma-4-0-mm-3D_glszm_SizeZoneNonUniformity,wavelet-HHH_glcm_ClusterShade,log-sigma-2-0-mm-3D_gldm_DependenceEntropy,log-sigma-4-0-mm-3D_glrlm_ShortRunEmphasis,wavelet-LLL_glcm_Imc2,wavelet-HHH_glszm_LargeAreaLowGrayLevelEmphasis,wavelet-LLL_glcm_JointEnergy,log-sigma-4-0-mm-3D_firstorder_Variance,log-sigma-4-0-mm-3D_gldm_GrayLevelVariance,log-sigma-4-0-mm-3D_glszm_GrayLevelNonUniformity,log-sigma-5-0-mm-3D_glrlm_ShortRunEmphasis,log-sigma-1-0-mm-3D_firstorder_Entropy,log-sigma-2-0-mm-3D_glszm_ZonePercentage,wavelet-LLH_glcm_Correlation,log-sigma-1-0-mm-3D_firstorder_90Percentile,log-sigma-1-0-mm-3D_glszm_LargeAreaHighGrayLevelEmphasis,log-sigma-1-0-mm-3D_glcm_JointEntropy,wavelet-HHL_glcm_JointAverage,wavelet-LLL_glrlm_GrayLevelNonUniformityNormalized,wavelet-LHH_firstorder_Kurtosis,log-sigma-2-0-mm-3D_glcm_MaximumProbability,log-sigma-4-0-mm-3D_glcm_ClusterTendency,log-sigma-1-0-mm-3D_glcm_DifferenceAverage,log-sigma-4-0-mm-3D_glcm_JointEnergy,wavelet-LLL_firstorder_Range,log-sigma-2-0-mm-3D_glszm_SmallAreaHighGrayLevelEmphasis,wavelet-LLL_glcm_SumEntropy,log-sigma-5-0-mm-3D_glcm_DifferenceVariance,log-sigma-4-0-mm-3D_glcm_SumSquares,log-sigma-5-0-mm-3D_glcm_Correlation,wavelet-LHH_gldm_LargeDependenceLowGrayLevelEmphasis,log-sigma-5-0-mm-3D_firstorder_Skewness,wavelet-LLH_glszm_SmallAreaHighGrayLevelEmphasis,wavelet-LLL_firstorder_Variance,wavelet-LLL_gldm_GrayLevelVariance,wavelet-LHH_glrlm_LongRunLowGrayLevelEmphasis,wavelet-HHH_firstorder_Maximum,wavelet-HLH_glszm_ZoneEntropy,wavelet-HHH_ngtdm_Strength,original_firstorder_Range,wavelet-LLL_glcm_Imc1,log-sigma-2-0-mm-3D_glcm_JointEnergy,original_glcm_SumEntropy,log-sigma-4-0-mm-3D_glcm_DifferenceVariance,log-sigma-3-0-mm-3D_glrlm_ShortRunEmphasis,log-sigma-4-0-mm-3D_gldm_DependenceNonUniformityNormalized,wavelet-HHL_firstorder_Range,log-sigma-1-0-mm-3D_glcm_DifferenceEntropy,original_firstorder_Entropy,wavelet-HLL_ngtdm_Contrast,wavelet-LLL_glrlm_GrayLevelVariance,wavelet-LLL_firstorder_Entropy,log-sigma-3-0-mm-3D_glszm_LargeAreaHighGrayLevelEmphasis,wavelet-HLL_gldm_DependenceEntropy,log-sigma-2-0-mm-3D_glrlm_RunEntropy,wavelet-LLH_firstorder_Range,original_glszm_GrayLevelVariance,log-sigma-5-0-mm-3D_glszm_GrayLevelNonUniformity,wavelet-HLL_glszm_LargeAreaHighGrayLevelEmphasis,log-sigma-3-0-mm-3D_glcm_DifferenceVariance,log-sigma-1-0-mm-3D_glcm_Contrast,log-sigma-3-0-mm-3D_gldm_LargeDependenceHighGrayLevelEmphasis,wavelet-HHL_firstorder_Maximum,original_glszm_GrayLevelNonUniformityNormalized,wavelet-HHH_firstorder_Skewness,wavelet-LLH_glrlm_ShortRunHighGrayLevelEmphasis,wavelet-LLL_firstorder_Uniformity,wavelet-LLH_firstorder_Minimum,wavelet-LLH_glrlm_HighGrayLevelRunEmphasis,wavelet-LHH_firstorder_Maximum,wavelet-LLH_glcm_JointAverage,wavelet-LLH_gldm_HighGrayLevelEmphasis,wavelet-LHL_firstorder_Minimum,wavelet-LLH_glcm_Autocorrelation,original_firstorder_Maximum,log-sigma-5-0-mm-3D_glcm_DifferenceEntropy,wavelet-LLH_glszm_HighGrayLevelZoneEmphasis,wavelet-LHL_glcm_JointAverage,log-sigma-3-0-mm-3D_glcm_Idmn,log-sigma-2-0-mm-3D_glszm_LargeAreaLowGrayLevelEmphasis,original_firstorder_Variance,log-sigma-4-0-mm-3D_glcm_DifferenceEntropy,original_gldm_GrayLevelVariance,log-sigma-5-0-mm-3D_glcm_Idmn,log-sigma-4-0-mm-3D_glcm_JointEntropy,log-sigma-1-0-mm-3D_firstorder_Uniformity,log-sigma-4-0-mm-3D_firstorder_90Percentile,wavelet-LLH_gldm_SmallDependenceHighGrayLevelEmphasis,log-sigma-3-0-mm-3D_glcm_DifferenceEntropy,wavelet-HLL_firstorder_RobustMeanAbsoluteDeviation,log-sigma-1-0-mm-3D_gldm_SmallDependenceLowGrayLevelEmphasis,log-sigma-1-0-mm-3D_glszm_SizeZoneNonUniformity,log-sigma-3-0-mm-3D_glcm_SumEntropy,log-sigma-3-0-mm-3D_firstorder_Entropy,wavelet-LLL_glcm_JointEntropy,wavelet-HHL_glcm_Correlation,log-sigma-2-0-mm-3D_glrlm_GrayLevelVariance,log-sigma-5-0-mm-3D_glrlm_LowGrayLevelRunEmphasis,original_firstorder_Uniformity,wavelet-LLH_glrlm_LongRunHighGrayLevelEmphasis,log-sigma-2-0-mm-3D_firstorder_Uniformity,wavelet-LHH_firstorder_Minimum,wavelet-LLH_gldm_LargeDependenceHighGrayLevelEmphasis,wavelet-HLL_firstorder_Minimum,log-sigma-4-0-mm-3D_glcm_ClusterShade,wavelet-LHH_firstorder_Range,wavelet-HLL_glcm_JointAverage,log-sigma-2-0-mm-3D_glcm_Idmn,wavelet-HHL_glrlm_GrayLevelVariance,original_glszm_SmallAreaEmphasis,original_glrlm_GrayLevelVariance,log-sigma-2-0-mm-3D_glrlm_ShortRunLowGrayLevelEmphasis,log-sigma-2-0-mm-3D_firstorder_90Percentile,log-sigma-5-0-mm-3D_glszm_SizeZoneNonUniformity,log-sigma-3-0-mm-3D_glcm_ClusterProminence,log-sigma-1-0-mm-3D_glcm_ClusterShade,wavelet-HLL_firstorder_Range,wavelet-HHL_glszm_SmallAreaHighGrayLevelEmphasis,wavelet-LLH_glrlm_RunEntropy,log-sigma-1-0-mm-3D_glcm_Idm,wavelet-LHH_glcm_JointAverage,wavelet-LHL_firstorder_RootMeanSquared,wavelet-LHL_firstorder_Maximum,log-sigma-4-0-mm-3D_glszm_ZonePercentage,original_glszm_SizeZoneNonUniformityNormalized,wavelet-LHL_ngtdm_Busyness,log-sigma-2-0-mm-3D_firstorder_Range,wavelet-HHH_ngtdm_Busyness,wavelet-LHL_firstorder_Mean,log-sigma-5-0-mm-3D_firstorder_RobustMeanAbsoluteDeviation,log-sigma-2-0-mm-3D_ngtdm_Busyness,log-sigma-3-0-mm-3D_glcm_ClusterShade,wavelet-LHL_ngtdm_Contrast,wavelet-LHH_glrlm_LongRunHighGrayLevelEmphasis,wavelet-LLH_ngtdm_Complexity,wavelet-LHL_firstorder_Range,log-sigma-1-0-mm-3D_glcm_Id,wavelet-LLL_glcm_DifferenceEntropy,log-sigma-3-0-mm-3D_firstorder_90Percentile,wavelet-LLL_glcm_DifferenceAverage,log-sigma-3-0-mm-3D_glcm_MaximumProbability,log-sigma-2-0-mm-3D_firstorder_Median,log-sigma-3-0-mm-3D_glrlm_LongRunHighGrayLevelEmphasis,log-sigma-1-0-mm-3D_glcm_DifferenceVariance,original_glszm_LargeAreaHighGrayLevelEmphasis,wavelet-HHH_ngtdm_Complexity,log-sigma-5-0-mm-3D_firstorder_90Percentile,wavelet-HLL_glrlm_LongRunHighGrayLevelEmphasis,wavelet-HLL_glszm_SmallAreaHighGrayLevelEmphasis,log-sigma-1-0-mm-3D_glcm_JointEnergy,original_gldm_SmallDependenceEmphasis,wavelet-HHL_gldm_GrayLevelVariance,original_glcm_JointEntropy,wavelet-HLL_glrlm_RunEntropy,wavelet-LHH_gldm_LargeDependenceHighGrayLevelEmphasis,wavelet-HHL_firstorder_Variance,log-sigma-3-0-mm-3D_glcm_JointEntropy,log-sigma-2-0-mm-3D_glcm_SumEntropy,wavelet-LHL_glcm_Imc2,wavelet-HLL_glrlm_ShortRunHighGrayLevelEmphasis,wavelet-HHH_firstorder_Kurtosis,wavelet-LLL_ngtdm_Strength,wavelet-HLH_glcm_ClusterProminence,log-sigma-2-0-mm-3D_gldm_DependenceNonUniformityNormalized,wavelet-HLH_glszm_GrayLevelVariance,wavelet-HLL_gldm_LargeDependenceHighGrayLevelEmphasis,wavelet-HLL_glcm_Autocorrelation,wavelet-HLL_gldm_HighGrayLevelEmphasis,wavelet-HLL_ngtdm_Complexity,wavelet-HLL_glrlm_HighGrayLevelRunEmphasis,wavelet-HHL_glszm_HighGrayLevelZoneEmphasis,log-sigma-5-0-mm-3D_ngtdm_Contrast,log-sigma-2-0-mm-3D_ngtdm_Contrast,wavelet-HHL_glrlm_LongRunHighGrayLevelEmphasis,log-sigma-2-0-mm-3D_glcm_ClusterShade,log-sigma-5-0-mm-3D_gldm_LowGrayLevelEmphasis,wavelet-HHH_glcm_Correlation,wavelet-LHH_glszm_SmallAreaHighGrayLevelEmphasis,wavelet-HHL_glcm_Autocorrelation,wavelet-HHL_glrlm_HighGrayLevelRunEmphasis,wavelet-HHL_gldm_HighGrayLevelEmphasis,wavelet-LLL_glcm_Contrast,wavelet-HHL_gldm_LargeDependenceHighGrayLevelEmphasis,wavelet-LHH_glcm_Autocorrelation,log-sigma-5-0-mm-3D_gldm_LargeDependenceLowGrayLevelEmphasis,wavelet-LHH_gldm_HighGrayLevelEmphasis,log-sigma-2-0-mm-3D_glcm_ClusterTendency,log-sigma-4-0-mm-3D_firstorder_10Percentile,wavelet-LHH_glrlm_HighGrayLevelRunEmphasis,wavelet-HLL_firstorder_RootMeanSquared,log-sigma-5-0-mm-3D_firstorder_10Percentile,wavelet-HHL_glrlm_ShortRunHighGrayLevelEmphasis,log-sigma-2-0-mm-3D_firstorder_Entropy,wavelet-HHL_glcm_Imc1,wavelet-HLL_gldm_SmallDependenceHighGrayLevelEmphasis,wavelet-LHH_glcm_Imc1,wavelet-HLL_glszm_HighGrayLevelZoneEmphasis,wavelet-HLL_firstorder_InterquartileRange,wavelet-LLH_gldm_DependenceEntropy,log-sigma-2-0-mm-3D_glcm_SumSquares,wavelet-LHH_glszm_HighGrayLevelZoneEmphasis,log-sigma-5-0-mm-3D_glszm_ZonePercentage,wavelet-HLL_firstorder_Mean,wavelet-HLH_glrlm_RunEntropy,log-sigma-3-0-mm-3D_glrlm_LongRunEmphasis,log-sigma-2-0-mm-3D_glszm_GrayLevelNonUniformity,wavelet-LHL_glcm_Imc1,log-sigma-2-0-mm-3D_glrlm_ShortRunEmphasis,wavelet-LHH_glrlm_ShortRunHighGrayLevelEmphasis,wavelet-LLL_glcm_DifferenceVariance,log-sigma-4-0-mm-3D_glcm_Imc1,original_glcm_DifferenceEntropy,original_shape_Maximum2DDiameterColumn,wavelet-HLH_gldm_SmallDependenceHighGrayLevelEmphasis,wavelet-HHH_glrlm_RunLengthNonUniformityNormalized,wavelet-HHL_glcm_DifferenceVariance,log-sigma-1-0-mm-3D_firstorder_Range,log-sigma-1-0-mm-3D_glszm_ZonePercentage,wavelet-HHL_glcm_Imc2,log-sigma-5-0-mm-3D_firstorder_InterquartileRange,log-sigma-2-0-mm-3D_ngtdm_Complexity,wavelet-LLH_gldm_DependenceVariance,wavelet-LLH_gldm_DependenceNonUniformityNormalized,wavelet-LLL_glcm_MaximumProbability,wavelet-LHH_ngtdm_Complexity,log-sigma-3-0-mm-3D_glrlm_RunEntropy,log-sigma-2-0-mm-3D_firstorder_Minimum,log-sigma-3-0-mm-3D_gldm_DependenceVariance,log-sigma-2-0-mm-3D_glcm_JointEntropy,original_shape_Maximum3DDiameter,wavelet-HHL_glszm_ZoneVariance,wavelet-HHH_glcm_ClusterTendency,log-sigma-4-0-mm-3D_glcm_Idmn,wavelet-LLL_firstorder_Maximum,original_shape_MajorAxisLength,log-sigma-5-0-mm-3D_glrlm_LongRunHighGrayLevelEmphasis,original_glcm_JointEnergy,wavelet-HHL_firstorder_Median,wavelet-LLH_firstorder_Median,wavelet-LLH_glcm_Imc2,wavelet-LLH_glszm_GrayLevelVariance,log-sigma-2-0-mm-3D_firstorder_Kurtosis,wavelet-LHH_glcm_Imc2,log-sigma-2-0-mm-3D_gldm_GrayLevelVariance,original_shape_SurfaceVolumeRatio,log-sigma-5-0-mm-3D_glrlm_RunEntropy,wavelet-HHH_glcm_Imc1,wavelet-LLL_glcm_ClusterTendency,log-sigma-4-0-mm-3D_firstorder_RobustMeanAbsoluteDeviation,wavelet-HHL_gldm_SmallDependenceHighGrayLevelEmphasis,wavelet-LLL_glcm_SumSquares,log-sigma-5-0-mm-3D_gldm_DependenceNonUniformityNormalized,log-sigma-2-0-mm-3D_firstorder_Variance,wavelet-HHH_glrlm_RunEntropy,log-sigma-3-0-mm-3D_firstorder_MeanAbsoluteDeviation,log-sigma-2-0-mm-3D_firstorder_MeanAbsoluteDeviation,wavelet-HHH_gldm_GrayLevelVariance,log-sigma-3-0-mm-3D_firstorder_Kurtosis,wavelet-HLL_firstorder_MeanAbsoluteDeviation,log-sigma-2-0-mm-3D_firstorder_10Percentile,log-sigma-4-0-mm-3D_glrlm_RunEntropy,wavelet-HHL_glcm_ClusterTendency,wavelet-LLL_glcm_InverseVariance,log-sigma-2-0-mm-3D_glcm_JointAverage,wavelet-HHH_glrlm_RunPercentage,wavelet-LHH_glrlm_RunEntropy,wavelet-HLL_glcm_ClusterTendency,log-sigma-1-0-mm-3D_ngtdm_Strength,wavelet-HHH_gldm_LargeDependenceEmphasis,wavelet-HHL_glszm_GrayLevelVariance,wavelet-HLL_glszm_GrayLevelVariance,wavelet-HLL_glcm_SumSquares,log-sigma-3-0-mm-3D_firstorder_10Percentile,wavelet-HLL_glcm_JointEntropy,log-sigma-5-0-mm-3D_glcm_Contrast,log-sigma-3-0-mm-3D_glszm_ZonePercentage,wavelet-HLL_glcm_SumEntropy,log-sigma-4-0-mm-3D_glcm_MaximumProbability,wavelet-HLL_glcm_Contrast,wavelet-HHH_glcm_Imc2,wavelet-HHH_glcm_DifferenceVariance,original_glcm_ClusterTendency,wavelet-LLH_glcm_Imc1,wavelet-HHH_glcm_SumSquares,log-sigma-3-0-mm-3D_gldm_GrayLevelVariance,original_glszm_SizeZoneNonUniformity,wavelet-HLH_glcm_Correlation,wavelet-HHH_glcm_ClusterProminence,log-sigma-3-0-mm-3D_firstorder_Variance,original_glcm_SumSquares,log-sigma-4-0-mm-3D_glrlm_LongRunEmphasis,log-sigma-2-0-mm-3D_firstorder_RobustMeanAbsoluteDeviation,log-sigma-3-0-mm-3D_glcm_ClusterTendency,log-sigma-5-0-mm-3D_gldm_DependenceVariance,log-sigma-2-0-mm-3D_firstorder_Mean,original_glcm_Contrast,log-sigma-2-0-mm-3D_firstorder_RootMeanSquared,wavelet-HLH_glcm_MaximumProbability,wavelet-HLL_glcm_DifferenceAverage,wavelet-HHH_firstorder_Uniformity,log-sigma-3-0-mm-3D_glcm_SumSquares,wavelet-HHL_glcm_SumSquares,log-sigma-1-0-mm-3D_firstorder_Minimum,wavelet-HLH_glcm_ClusterShade,wavelet-LHH_glrlm_GrayLevelVariance,log-sigma-2-0-mm-3D_glszm_HighGrayLevelZoneEmphasis,wavelet-HLL_firstorder_Entropy,wavelet-LLH_glcm_SumEntropy,wavelet-HLL_glcm_DifferenceEntropy,wavelet-LHL_gldm_DependenceVariance,log-sigma-4-0-mm-3D_ngtdm_Contrast,wavelet-LHL_glcm_SumEntropy,wavelet-HHH_glrlm_GrayLevelVariance,log-sigma-1-0-mm-3D_firstorder_Median,wavelet-LHH_glszm_GrayLevelVariance,original_glcm_MaximumProbability,log-sigma-2-0-mm-3D_firstorder_InterquartileRange,wavelet-LHL_glrlm_GrayLevelNonUniformityNormalized,original_glcm_DifferenceVariance,log-sigma-1-0-mm-3D_glszm_GrayLevelVariance,log-sigma-2-0-mm-3D_glcm_Idn,wavelet-HHH_glrlm_RunVariance,log-sigma-2-0-mm-3D_glcm_DifferenceEntropy,original_shape_Maximum2DDiameterRow,wavelet-LHL_glcm_InverseVariance,log-sigma-1-0-mm-3D_glcm_JointAverage,log-sigma-5-0-mm-3D_ngtdm_Busyness,log-sigma-1-0-mm-3D_gldm_DependenceNonUniformityNormalized,wavelet-HLL_glcm_DifferenceVariance,wavelet-LLH_glcm_ClusterProminence,wavelet-HHH_glcm_Contrast,wavelet-LLH_glcm_ClusterTendency,log-sigma-5-0-mm-3D_glcm_MaximumProbability,wavelet-LHH_gldm_GrayLevelVariance,wavelet-LHH_gldm_SmallDependenceHighGrayLevelEmphasis,wavelet-HHH_glcm_SumEntropy,original_glcm_DifferenceAverage,log-sigma-3-0-mm-3D_glcm_InverseVariance,wavelet-LLL_ngtdm_Contrast,wavelet-HLH_glcm_Imc2,wavelet-HHH_glcm_JointEnergy,log-sigma-4-0-mm-3D_gldm_DependenceVariance,wavelet-LHL_firstorder_Entropy,wavelet-LHH_ngtdm_Strength,wavelet-LLH_firstorder_10Percentile,wavelet-HLH_glcm_Imc1,wavelet-LLH_glcm_ClusterShade,log-sigma-1-0-mm-3D_gldm_DependenceVariance,wavelet-LLL_glszm_ZonePercentage,log-sigma-4-0-mm-3D_glrlm_RunLengthNonUniformityNormalized,wavelet-HLL_glcm_Correlation,wavelet-HHL_glszm_LargeAreaEmphasis,log-sigma-3-0-mm-3D_glcm_Idn,wavelet-HHL_ngtdm_Complexity,wavelet-LLH_firstorder_RootMeanSquared,log-sigma-3-0-mm-3D_glrlm_RunLengthNonUniformity,wavelet-HHH_firstorder_Entropy,wavelet-LHL_firstorder_Variance,log-sigma-1-0-mm-3D_glcm_MaximumProbability,log-sigma-5-0-mm-3D_glrlm_RunLengthNonUniformityNormalized,wavelet-LLH_ngtdm_Strength,wavelet-HHH_glrlm_LongRunEmphasis,wavelet-LHL_firstorder_10Percentile,wavelet-HHL_glcm_Contrast,wavelet-HHH_glrlm_ShortRunEmphasis,wavelet-LLL_gldm_SmallDependenceEmphasis,wavelet-LLH_firstorder_Entropy,log-sigma-1-0-mm-3D_glrlm_ShortRunEmphasis,wavelet-LHL_gldm_DependenceNonUniformityNormalized,log-sigma-4-0-mm-3D_firstorder_InterquartileRange,wavelet-LHL_gldm_GrayLevelVariance,wavelet-LLH_glcm_JointEntropy,wavelet-LHH_glcm_ClusterTendency,wavelet-LLH_firstorder_Mean,wavelet-HLL_firstorder_Variance,log-sigma-3-0-mm-3D_glrlm_RunLengthNonUniformityNormalized,original_shape_Maximum2DDiameterSlice,wavelet-HLL_glcm_ClusterShade,log-sigma-3-0-mm-3D_ngtdm_Contrast,wavelet-LLH_firstorder_90Percentile,wavelet-HLL_gldm_GrayLevelVariance,log-sigma-4-0-mm-3D_glcm_InverseVariance,wavelet-LLH_glrlm_GrayLevelNonUniformityNormalized,log-sigma-4-0-mm-3D_glrlm_RunLengthNonUniformity,wavelet-HHL_gldm_DependenceVariance,wavelet-HHH_glrlm_GrayLevelNonUniformityNormalized,wavelet-HLL_glrlm_GrayLevelNonUniformityNormalized,wavelet-LLH_glcm_SumSquares,log-sigma-5-0-mm-3D_glszm_LargeAreaLowGrayLevelEmphasis,wavelet-HHH_glcm_JointEntropy,log-sigma-5-0-mm-3D_glcm_Idn,wavelet-HLL_glcm_Imc1,log-sigma-2-0-mm-3D_glrlm_RunLengthNonUniformity,wavelet-HHH_glcm_InverseVariance,wavelet-LLL_glcm_Id,wavelet-LHH_firstorder_Variance,log-sigma-5-0-mm-3D_glcm_Imc1,log-sigma-1-0-mm-3D_glrlm_RunLengthNonUniformityNormalized,wavelet-HLH_firstorder_Mean,wavelet-HLH_glcm_DifferenceVariance,log-sigma-2-0-mm-3D_glcm_Id,log-sigma-2-0-mm-3D_glcm_ClusterProminence,log-sigma-5-0-mm-3D_glcm_InverseVariance,wavelet-LLH_glcm_DifferenceEntropy,log-sigma-2-0-mm-3D_glcm_Idm,wavelet-LHL_glszm_ZonePercentage,wavelet-HLL_glcm_InverseVariance,wavelet-LLH_glszm_LargeAreaLowGrayLevelEmphasis,wavelet-LLL_glcm_Idm,wavelet-HHL_glcm_DifferenceEntropy,wavelet-HHH_glcm_DifferenceAverage,wavelet-HLH_glcm_Contrast,log-sigma-4-0-mm-3D_glcm_Contrast,wavelet-HHH_glcm_DifferenceEntropy,log-sigma-2-0-mm-3D_gldm_SmallDependenceHighGrayLevelEmphasis,log-sigma-2-0-mm-3D_glcm_DifferenceAverage,wavelet-HLL_gldm_SmallDependenceEmphasis,wavelet-HLL_glcm_Imc2,wavelet-LHL_firstorder_Uniformity,wavelet-LLL_glszm_ZoneVariance,wavelet-LLL_glszm_LargeAreaEmphasis,original_shape_MinorAxisLength,wavelet-LHL_glrlm_GrayLevelVariance,wavelet-LHH_glcm_DifferenceVariance,log-sigma-5-0-mm-3D_glcm_DifferenceAverage,wavelet-LHL_firstorder_MeanAbsoluteDeviation,wavelet-LHL_glcm_JointEntropy,original_ngtdm_Strength,wavelet-HLL_firstorder_Uniformity,wavelet-LHL_glcm_ClusterTendency,log-sigma-2-0-mm-3D_glcm_InverseVariance,log-sigma-3-0-mm-3D_gldm_SmallDependenceEmphasis,wavelet-HHL_gldm_DependenceEntropy,log-sigma-5-0-mm-3D_glrlm_RunLengthNonUniformity,log-sigma-1-0-mm-3D_glrlm_RunVariance,log-sigma-2-0-mm-3D_gldm_LargeDependenceHighGrayLevelEmphasis,log-sigma-3-0-mm-3D_firstorder_RootMeanSquared,log-sigma-3-0-mm-3D_firstorder_Median,wavelet-HLH_firstorder_RootMeanSquared,wavelet-HLL_glcm_Id,log-sigma-3-0-mm-3D_firstorder_Mean,wavelet-LLH_firstorder_MeanAbsoluteDeviation,wavelet-HLL_glcm_ClusterProminence,wavelet-LHL_glrlm_RunVariance,log-sigma-2-0-mm-3D_glcm_Contrast,log-sigma-3-0-mm-3D_glszm_LargeAreaEmphasis,wavelet-HLL_glcm_Idm,log-sigma-3-0-mm-3D_glszm_ZoneVariance,wavelet-LHH_glcm_SumSquares,log-sigma-5-0-mm-3D_firstorder_RootMeanSquared,wavelet-HHH_gldm_SmallDependenceHighGrayLevelEmphasis,wavelet-LHL_glcm_DifferenceEntropy,wavelet-LLH_firstorder_Uniformity,log-sigma-5-0-mm-3D_firstorder_Mean,wavelet-HHH_glcm_Idm,log-sigma-2-0-mm-3D_glrlm_LongRunHighGrayLevelEmphasis,wavelet-HLH_glcm_SumSquares,wavelet-LLL_glrlm_ShortRunEmphasis,log-sigma-4-0-mm-3D_firstorder_RootMeanSquared,wavelet-HHH_glcm_Id,log-sigma-4-0-mm-3D_firstorder_Mean,log-sigma-2-0-mm-3D_glcm_DifferenceVariance,wavelet-HHL_firstorder_Entropy,wavelet-HHL_glrlm_RunVariance,wavelet-LLH_gldm_GrayLevelVariance,wavelet-HLL_glrlm_GrayLevelVariance,log-sigma-5-0-mm-3D_glcm_Idm,wavelet-LLH_glcm_MaximumProbability,wavelet-LLH_glcm_DifferenceAverage,wavelet-LLH_firstorder_Variance,log-sigma-5-0-mm-3D_gldm_SmallDependenceEmphasis,original_glrlm_ShortRunEmphasis,log-sigma-4-0-mm-3D_glcm_Idn,log-sigma-5-0-mm-3D_glcm_Id,wavelet-LHL_ngtdm_Complexity,wavelet-LHL_glcm_JointEnergy,wavelet-LLL_glrlm_LongRunEmphasis,wavelet-LHL_glcm_SumSquares,wavelet-LHH_glrlm_RunVariance,wavelet-HHH_gldm_DependenceEntropy,wavelet-LHL_glszm_LargeAreaHighGrayLevelEmphasis,wavelet-HLL_glcm_JointEnergy,log-sigma-1-0-mm-3D_gldm_SmallDependenceEmphasis,wavelet-LLH_glrlm_GrayLevelVariance,wavelet-HLH_glrlm_GrayLevelVariance,wavelet-HHH_gldm_DependenceVariance,log-sigma-3-0-mm-3D_glcm_Contrast,wavelet-HLH_gldm_GrayLevelVariance,wavelet-HLH_glcm_ClusterTendency,log-sigma-2-0-mm-3D_glrlm_RunLengthNonUniformityNormalized,wavelet-LHH_glcm_InverseVariance,wavelet-LHL_glszm_SizeZoneNonUniformity,wavelet-HLL_glszm_SizeZoneNonUniformity,log-sigma-3-0-mm-3D_glcm_Id,wavelet-LLH_glcm_JointEnergy,wavelet-HLL_glrlm_RunVariance,wavelet-LHL_glrlm_LongRunHighGrayLevelEmphasis,wavelet-LHL_gldm_SmallDependenceEmphasis,wavelet-HHL_glcm_SumEntropy,wavelet-LLH_glcm_Idm,wavelet-HLH_glcm_SumEntropy,wavelet-HLH_glcm_InverseVariance,wavelet-HLL_gldm_DependenceNonUniformityNormalized,wavelet-HLL_glcm_MaximumProbability,log-sigma-3-0-mm-3D_glcm_Idm,log-sigma-1-0-mm-3D_ngtdm_Complexity,log-sigma-4-0-mm-3D_glcm_Id,wavelet-HHL_firstorder_10Percentile,wavelet-LLH_glcm_Id,original_glcm_InverseVariance,wavelet-HLH_firstorder_Entropy,wavelet-HHL_firstorder_MeanAbsoluteDeviation,wavelet-HLH_glcm_DifferenceAverage,log-sigma-1-0-mm-3D_gldm_SmallDependenceHighGrayLevelEmphasis,wavelet-LHL_firstorder_Median,log-sigma-4-0-mm-3D_glcm_DifferenceAverage,wavelet-LLL_glrlm_RunLengthNonUniformityNormalized,wavelet-HLL_glszm_ZonePercentage,log-sigma-2-0-mm-3D_gldm_HighGrayLevelEmphasis,wavelet-LHH_ngtdm_Busyness,wavelet-LHH_glcm_ClusterProminence,log-sigma-2-0-mm-3D_glcm_Autocorrelation,wavelet-HLH_firstorder_Variance,log-sigma-1-0-mm-3D_glcm_InverseVariance,log-sigma-4-0-mm-3D_glcm_Idm,wavelet-LHH_glcm_Contrast,log-sigma-2-0-mm-3D_glszm_LargeAreaHighGrayLevelEmphasis,log-sigma-3-0-mm-3D_glcm_DifferenceAverage,original_glcm_Idm,wavelet-HLL_glrlm_LongRunEmphasis,wavelet-HLH_glcm_JointEntropy,wavelet-LHL_gldm_LargeDependenceHighGrayLevelEmphasis,original_glrlm_RunLengthNonUniformityNormalized,wavelet-HLL_gldm_DependenceVariance,wavelet-HHL_glrlm_GrayLevelNonUniformityNormalized,wavelet-HLH_firstorder_Uniformity,original_glcm_Id,wavelet-HLH_glcm_DifferenceEntropy,wavelet-HHL_glrlm_LongRunEmphasis,wavelet-LHL_firstorder_90Percentile,log-sigma-2-0-mm-3D_gldm_SmallDependenceEmphasis,wavelet-LLH_glcm_Contrast,wavelet-LLL_gldm_LargeDependenceEmphasis,wavelet-HHL_glcm_JointEntropy,log-sigma-1-0-mm-3D_glrlm_RunPercentage,wavelet-LHL_glcm_ClusterShade,wavelet-HLH_gldm_DependenceEntropy,original_glszm_LargeAreaEmphasis,original_glszm_ZoneVariance,wavelet-HLH_glrlm_GrayLevelNonUniformityNormalized,log-sigma-3-0-mm-3D_firstorder_RobustMeanAbsoluteDeviation,log-sigma-5-0-mm-3D_glrlm_LongRunEmphasis,log-sigma-1-0-mm-3D_glrlm_LongRunEmphasis,log-sigma-2-0-mm-3D_glrlm_ShortRunHighGrayLevelEmphasis,log-sigma-2-0-mm-3D_gldm_LargeDependenceEmphasis,wavelet-LHL_glcm_Autocorrelation,wavelet-LHL_gldm_HighGrayLevelEmphasis,wavelet-LHL_glrlm_HighGrayLevelRunEmphasis,log-sigma-4-0-mm-3D_gldm_SmallDependenceEmphasis,log-sigma-2-0-mm-3D_glrlm_HighGrayLevelRunEmphasis,wavelet-LLH_firstorder_RobustMeanAbsoluteDeviation,wavelet-LHL_glrlm_LongRunEmphasis,log-sigma-2-0-mm-3D_glrlm_RunPercentage,wavelet-HHL_gldm_DependenceNonUniformityNormalized,wavelet-HLL_glrlm_ShortRunEmphasis,wavelet-LLL_glrlm_RunPercentage,wavelet-LLH_glszm_SizeZoneNonUniformity,wavelet-LHL_glcm_DifferenceAverage,wavelet-LHL_glcm_MaximumProbability,log-sigma-3-0-mm-3D_glrlm_RunPercentage,wavelet-LHL_glszm_HighGrayLevelZoneEmphasis,log-sigma-2-0-mm-3D_glszm_ZoneVariance,log-sigma-2-0-mm-3D_glszm_LargeAreaEmphasis,wavelet-LHL_glrlm_ShortRunHighGrayLevelEmphasis,log-sigma-3-0-mm-3D_gldm_LargeDependenceEmphasis,wavelet-HLH_glcm_JointEnergy,log-sigma-2-0-mm-3D_glrlm_LongRunEmphasis,wavelet-HHL_glcm_DifferenceAverage,log-sigma-1-0-mm-3D_gldm_LargeDependenceEmphasis,wavelet-LLL_glrlm_RunVariance,log-sigma-4-0-mm-3D_gldm_LargeDependenceEmphasis,wavelet-LLH_glcm_DifferenceVariance,wavelet-HLL_glrlm_RunLengthNonUniformityNormalized,wavelet-LHL_glcm_Contrast,wavelet-LHL_glcm_Id,wavelet-HLH_gldm_LargeDependenceEmphasis,wavelet-HHL_glszm_LargeAreaHighGrayLevelEmphasis,log-sigma-4-0-mm-3D_glrlm_RunPercentage,wavelet-HHL_glcm_ClusterProminence,wavelet-LHH_gldm_DependenceEntropy,wavelet-LLH_firstorder_InterquartileRange,wavelet-LHL_glcm_Idm,wavelet-HLH_glrlm_RunVariance,wavelet-HLL_glrlm_RunPercentage,wavelet-HLL_gldm_LargeDependenceEmphasis,wavelet-LHH_glrlm_LongRunEmphasis,wavelet-HLH_glrlm_RunLengthNonUniformityNormalized,log-sigma-5-0-mm-3D_glrlm_RunPercentage,wavelet-HLH_glrlm_RunPercentage,original_glrlm_LongRunEmphasis,wavelet-LHL_glszm_ZoneVariance,wavelet-LHL_glszm_GrayLevelVariance,wavelet-LHL_glszm_LargeAreaEmphasis,log-sigma-5-0-mm-3D_gldm_LargeDependenceEmphasis,wavelet-HHL_firstorder_Uniformity,wavelet-HLH_glcm_Idm,wavelet-LHH_gldm_LargeDependenceEmphasis,wavelet-HHH_gldm_DependenceNonUniformityNormalized,wavelet-HLL_glszm_ZoneVariance,wavelet-LHL_gldm_LargeDependenceEmphasis,wavelet-HLH_gldm_DependenceNonUniformityNormalized,wavelet-HLL_glszm_LargeAreaEmphasis,wavelet-LHL_glszm_SmallAreaHighGrayLevelEmphasis,wavelet-HLH_glcm_Id,wavelet-HHH_firstorder_Variance,log-sigma-1-0-mm-3D_glcm_ClusterProminence,log-sigma-1-0-mm-3D_glszm_SmallAreaHighGrayLevelEmphasis,wavelet-HHL_gldm_LargeDependenceEmphasis,wavelet-HLH_gldm_DependenceVariance,log-sigma-5-0-mm-3D_firstorder_Median,log-sigma-5-0-mm-3D_glszm_LargeAreaHighGrayLevelEmphasis,wavelet-LHH_glcm_SumEntropy,wavelet-LHH_gldm_DependenceVariance,wavelet-LLH_glcm_InverseVariance,wavelet-LHL_glcm_DifferenceVariance,wavelet-LHH_firstorder_Entropy,original_shape_SurfaceArea,wavelet-HHL_glszm_ZonePercentage,wavelet-HHL_firstorder_90Percentile,log-sigma-4-0-mm-3D_firstorder_Median,wavelet-LHL_glrlm_ShortRunEmphasis,wavelet-LLL_gldm_DependenceNonUniformityNormalized,wavelet-LLL_gldm_DependenceVariance,wavelet-LHH_gldm_DependenceNonUniformityNormalized,log-sigma-4-0-mm-3D_glszm_LargeAreaEmphasis,log-sigma-4-0-mm-3D_glszm_ZoneVariance,wavelet-LHH_glcm_DifferenceEntropy,wavelet-LHL_glrlm_RunLengthNonUniformityNormalized,wavelet-HHL_glrlm_RunPercentage,wavelet-HLH_glszm_ZonePercentage,wavelet-LHH_glrlm_RunPercentage,wavelet-LLH_glszm_ZonePercentage,wavelet-LHL_firstorder_RobustMeanAbsoluteDeviation,wavelet-LHL_glrlm_RunPercentage,wavelet-HHL_firstorder_InterquartileRange,wavelet-HLH_glrlm_LongRunEmphasis,wavelet-HHL_gldm_SmallDependenceEmphasis,log-sigma-3-0-mm-3D_firstorder_InterquartileRange,wavelet-HHL_glcm_JointEnergy,wavelet-HLH_firstorder_MeanAbsoluteDeviation,wavelet-LHL_gldm_SmallDependenceHighGrayLevelEmphasis,wavelet-LHL_firstorder_InterquartileRange,original_gldm_DependenceNonUniformityNormalized,log-sigma-1-0-mm-3D_glszm_ZoneVariance,original_glrlm_RunVariance,wavelet-HHH_glszm_LargeAreaHighGrayLevelEmphasis,log-sigma-1-0-mm-3D_glszm_LargeAreaEmphasis,log-sigma-1-0-mm-3D_glrlm_RunLengthNonUniformity,wavelet-LLH_gldm_SmallDependenceEmphasis,wavelet-LHH_glcm_JointEntropy,wavelet-HHL_glcm_MaximumProbability,wavelet-LLH_glrlm_RunLengthNonUniformityNormalized,wavelet-HHL_glcm_Idm,log-sigma-1-0-mm-3D_glszm_HighGrayLevelZoneEmphasis,wavelet-HHL_firstorder_RobustMeanAbsoluteDeviation,wavelet-LLL_glcm_ClusterProminence,log-sigma-1-0-mm-3D_glrlm_ShortRunHighGrayLevelEmphasis,wavelet-HLL_firstorder_90Percentile,wavelet-LHH_glcm_DifferenceAverage,wavelet-HHL_glcm_Id,log-sigma-1-0-mm-3D_glrlm_LongRunHighGrayLevelEmphasis,log-sigma-3-0-mm-3D_glrlm_RunVariance,wavelet-LHH_firstorder_MeanAbsoluteDeviation,wavelet-HLH_glrlm_ShortRunEmphasis,log-sigma-1-0-mm-3D_glcm_Autocorrelation,wavelet-LHH_glrlm_GrayLevelNonUniformityNormalized,log-sigma-1-0-mm-3D_gldm_HighGrayLevelEmphasis,wavelet-LLH_glrlm_ShortRunEmphasis,wavelet-HHL_glrlm_RunLengthNonUniformityNormalized,wavelet-HLH_gldm_SmallDependenceEmphasis,log-sigma-1-0-mm-3D_gldm_LargeDependenceHighGrayLevelEmphasis,wavelet-HHL_glrlm_ShortRunEmphasis,original_glcm_ClusterProminence,wavelet-LHH_glcm_MaximumProbability,original_glrlm_RunPercentage,log-sigma-1-0-mm-3D_glrlm_HighGrayLevelRunEmphasis,wavelet-LLL_glcm_ClusterShade,wavelet-LHH_glszm_LargeAreaHighGrayLevelEmphasis,wavelet-LHH_glszm_LargeAreaEmphasis,original_glcm_ClusterShade,wavelet-HLH_firstorder_10Percentile,wavelet-LHH_firstorder_90Percentile,wavelet-LHH_firstorder_Uniformity,wavelet-HLH_firstorder_90Percentile,original_gldm_LargeDependenceEmphasis,wavelet-HHH_firstorder_10Percentile,wavelet-LHH_firstorder_10Percentile,log-sigma-1-0-mm-3D_glszm_GrayLevelNonUniformity,log-sigma-2-0-mm-3D_glrlm_RunVariance,wavelet-LHH_glrlm_RunLengthNonUniformityNormalized,wavelet-LHL_ngtdm_Strength,wavelet-LLH_glrlm_RunPercentage,wavelet-LHH_glszm_ZonePercentage,wavelet-LHH_glrlm_ShortRunEmphasis,wavelet-LLH_glszm_LargeAreaHighGrayLevelEmphasis,wavelet-LLL_firstorder_10Percentile,wavelet-HLH_glszm_SizeZoneNonUniformity,wavelet-HHH_firstorder_MeanAbsoluteDeviation,original_firstorder_10Percentile,wavelet-HLH_firstorder_InterquartileRange,wavelet-LHH_glcm_JointEnergy,wavelet-LLH_glrlm_RunLengthNonUniformity,wavelet-LHH_glszm_ZoneVariance,wavelet-LLH_gldm_LargeDependenceEmphasis,log-sigma-5-0-mm-3D_glrlm_GrayLevelNonUniformity,original_gldm_DependenceVariance,wavelet-LLH_glrlm_LongRunEmphasis,wavelet-LHH_glcm_Id,wavelet-LHH_glcm_Idm,wavelet-LLH_glrlm_RunVariance,log-sigma-4-0-mm-3D_glrlm_RunVariance,wavelet-HHH_firstorder_90Percentile,wavelet-HHH_glszm_ZonePercentage,wavelet-HLH_firstorder_RobustMeanAbsoluteDeviation,wavelet-LHL_gldm_GrayLevelNonUniformity,wavelet-LLH_glszm_ZoneVariance,wavelet-LLH_glszm_LargeAreaEmphasis,wavelet-HHH_firstorder_InterquartileRange,wavelet-LLL_glszm_GrayLevelNonUniformity,wavelet-HLH_glszm_LargeAreaHighGrayLevelEmphasis,wavelet-LHH_gldm_SmallDependenceEmphasis,original_glszm_GrayLevelNonUniformity,wavelet-LLL_firstorder_Energy,wavelet-LLL_firstorder_TotalEnergy,wavelet-HLL_gldm_GrayLevelNonUniformity,wavelet-LLH_glszm_GrayLevelNonUniformity,log-sigma-4-0-mm-3D_glrlm_GrayLevelNonUniformity,wavelet-LHH_firstorder_RobustMeanAbsoluteDeviation,wavelet-HLH_glrlm_RunLengthNonUniformity,wavelet-LLL_glrlm_RunLengthNonUniformity,wavelet-HHH_firstorder_RobustMeanAbsoluteDeviation,wavelet-LHH_glszm_SizeZoneNonUniformity,original_firstorder_Energy,original_firstorder_TotalEnergy,wavelet-LHH_glrlm_RunLengthNonUniformity,log-sigma-5-0-mm-3D_glszm_LargeAreaEmphasis,original_glrlm_RunLengthNonUniformity,log-sigma-5-0-mm-3D_glszm_ZoneVariance,wavelet-HHL_gldm_GrayLevelNonUniformity,wavelet-HHL_glrlm_GrayLevelNonUniformity,wavelet-HHH_gldm_SmallDependenceEmphasis,wavelet-HLL_glrlm_GrayLevelNonUniformity,wavelet-HHL_gldm_DependenceNonUniformity,wavelet-LHL_glrlm_GrayLevelNonUniformity,wavelet-HHH_glrlm_RunLengthNonUniformity,wavelet-LHH_firstorder_InterquartileRange,wavelet-HHH_glrlm_GrayLevelNonUniformity,wavelet-LHH_firstorder_Energy,wavelet-LHH_firstorder_TotalEnergy,original_shape_VoxelVolume,original_shape_MeshVolume,wavelet-LHL_firstorder_Energy,wavelet-LHL_firstorder_TotalEnergy,wavelet-HHL_firstorder_Energy,wavelet-HHL_firstorder_TotalEnergy,wavelet-HLH_firstorder_Energy,wavelet-HLH_firstorder_TotalEnergy,wavelet-HHH_firstorder_Energy,wavelet-HHH_firstorder_TotalEnergy,wavelet-HLL_gldm_DependenceNonUniformity,wavelet-HLL_firstorder_Energy,wavelet-HLL_firstorder_TotalEnergy,wavelet-HHH_gldm_GrayLevelNonUniformity,wavelet-LLH_firstorder_Energy,wavelet-LLH_firstorder_TotalEnergy,log-sigma-1-0-mm-3D_firstorder_Energy,log-sigma-1-0-mm-3D_firstorder_TotalEnergy,wavelet-LLH_glrlm_GrayLevelNonUniformity,log-sigma-2-0-mm-3D_firstorder_Energy,log-sigma-2-0-mm-3D_firstorder_TotalEnergy,wavelet-HLH_glrlm_GrayLevelNonUniformity,wavelet-LHH_glrlm_GrayLevelNonUniformity,wavelet-LHL_gldm_DependenceNonUniformity,log-sigma-5-0-mm-3D_firstorder_Energy,log-sigma-5-0-mm-3D_firstorder_TotalEnergy,log-sigma-4-0-mm-3D_firstorder_Energy,log-sigma-4-0-mm-3D_firstorder_TotalEnergy,log-sigma-3-0-mm-3D_firstorder_Energy,log-sigma-3-0-mm-3D_firstorder_TotalEnergy,wavelet-LHH_gldm_GrayLevelNonUniformity,wavelet-HLH_gldm_GrayLevelNonUniformity,wavelet-HLL_glrlm_RunLengthNonUniformity,wavelet-HHL_glrlm_RunLengthNonUniformity,wavelet-LHL_glrlm_RunLengthNonUniformity,wavelet-HLH_glszm_GrayLevelNonUniformity,original_shape_LeastAxisLength,log-sigma-5-0-mm-3D_glrlm_RunVariance,wavelet-HHH_gldm_DependenceNonUniformity,wavelet-LHH_gldm_DependenceNonUniformity,wavelet-HLH_gldm_DependenceNonUniformity,wavelet-LHL_glszm_GrayLevelNonUniformity,wavelet-LLH_gldm_GrayLevelNonUniformity,wavelet-HHL_glszm_SizeZoneNonUniformity,original_gldm_GrayLevelNonUniformity,original_gldm_DependenceNonUniformity,log-sigma-1-0-mm-3D_gldm_GrayLevelNonUniformity,original_firstorder_Median,wavelet-LLL_firstorder_Median,wavelet-HHH_glszm_ZoneVariance,wavelet-LLH_gldm_DependenceNonUniformity,log-sigma-1-0-mm-3D_glrlm_GrayLevelNonUniformity,log-sigma-3-0-mm-3D_glrlm_GrayLevelNonUniformity,wavelet-HLL_glszm_GrayLevelNonUniformity,log-sigma-2-0-mm-3D_gldm_DependenceNonUniformity,wavelet-HLH_glszm_LargeAreaEmphasis,wavelet-HLH_glszm_ZoneVariance,wavelet-LLL_firstorder_Mean,original_firstorder_Mean,original_firstorder_RootMeanSquared,wavelet-HHH_glszm_GrayLevelNonUniformity,wavelet-LLL_firstorder_RootMeanSquared,wavelet-LLL_gldm_DependenceNonUniformity,wavelet-HHH_glszm_LargeAreaEmphasis,wavelet-LHH_glszm_GrayLevelNonUniformity,wavelet-LLL_gldm_GrayLevelNonUniformity,wavelet-LHL_glcm_ClusterProminence,wavelet-HHH_glszm_SizeZoneNonUniformity,log-sigma-1-0-mm-3D_gldm_DependenceNonUniformity,original_glrlm_GrayLevelNonUniformity,wavelet-LLL_firstorder_90Percentile,wavelet-LLL_glrlm_GrayLevelNonUniformity,log-sigma-2-0-mm-3D_glrlm_GrayLevelNonUniformity,log-sigma-2-0-mm-3D_gldm_GrayLevelNonUniformity,original_firstorder_90Percentile,log-sigma-3-0-mm-3D_gldm_GrayLevelNonUniformity,log-sigma-3-0-mm-3D_gldm_DependenceNonUniformity,log-sigma-4-0-mm-3D_gldm_GrayLevelNonUniformity,wavelet-HHL_glszm_GrayLevelNonUniformity,log-sigma-5-0-mm-3D_gldm_GrayLevelNonUniformity,log-sigma-4-0-mm-3D_gldm_DependenceNonUniformity,log-sigma-5-0-mm-3D_gldm_DependenceNonUniformity

#### **UECT Features in LASSO**

Total 23 features.

original_shape_SurfaceVolumeRatio,wavelet-LLH_glszm_GrayLevelVariance,wavelet-HHH_glszm_SmallAreaEmphasis,wavelet-LLH_glszm_SmallAreaHighGrayLevelEmphasis,wavelet-HLH_glszm_SmallAreaEmphasis,wavelet-HHH_gldm_LargeDependenceEmphasis,wavelet-LLH_glszm_HighGrayLevelZoneEmphasis,log-sigma-4-0-mm-3D_glrlm_LongRunHighGrayLevelEmphasis,wavelet-LHH_glrlm_RunEntropy,log-sigma-1-0-mm-3D_gldm_LargeDependenceLowGrayLevelEmphasis,log-sigma-5-0-mm-3D_glcm_JointAverage,wavelet-LLH_glszm_SmallAreaEmphasis,wavelet-HLH_glrlm_RunEntropy,log-sigma-5-0-mm-3D_glcm_ClusterTendency,log-sigma-5-0-mm-3D_glcm_Correlation,log-sigma-4-0-mm-3D_glcm_Correlation,log-sigma-5-0-mm-3D_glszm_SmallAreaHighGrayLevelEmphasis,log-sigma-5-0-mm-3D_glrlm_GrayLevelNonUniformityNormalized,log-sigma-5-0-mm-3D_glrlm_RunLengthNonUniformity,wavelet-LLL_firstorder_Median,wavelet-HHH_glrlm_RunVariance,log-sigma-1-0-mm-3D_glcm_JointAverage,log-sigma-4-0-mm-3D_glrlm_RunLengthNonUniformity,

At last, four selected features consisted of one shape feature, one GLSZM-based features, one GLRLM-based features, and one GLCM-based features which were analyzed as follows:

1. shape featuer: SurfaceVolumeRatio. A lower value indicates a more compact (sphere-like) shape. This feature is not dimensionless, and is therefore (partly) dependent on the volume of the ROI.
2. GLSZM-based features: SmallAreaHighGrayLevelEmphasis measures the proportion in the image of the joint distribution of smaller size zones with lower gray-level values.
3. GLRLM-based feature: RunEntropy measures the uncertainty/randomness in the distribution of run lengths and gray levels. A higher value indicates more heterogeneity in the texture patterns.

(4) GLCM-based feature: JointAverage. Returns the mean gray level intensity of the i distribution.

#### **CECT features in LASSO**

Total 134 features.

log-sigma-4-0-mm-3D_glszm_SizeZoneNonUniformity,log-sigma-2-0-mm-3D_gldm_DependenceEntropy,wavelet-LLL_glcm_Imc2,wavelet-LLL_glcm_JointEnergy,log-sigma-4-0-mm-3D_gldm_GrayLevelVariance,log-sigma-4-0-mm-3D_glszm_GrayLevelNonUniformity,log-sigma-1-0-mm-3D_firstorder_Entropy,log-sigma-1-0-mm-3D_glcm_JointEntropy,wavelet-LLL_glrlm_GrayLevelNonUniformityNormalized,log-sigma-2-0-mm-3D_glcm_MaximumProbability,log-sigma-4-0-mm-3D_glcm_ClusterTendency,log-sigma-2-0-mm-3D_glszm_SmallAreaHighGrayLevelEmphasis,wavelet-LLL_glcm_SumEntropy,log-sigma-4-0-mm-3D_glcm_SumSquares,wavelet-LLH_glszm_SmallAreaHighGrayLevelEmphasis,wavelet-HLH_glszm_ZoneEntropy,log-sigma-2-0-mm-3D_glcm_JointEnergy,original_glcm_SumEntropy,wavelet-HHL_firstorder_Range,wavelet-HLL_ngtdm_Contrast,wavelet-LLL_firstorder_Entropy,wavelet-LLH_firstorder_Range,wavelet-HHL_firstorder_Maximum,wavelet-LLH_glrlm_ShortRunHighGrayLevelEmphasis,wavelet-LLL_firstorder_Uniformity,wavelet-LLH_firstorder_Minimum,wavelet-LLH_glrlm_HighGrayLevelRunEmphasis,wavelet-LLH_glcm_JointAverage,wavelet-LLH_gldm_HighGrayLevelEmphasis,wavelet-LLH_glcm_Autocorrelation,original_firstorder_Maximum,wavelet-LLH_glszm_HighGrayLevelZoneEmphasis,log-sigma-3-0-mm-3D_glcm_Idmn,log-sigma-1-0-mm-3D_firstorder_Uniformity,wavelet-LLH_gldm_SmallDependenceHighGrayLevelEmphasis,log-sigma-3-0-mm-3D_firstorder_Entropy,wavelet-LLL_glcm_JointEntropy,wavelet-HHL_glcm_Correlation,log-sigma-5-0-mm-3D_glrlm_LowGrayLevelRunEmphasis,wavelet-LLH_glrlm_LongRunHighGrayLevelEmphasis,log-sigma-2-0-mm-3D_firstorder_Uniformity,wavelet-HLL_firstorder_Minimum,wavelet-HLL_glcm_JointAverage,log-sigma-2-0-mm-3D_glcm_Idmn,log-sigma-2-0-mm-3D_glrlm_ShortRunLowGrayLevelEmphasis,log-sigma-2-0-mm-3D_firstorder_90Percentile,log-sigma-5-0-mm-3D_glszm_SizeZoneNonUniformity,wavelet-HLL_firstorder_Range,wavelet-HHL_glszm_SmallAreaHighGrayLevelEmphasis,log-sigma-2-0-mm-3D_firstorder_Range,wavelet-LHL_ngtdm_Contrast,wavelet-LLH_ngtdm_Complexity,log-sigma-3-0-mm-3D_firstorder_90Percentile,wavelet-HHH_ngtdm_Complexity,wavelet-HLL_glrlm_LongRunHighGrayLevelEmphasis,wavelet-HLL_glszm_SmallAreaHighGrayLevelEmphasis,log-sigma-1-0-mm-3D_glcm_JointEnergy,log-sigma-2-0-mm-3D_glcm_SumEntropy,wavelet-HLL_glrlm_ShortRunHighGrayLevelEmphasis,wavelet-HLH_glcm_ClusterProminence,wavelet-HLL_gldm_LargeDependenceHighGrayLevelEmphasis,wavelet-HLL_glcm_Autocorrelation,wavelet-HLL_gldm_HighGrayLevelEmphasis,wavelet-HLL_ngtdm_Complexity,wavelet-HLL_glrlm_HighGrayLevelRunEmphasis,wavelet-HHL_glszm_HighGrayLevelZoneEmphasis,wavelet-HHL_glrlm_LongRunHighGrayLevelEmphasis,log-sigma-5-0-mm-3D_gldm_LowGrayLevelEmphasis,log-sigma-5-0-mm-3D_gldm_LargeDependenceLowGrayLevelEmphasis,log-sigma-2-0-mm-3D_firstorder_Entropy,wavelet-HLL_gldm_SmallDependenceHighGrayLevelEmphasis,wavelet-HLL_glszm_HighGrayLevelZoneEmphasis,log-sigma-2-0-mm-3D_glszm_GrayLevelNonUniformity,wavelet-HLH_gldm_SmallDependenceHighGrayLevelEmphasis,log-sigma-1-0-mm-3D_firstorder_Range,log-sigma-2-0-mm-3D_ngtdm_Complexity,wavelet-LLL_glcm_MaximumProbability,log-sigma-2-0-mm-3D_glcm_JointEntropy,log-sigma-4-0-mm-3D_glcm_Idmn,wavelet-LLL_firstorder_Maximum,wavelet-LLH_glcm_Imc2,log-sigma-2-0-mm-3D_glcm_JointAverage,wavelet-HLH_glcm_Correlation,log-sigma-2-0-mm-3D_glszm_HighGrayLevelZoneEmphasis,wavelet-LLH_glcm_SumEntropy,log-sigma-1-0-mm-3D_glszm_GrayLevelVariance,original_shape_Maximum2DDiameterRow,wavelet-LLH_glcm_ClusterProminence,wavelet-LLH_glcm_ClusterTendency,wavelet-HLL_glcm_Correlation,wavelet-HHL_ngtdm_Complexity,log-sigma-3-0-mm-3D_glrlm_RunLengthNonUniformity,wavelet-LLH_firstorder_Entropy,wavelet-LLH_glcm_JointEntropy,wavelet-LLH_firstorder_90Percentile,wavelet-LLH_glrlm_GrayLevelNonUniformityNormalized,log-sigma-4-0-mm-3D_glrlm_RunLengthNonUniformity,wavelet-LLH_glcm_SumSquares,log-sigma-2-0-mm-3D_glrlm_RunLengthNonUniformity,wavelet-LLH_glcm_DifferenceEntropy,log-sigma-2-0-mm-3D_gldm_SmallDependenceHighGrayLevelEmphasis,original_shape_MinorAxisLength,log-sigma-5-0-mm-3D_glrlm_RunLengthNonUniformity,wavelet-LLH_firstorder_MeanAbsoluteDeviation,wavelet-HHH_gldm_SmallDependenceHighGrayLevelEmphasis,wavelet-LLH_firstorder_Uniformity,wavelet-LLH_gldm_GrayLevelVariance,wavelet-LLH_glcm_MaximumProbability,wavelet-LLH_glcm_DifferenceAverage,wavelet-LLH_firstorder_Variance,wavelet-LLH_glrlm_GrayLevelVariance,wavelet-LLH_glcm_JointEnergy,wavelet-LLH_glcm_Idm,wavelet-LLH_glcm_Id,log-sigma-2-0-mm-3D_gldm_HighGrayLevelEmphasis,log-sigma-2-0-mm-3D_glcm_Autocorrelation,wavelet-LLH_glcm_Contrast,log-sigma-2-0-mm-3D_glrlm_ShortRunHighGrayLevelEmphasis,log-sigma-2-0-mm-3D_glrlm_HighGrayLevelRunEmphasis,wavelet-LLH_firstorder_RobustMeanAbsoluteDeviation,wavelet-LLH_firstorder_InterquartileRange,wavelet-HLH_glszm_ZonePercentage,log-sigma-1-0-mm-3D_glrlm_RunLengthNonUniformity,wavelet-LLH_gldm_SmallDependenceEmphasis,wavelet-LLH_glrlm_RunLengthNonUniformityNormalized,wavelet-LLH_glrlm_ShortRunEmphasis,wavelet-HLH_gldm_SmallDependenceEmphasis,wavelet-HLH_firstorder_10Percentile,wavelet-HHH_firstorder_10Percentile,log-sigma-1-0-mm-3D_glszm_GrayLevelNonUniformity,wavelet-LLH_glrlm_RunPercentage,wavelet-HLH_glszm_SizeZoneNonUniformity,wavelet-HHH_firstorder_MeanAbsoluteDeviation,wavelet-LLH_gldm_LargeDependenceEmphasis,

1. two GLCM-based features: Idmn and JointAverage. IDMN is a measure of the local homogeneity of an image. IDMN weights are the inverse of the Contrast weights (decreasing exponentially from the diagonal i=j in the GLCM). Unlike Homogeneity2, IDMN normalizes the square of the difference between neighboring intensity values by dividing over the square of the total number of discrete intensity values. JointAverage measures the mean gray level intensity of the i distribution.
2. one GLDM-based features: LowGrayLevelEmphasis measures the distribution of low gray-level values, with a higher value indicating a greater concentration of low gray-level values in the image.
3. one GLRLM-based feature: LongRunHighGrayLevelEmphasis measures the joint distribution of long run lengths with higher gray-level values.
4. one NGTDM feature: Contrast is a measure of the spatial intensity change, but is also dependent on the overall gray level dynamic range. Contrast is high when both the dynamic range and the spatial change rate are high, i.e. an image with a large range of gray levels, with large changes between voxels and their neighbourhood.

#### **Pyradiomics parameter**

# This is an example of settings that can be used as a starting point for analyzing CT data. This is only intended as a

# starting point and is not likely to be the optimal settings for your dataset. Some points in determining better values

# are added as comments where appropriate

# When adapting and using these settings for an analysis, be sure to add the PyRadiomics version used to allow you to

# easily recreate your extraction at a later timepoint:

# ############################# Extracted using PyRadiomics version: <version> ######################################

imageType:

Original: {}

LoG:

sigma: [1.0, 2.0, 3.0, 4.0, 5.0] # If you include sigma values >5, remember to also increase the padDistance.

Wavelet: {}

featureClass:

# redundant Compactness 1, Compactness 2 an Spherical Disproportion features are disabled by default, they can be

# enabled by specifying individual feature names (as is done for glcm) and including them in the list.

shape:

firstorder:

glcm: # Disable SumAverage by specifying all other GLCM features available

- 'Autocorrelation'

- 'JointAverage'

- 'ClusterProminence'

- 'ClusterShade'

- 'ClusterTendency'

- 'Contrast'

- 'Correlation'

- 'DifferenceAverage'

- 'DifferenceEntropy'

- 'DifferenceVariance'

- 'JointEnergy'

- 'JointEntropy'

- 'Imc1'

- 'Imc2'

- 'Idm'

- 'Idmn'

- 'Id'

- 'Idn'

- 'InverseVariance'

- 'MaximumProbability'

- 'SumEntropy'

- 'SumSquares'

glrlm:

glszm:

gldm:

ngtdm:

setting:

# Normalization:

# most likely not needed, CT gray values reflect absolute world values (HU) and should be comparable between scanners.

# If analyzing using different scanners / vendors, check if the extracted features are correlated to the scanner used.

# If so, consider enabling normalization by uncommenting settings below:

normalize: true

normalizeScale: 500 # This allows you to use more or less the same bin width.

# Resampling:

# Usual spacing for CT is often close to 1 or 2 mm, if very large slice thickness is used,

# increase the resampled spacing.

# On a side note: increasing the resampled spacing forces PyRadiomics to look at more coarse textures, which may or

# may not increase accuracy and stability of your extracted features.

interpolator: 'sitkBSpline'

resampledPixelSpacing: [1, 1, 1]

#padDistance: 10 # Extra padding for large sigma valued LoG filtered images

# Mask validation:

# correctMask and geometryTolerance are not needed, as both image and mask are resampled, if you expect very small

# masks, consider to enable a size constraint by uncommenting settings below:

#minimumROIDimensions: 2

#minimumROISize: 50

# Image discretization:

# The ideal number of bins is somewhere in the order of 16-128 bins. A possible way to define a good binwidt is to

# extract firstorder:Range from the dataset to analyze, and choose a binwidth so, that range/binwidth remains approximately

# in this range of bins.

binWidth: 25

# first order specific settings:

voxelArrayShift: 1000 # Minimum value in HU is -1000, shift +1000 to prevent negative values from being squared.

# Misc:

# default label value. Labels can also be defined in the call to featureextractor.execute, as a commandline argument,

# or in a column "Label" in the input csv (batchprocessing)

label: 1

#### **Radiomics reporting guidelines**

**patient**

| **Topic** | **Modality** | **Item** | **Description** | **Manuscript** |
| --- | --- | --- | --- | --- |
| Region of interest [[1]](https://ibsi.readthedocs.io/en/latest/04_Radiomics_reporting_guidelines_and_nomenclature.html#id17) |  | 1 | Describe the region of interest that is being imaged. | Anterior mediastinal mass |
| Patient preparation |  | 2a | Describe specific instructions given to patients prior to image acquisition, e.g. fasting prior to imaging. | Not applicable |
|  |  | 2b | Describe administration of drugs to the patient prior to image acquisition, e.g. muscle relaxants. |  |
|  |  | 2c | Describe the use of specific equipment for patient comfort during scanning, e.g. ear plugs. |  |
| Radioactive tracer | PET, SPECT | 3a | Describe which radioactive tracer was administered to the patient, e.g. 18F-FDG. | Not applicable |
|  | PET, SPECT | 3b | Describe the administration method. |  |
|  | PET, SPECT | 3c | Describe the injected activity of the radioactive tracer at administration. |  |
|  | PET, SPECT | 3d | Describe the uptake time prior to image acquisition. |  |
|  | PET, SPECT | 3e | Describe how competing substance levels were controlled. [[2]](https://ibsi.readthedocs.io/en/latest/04_Radiomics_reporting_guidelines_and_nomenclature.html#id18) |  |
| Contrast agent |  | 4a | Describe which contrast agent was administered to the patient. | Omnipaque 350, GE Healthcare |
|  |  | 4b | Describe the administration method. | intravenously administered at a rate of 3.0 mL/s via a power injector, followed by a 20.0 mL saline flush |
|  |  | 4c | Describe the injected quantity of contrast agent. | 1.5 mL/kg body weight |
|  |  | 4d | Describe the uptake time prior to image acquisition. | 45s |
|  |  | 4e | Describe how competing substance levels were controlled. | Not applicable |
| Comorbidities |  | 5 | Describe if the patients have comorbidities that affect imaging. [[3]](https://ibsi.readthedocs.io/en/latest/04_Radiomics_reporting_guidelines_and_nomenclature.html#id19) | Not applicable |

### Acquisition [[4]](https://ibsi.readthedocs.io/en/latest/04_Radiomics_reporting_guidelines_and_nomenclature.html#id20)

| **Topic** | **Modality** | **Item** | **Description** | **Manuscript** |
| --- | --- | --- | --- | --- |
| Acquisition protocol |  | 6 | Describe whether a standard imaging protocol was used, and where its description may be found. | Supplementary material part 1 |
| Scanner type |  | 7 | Describe the scanner type(s) and vendor(s) used in the study. | Supplementary material part 1 |
| Imaging modality |  | 8 | Clearly state the imaging modality that was used in the study, e.g. CT, MRI. | CT |
| Static/dynamic scans |  | 9a | State if the scans were static or dynamic. | static |
|  | Dynamic scans | 9b | Describe the acquisition time per time frame. | Not applicable |
|  | Dynamic scans | 9c | Describe any temporal modelling technique that was used. |  |
| Scanner calibration |  | 10 | Describe how and when the scanner was calibrated. | Not applicable |
| Patient instructions |  | 11 | Describe specific instructions given to the patient during acquisition, e.g. breath holding. | at the end of inspiratory phase of patients |
| Anatomical motion correction |  | 12 | Describe the method used to minimise the effect of anatomical motion. | breath holding |
| Scan duration |  | 13 | Describe the duration of the complete scan or the time per bed position. | Not applicable |
| Tube voltage | CT | 14 | Describe the peak kilo voltage output of the X-ray source. | 120KV |
| Tube current | CT | 15 | Describe the tube current in mA. | adaptive tube current technology |
| Time-of-flight | PET | 16 | State if scanner time-of-flight capabilities are used during acquisition. | Not applicable |
| RF coil | MRI | 17 | Describe what kind RF coil used for acquisition, incl. vendor. | Not applicable |
| Scanning sequence | MRI | 18a | Describe which scanning sequence was acquired. |  |
|  | MRI | 18b | Describe which sequence variant was acquired. |  |
|  | MRI | 18c | Describe which scan options apply to the current sequence, e.g. flow compensation, cardiac gating. |  |
| Repetition time | MRI | 19 | Describe the time in ms between subsequent pulse sequences. |  |
| Echo time | MRI | 20 | Describe the echo time in ms. |  |
| Echo train length | MRI | 21 | Describe the number of lines in k-space that are acquired per excitation pulse. |  |
| Inversion time | MRI | 22 | Describe the time in ms between the middle of the inverting RF pulse to the middle of the excitation pulse. |  |
| Flip angle | MRI | 23 | Describe the flip angle produced by the RF pulses. |  |
| Acquisition type | MRI | 24 | Describe the acquisition type of the MRI scan, e.g. 3D. |  |
| k-space traversal | MRI | 25 | Describe the acquisition trajectory of the k-space. |  |
| Number of averages/ excitations | MRI | 26 | Describe the number of times each point in k-space is sampled. |  |
| Magnetic field strength | MRI | 27 | Describe the nominal strength of the MR magnetic field. |  |

### Reconstruction [[5]](https://ibsi.readthedocs.io/en/latest/04_Radiomics_reporting_guidelines_and_nomenclature.html#id21)

| **Topic** | **Modality** | **Item** | **Description** | **Manuscript** |
| --- | --- | --- | --- | --- |
| In-plane resolution |  | 28 | Describe the distance between pixels, or alternatively the field of view and matrix size. | Supplementary material part 1 |
| Image slice thickness |  | 29 | Describe the slice thickness. | Supplementary material part 1 |
| Image slice spacing |  | 30 | Describe the distance between image slices. [[6]](https://ibsi.readthedocs.io/en/latest/04_Radiomics_reporting_guidelines_and_nomenclature.html#id22) | Supplementary material part 1 |
| Convolution kernel | CT | 31a | Describe the convolution kernel used to reconstruct the image. | Supplementary material part 1 |
|  | CT | 31b | Describe settings pertaining to iterative reconstruction algorithms. | Supplementary material part 1 |
| Exposure | CT | 31c | Describe the exposure (in mAs) in slices containing the region of interest. | Supplementary material part 1 |
| Reconstruction method | PET | 32a | Describe which reconstruction method was used, e.g. 3D OSEM. | Not applicable |
|  | PET | 32b | Describe the number of iterations for iterative reconstruction. |  |
|  | PET | 32c | Describe the number of subsets for iterative reconstruction. |  |
| Point spread function modelling | PET | 33 | Describe if and how point-spread function modelling was performed. |  |
| Image corrections | PET | 34a | Describe if and how attenuation correction was performed. |  |
|  | PET | 34b | Describe if and how other forms of correction were performed, e.g. scatter correction, randoms correction, dead time correction etc. |  |
| Reconstruction method | MRI | 35a | Describe the reconstruction method used to reconstruct the image from the k-space information. |  |
|  | MRI | 35b | Describe any artifact suppression methods used during reconstruction to suppress artifacts due to undersampling of k-space. |  |
| Diffusion-weigh ted imaging | DWI-MRI | 36 | Describe the b-values used for diffusion-weigh ting. |  |

### Image registration

| **Topic** | **Modality** | **Item** | **Description** | **Manuscript** |
| --- | --- | --- | --- | --- |
| Registration method |  | 37 | Describe the method used to register multi-modality imaging. | Not applicable |

### Image processing

### Data conversion

| **Topic** | **Modality** | **Item** | **Description** | **Manuscript** |
| --- | --- | --- | --- | --- |
| SUV normalisation | PET | 38 | Describe which standardised uptake value (SUV) normalisation method is used. | Not applicable |
| ADC computation | DWI-MRI | 39 | Describe how apparent diffusion coefficient (ADC) values were calculated. |  |
| Other data conversions |  | 40 | Describe any other conversions that are performed to generate e.g. perfusion maps. |  |

### Post-acquisition processing

| **Topic** | **Modality** | **Item** | **Description** | **Manuscript** |
| --- | --- | --- | --- | --- |
| Anti-aliasing |  | 41 | Describe the method used to deal with anti-aliasing when down-sampling during interpolation. | Not applicable |
| Noise suppression |  | 42 | Describe methods used to suppress image noise. |  |
| Post-reconstruc tion smoothing filter | PET | 43 | Describe the width of the Gaussian filter (FWHM) to spatially smooth intensities. |  |
| Skull stripping | MRI (brain) | 44 | Describe method used to perform skull stripping. |  |
| Non-uniformity correction [[7]](https://ibsi.readthedocs.io/en/latest/04_Radiomics_reporting_guidelines_and_nomenclature.html#id23) | MRI | 45 | Describe the method and settings used to perform non-uniformity correction. |  |
| Intensity normalisation |  | 46 | Describe the method and settings used to normalise intensity distributions within a patient or patient cohort. | Normalize: true  NormalizeScale: 500 |
| Other post-acquisitio n processing methods |  | 47 | Describe any other methods that were used to process the image and are not mentioned separately in this list. | Not applicable |

|  |
| --- |

### Segmentation

| **Topic** | **Modality** | **Item** | **Description** | **Manuscript** |
| --- | --- | --- | --- | --- |
| Segmentation method |  | 48a | Describe how regions of interest were segmented, e.g. manually. | manually |
|  |  | 48b | Describe the number of experts, their expertise and consensus strategies for manual delineation. | 2, Y.Z., 6 years’ experience in thoracic imaging diagnosis; Z.Z., 6 years’ experience in thoracic imaging diagnosis |
|  |  | 48c | Describe methods and settings used for semi-automatic and fully automatic segmentation. | Not applicable |
|  |  | 48d | Describe which image was used to define segmentation in case of multi-modality imaging. | Not applicable |
| Conversion to mask |  | 49 | Describe the method used to convert polygonal or mesh-based segmentations to a voxel-based mask. | Not applicable |

### Image interpolation

| **Topic** | **Modality** | **Item** | **Description** | **Manuscript** |
| --- | --- | --- | --- | --- |
| Interpolation method |  | 50a | Describe which interpolation algorithm was used to interpolate the image. | sitkBSpline |
|  |  | 50b | Describe how the position of the interpolation grid was defined, e.g. align by center. | <https://pyradiomics.readthedocs.io/en/v3.0.1/>  (access date 30^th^ April 2022) |
|  |  | 50c | Describe how the dimensions of the interpolation grid were defined, e.g. rounded to nearest integer. | <https://pyradiomics.readthedocs.io/en/v3.0.1/>  (access date 30^th^ April 2022) |
|  |  | 50d | Describe how extrapolation beyond the original image was handled. | <https://pyradiomics.readthedocs.io/en/v3.0.1/>  (access date 30^th^ April 2022) |
| Voxel dimensions |  | 51 | Describe the size of the interpolated voxels. | [1,1,1] |
| Intensity rounding | CT | 52 | Describe how fractional Hounsfield Units are rounded to integer values after interpolation. | <https://pyradiomics.readthedocs.io/en/v3.0.1/>  (access date 30^th^ April 2022) |

###

### ROI interpolation

| **Topic** | **Modality** | **Item** | **Description** | **Manuscript** |
| --- | --- | --- | --- | --- |
| Interpolation method |  | 53 | Describe which interpolation algorithm was used to interpolate the region of interest mask. | <https://pyradiomics.readthedocs.io/en/v3.0.1/>  (access date 30^th^ April 2022) |
| Partially masked voxels |  | 54 | Describe how partially masked voxels after interpolation are handled. | <https://pyradiomics.readthedocs.io/en/v3.0.1/>  (access date 30^th^ April 2022) |

### Re-segmentation

| **Topic** | **Modality** | **Item** | **Description** | **Manuscript** |
| --- | --- | --- | --- | --- |
| Re-segmentation methods |  | 55 | Describe which methods and settings are used to re-segment the ROI intensity mask. | Not applicable |

### Discretisation

| **Topic** | **Modality** | **Item** | **Description** | **Manuscript** |
| --- | --- | --- | --- | --- |
| Discretisation method [[8]](https://ibsi.readthedocs.io/en/latest/04_Radiomics_reporting_guidelines_and_nomenclature.html#id24) |  | 56a | Describe the method used to discretise image intensities. | <https://pyradiomics.readthedocs.io/en/v3.0.1/>  (access date 30^th^ April 2022) |
|  |  | 56b | Describe the number of bins (FBN) or the bin size (FBS) used for discretisation. | 20 |
|  |  | 56c | Describe the lowest intensity in the first bin for FBS discretisation. [[9]](https://ibsi.readthedocs.io/en/latest/04_Radiomics_reporting_guidelines_and_nomenclature.html#id25) | 16 |

### Image transformation

| **Topic** | **Modality** | **Item** | **Description** | **Manuscript** |
| --- | --- | --- | --- | --- |
| Image filter [[10]](https://ibsi.readthedocs.io/en/latest/04_Radiomics_reporting_guidelines_and_nomenclature.html#id26) |  | 57 | Describe the methods and settings used to filter images, e.g. Laplacian-of-Gaussian. | Laplacian-of-Gaussian and wavelets |

### Image biomarker computation

| **Topic** | **Modality** | **Item** | **Description** | **Manuscript** |
| --- | --- | --- | --- | --- |
| Biomarker set |  | 58 | Describe which set of image biomarkers is computed and refer to their definitions or provide these. | In total, 1288 radiomics features were extracted, the specific classifications are as follows: (1) first order statistics and filter-based features(n = 252), (2) shape (n = 14), (3) gray level co-occurrence matrix (GLCM) and filter-based features (n = 308), (4) gray level run length matrix (GLRLM) and filter-based features (n = 224), (5) gray level size zone matrix (GLSZM) and filter-based features (n = 224), (6) gray level dependence matrix (GLDM) and filter-based features (n = 196), and (7) neighbouring gray tone difference matrix (NGTDM) and filter-based features (n = 70). |
| IBSI compliance |  | 59 | State if the software used to extract the set of image biomarkers is compliant with the IBSI benchmarks. [[11]](https://ibsi.readthedocs.io/en/latest/04_Radiomics_reporting_guidelines_and_nomenclature.html#id27) | For the most part, yes. |
| Robustness |  | 60 | Describe how robustness of the image biomarkers was assessed, e.g. test-retest analysis. | test-retest analysis |
| Software availability |  | 61 | Describe which software and version was used to compute image biomarkers. | Pyradiomics (version 3.0.1) |

### Image biomarker computation - texture parameters

| **Topic** | **Modality** | **Item** | **Description** | **Manuscript** |
| --- | --- | --- | --- | --- |
| Texture matrix aggregation |  | 62 | Define how texture-matrix based biomarkers were computed from underlying texture matrices. | <https://pyradiomics.readthedocs.io/en/v3.0.1/>  (access date 30^th^ April 2022) |
| Distance weighting |  | 63 | Define how CM, RLM, NGTDM and NGLDM weight distances, e.g. no weighting. | no weighting |
| CM symmetry |  | 64 | Define whether symmetric or asymmetric co-occurrence matrices were computed. | <https://pyradiomics.readthedocs.io/en/v3.0.1/>  (access date 30^th^ April 2022) |
| CM distance |  | 65 | Define the (Chebyshev) distance at which co-occurrence of intensities is determined, e.g. 1. | 1 |
| SZM linkage distance |  | 66 | Define the distance and distance norm for which voxels with the same intensity are considered to belong to the same zone for the purpose of constructing an SZM, e.g. Chebyshev distance of 1. | 1 |
| DZM linkage distance |  | 67 | Define the distance and distance norm for which voxels with the same intensity are considered to belong to the same zone for the purpose of constructing a DZM, e.g. Chebyshev distance of 1. | 1 |
| DZM zone distance norm |  | 68 | Define the distance norm for determining the distance of zones to the border of the ROI, e.g. Manhattan distance. | Manhattan distance |
| NGTDM distance |  | 69 | Define the neighbourhood distance and distance norm for the NGTDM, e.g. Chebyshev distance of 1. | 1 |
| NGLDM distance |  | 70 | Define the neighbourhood distance and distance norm for the NGLDM, e.g. Chebyshev distance of 1. | 1 |
| NGLDM coarseness |  | 71 | Define the coarseness parameter for the NGLDM, e.g. 0. | 0 |

### Machine learning and radiomics analysis

| **Topic** | **Modality** | **Item** | **Description** | **Manuscript** |
| --- | --- | --- | --- | --- |
| Diagnostic and prognostic modelling |  | 72 | See the TRIPOD guidelines for reporting on diagnostic and prognostic modelling. | YES |
| Comparison with known factors |  | 73 | Describe where performance of radiomics models is compared with known (clinical) factors. | YES |
| Multicollinearity |  | 74 | Describe where the multicollineari ty between image biomarkers in the signature is assessed. | YES |
| Model availability |  | 75 | Describe where radiomics models with the necessary pre-processing information may be found. | YES |
| Data availability |  | 76 | Describe where imaging data and relevant meta-data used in the study may be found. | YES |
